# Diet-Induced Over-Expression of Flightless-I Protein and Its Relation to Flightlessness in Mediterranean Fruit Fly, *Ceratitis capitata*

**DOI:** 10.1371/journal.pone.0081099

**Published:** 2013-12-03

**Authors:** Il Kyu Cho, Chiou Ling Chang, Qing X. Li

**Affiliations:** 1 Department of Molecular Biosciences and Bioengineering, University of Hawaii, Honolulu, Hawaii, United States of America; 2 U.S. Pacific Basin Agricultural Research Center, Hilo, Hawaii, United States of America; Oxford Brookes University, United Kingdom

## Abstract

The Mediterranean fruit fly (medfly), *Ceratitis capitata* is among the most economically important pests worldwide. Understanding nutritional requirement helps rearing healthy medfly for biocontrol of its population in fields. Flight ability is a high priority criterion. Two groups of medfly larvae were reared with two identical component diets except one with fatty acids (diet A) and another without it (diet B). Adults from larvae reared on diet B demonstrated 20±8% of normal flight ability, whereas those from larvae reared on diet A displayed full flight ability of 97±1%. Proteomes were profiled to compare two groups of medfly pupae using shotgun proteomics to study dietary effects on flight ability. When proteins detected in pupae A were compared with those in pupae B, 233 and 239 proteins were, respectively, under- and over-expressed in pupae B, while 167 proteins were overlapped in both pupae A and B. Differential protein profiles indicate that nutritional deficiency induced over-expression of flightless-I protein (fli-I) in medfly. All proteins were subjected to Ingenuity Pathway Analysis (IPA) to create 13 biological networks and 17 pathways of interacting protein clusters in human ortholog. Fli-I, leucine-rich repeat (LRR)-containing G protein-coupled receptor 2, LRR protein soc-2 and protein wings apart-like were over-expressed in pupae B. Inositol-1,4,5-trisphosphate receptor, protocadherin-like wing polarity protein stan and several Wnt pathway proteins were under-expressed in pupae B. These results suggest down-regulation of the Wnt/wingless signaling pathway, which consequently may result in flightlessness in pupae B. The *fli-I* gene is known to be located within the Smith-Magenis syndrome (SMS) region on chromosome 17, and thus, we speculate that nutritional deficiency might induce over-expression of fli-I (or *fli-I* gene) and be associated with human SMS. However, more evidence would be needed to confirm our speculation.

## Introduction

Tephritid fruit flies are economically significant pests worldwide. Its control mainly relies on sterile insect technique (SIT). SIT requires continuous mass-rearing, sterilization of adult flies, and release of sterilized male flies into fields to compete with wild male flies for female flies. A female it mates with a sterile male will not produce offspring. Repeated release of sterile males can effectively suppress the population. The Mediterranean fruit fly (medfly), *Ceratitis capitata*, is one of the most successful SIT controls in tephritids. To rear healthy medfly for field release, research has been focused on understanding of nutrient requirement for a liquid diet.

It was very interesting that we observed an increase in medfly flightlessness when the larvae were fed with a fatty acid deficient liquid diet in the present study. We hypothesized that the flightlessness may be caused by over-expression of flightless I protein (fli-I) in the pupal stage. Insects consume much energy on performing flight, and its maintenance is reliant on adequate and continuous supplies of biofuel and oxygen to flight muscles [1]. Studies on the form and function of insect wings and insect flight muscles have been reported since the 1970s [1]–[4]. Insects can utilize a variety of substrates such as carbohydrates, lipids, and amino acids as energy sources for flight [1], [5]–[6]. Flight functions are related with flightless I gene (*fli-I*) and fli-I. The mutation of *fli-I* causes myofibrillar abnormalities in the indirect flight muscles and, thus, flightlessness [6]–[10]. Fli-I was first identified from *Drosophila melanogaster* mutant that could not fly [11]. The *fli-I* gene is highly conserved across life forms from insect, nematode, mouse, to humans [12]. Fli-I contains a C-terminal gelsolin-like domain and an N-terminal leucine-rich repeat (LRR) domain and is the most highly conserved member of the gelsolin family [13]–[15]. The LRR was discovered in a leucine-rich α2-glycoprotein from human serum [16]. Fli-I is essential for early embryogenesis, the structural organization of indirect flight muscle [17], and intracellular signaling via nuclear hormone receptors [18]. The critical role of fli-I is supported by the finding that *fli-I* deficient mice are embryonic lethal [12]. The human *fli-I* gene is located within the short (p) arm of chromosome 17 of Smith-Magenis syndrome (SMS), also called the 17p-syndrome. This syndrome is a developmental disorder that causes mental retardation, distinctive facial features and behavioral abnormalities [19].

The objectives of this study were to identify differentially expressed proteins and compare protein profiles in pupae of their larvae reared on the standard mill feed diet (diet A) and a fatty acid deficient liquid diet (diet B) and to understand how the responded proteins interact within protein networks in medfly pupae and possible link between nutritional deficiency, fli-I and the human disease SMS. To our knowledge, this is the first study of effects of dietary nutrients on over-expression of fli-I and medfly flight ability.

## Materials and Methods

### Medfly rearing

A Hawaii laboratory strain of medfly was reared at 65% relative humidity and 25°C throughout life cycle. Eggs were collected within 6 h after oviposition and were seeded in the fatty acid-supplemented diet (solid mill feed diet; diet A) and the fatty acid-deficient diet (liquid diet; diet B) [20]–[23] until pupation.

### Flight ability assay

Upon pupation, 100 pupae were collected from each of four lots (400 pupae in total) and placed in a petri dish. The dish was then placed over with a black plastic polyvinyl chloride tube (20 cm in length and 8.5 cm in diameter) of which the inside wall was coated with talcum powder (Hawaii Chemical Co., Honolulu, Hawaii) to prevent emerged flies from crawling, instead of flying, out of the tube. Two pieces of folded cardstock (12×1 cm) were placed inside the tube with the pupae to give emerging adults a room to expand their wings [24]. The tubes were placed in a lighted flight cage (120×120×60 cm) without food or water until all flies had emerged and died, usually 24 d after egg seeding or 4 d after adult emergence. Flies that flied out of the tubes were trapped and killed by the sticky paper hanging above the tubes. Flies being incapable of flying were dead inside the petri dish. Flies were categorized into five groups: 1) unemerged, 2) partially emerged (part of adult body stuck to the puparium), 3) flies with deformed wings, 4) nonflying flies (flies that look normal, but are not capable of flying), and 5) flies capable of flying. Three independent experiments were performed and each experiment had 4 replicates.

### Pupae and protein samples

Five-day old medfly pupae were collected, rinsed with proteomics grade water (Bio-Rad, Hercules, CA), and were then stored at −80°C for later use. Pupae (3×8 g; 8 g were approximately 200 pupae) were incubated with 15 mL of lysis buffer [40 mM Tris-HCl, pH 7.4, 5 mM dithiotreitol (DTT), 1 mM, phenylmethylsufonyl fluoride] and with 750 µL of a protease inhibitor cocktail (Roche Diagnostics, Basel, Switzerland) at ambient temperature for 15 min. Lysates were homogenized on ice using an ultraturrax homogenizer (Cole Parmer, Vernon Hills, IL) for 3 min at an interval of 30 s. The homogenate was then centrifuged at 17,000 *g* for 30 min and the supernatant was obtained by passing through a membrane Econofilter (0.2-mm×25-µm, Agilent, Palo Alto, CA). The supernatant was used for identification of proteins and determination of L-leucine content. Protein concentrations were determined with Coomassie PlusTM protein assay kit (Pierce, Rockford, IL). Images of Coomassie stained gels were obtained on a Bio-Rad densitometer GS-800. The gels were analyzed with PD-Quest (Bio-Rad) to compare protein content from pupae A and pupae B. Statistical analyses were performed with GraphPad software (Bio-Rad).

### One dimensional (1D) SDS-PAGE, protein digestion and peptide extraction

An aliquot of protein extracts from pupae emerged from larvae grown on diet A (1.5 µL) and diet B (2.0 µL) (both 25 µg protein equivalents) was mixed with SDS-PAGE sample buffer (3.0 µL and 4.0 µL, respectively) and heated at 100°C for 5 min. The denatured proteins were separated on 10–20% gradient SDS-PAGE mini gels (9×10 cm, PAGE Gold Precast Gel, Cambrex Bioscience, Rockland, ME) followed by Coomassie dye G-250 staining. Precision plus protein standards (10 µL) (Bio-Rad) were used as protein markers. Each gel lane was cut into 20 even slices, destained with 50% (v/v) acetonitrile (ACN) in 25 mM NH_4_HCO_3_, and then completely dried in a speed-vacuum centrifuge (Eppendorf, Hamburg, Germany) after dehydration with ACN. The dried gel slices were reduced in 50 µL of 10 mM DTT for 45 min at 56°C, alkylated in 50 µL of 55 mM iodoacetamide for 45 min at ambient temperature in the dark. The gel slices were dehydrated with ACN followed by drying in the speed-vacuum centrifuge. After addition of 20 µL of sequencing-grade modified porcine trypsin (20 ng/µL in 50 mM NH_4_HCO_3_), samples were incubated at 37°C overnight. Tryptic digestion was stopped by adding 5 µL of 2% trifluoroacetic acid (TFA). The digested peptides were extracted from each gel slice with 30 µL of water/ACN/TFA (93∶5∶2, v/v/v) twice by sonication for 10 min on ice.

### Two dimensional gel electrophoresis (2D GE)

2D GE was conducted with ReadyStrip IPG (Bio-Rad) using 11 cm pH 3–10 IPG strips, and 10–20% Tris-HCl precast acrylamide gels. Pupae (3×8 g) were ground in liquid N_2_ using a mortar and pestle. An amount of 100 mg of the powdered pupae was dissolved in 1 mL of lysis buffer (4% CHAPS, 8 M urea, 10% Triton-100, 2% bio-lyte, 1 M DTT, a trace amount (2 µL) of DNase I and RNase A, and protease inhibitor mixture) using an ultrasonic cell disruptor (Misonix, Farmingdale, NY) and mixed with 200 µL of rehydration buffer. After centrifugation at 29,774 *g* for 15 min at 4°C, an aliquot of the supernatant (10 µg of solubilized protein) was applied to isoelectric focusing (IEF) using immobiline dry strips (immobilized pH gradient, pH 3–10, 11 cm, linear) according to the instruction (Bio-Rad). IEF of the rehydrated strips was performed in a Protein® IEF Cell (Bio-Rad) with linear ramping of voltage (50 V for 10 h, 100 V for 3 h, 8,000 V for 1 h, 800 V for 10 h for a total of 50,000 Vh). After IEF, strips were equilibrated in 0.375 M Tris-HCl, pH 8.8, 6 M urea, 2% SDS, 20% glycerol with 130 mM DTT for 15 min, and then for 15 min in the same buffer without DTT but with 135 mM iodoacetamide. Equilibrated strips were placed on the top of 20% PAGEs and fixed with 0.5% agarose in a concentrating buffer (62.5 mM Tris-HCl, pH 6.8, 0.1% SDS). The second dimension was run at 300 V and 30 mA/gel for 8 h.

### Protein identification by LC-ion trap mass spectrometry (LC-ITMS) and matrix assisted desorption/ionization-time of flight/time of flight (MALDI TOF/TOF) MS

The digested peptides of which the proteins were separated on 1D gel were analyzed on a Dionex UltiMate™ 3000 nano LC interfaced with an esquireHCT^ultra^ ion trap mass spectrometer (Bruker Daltonics, Billerica, ME) in nanoelectrospray mode with a PicoTip Emitter (360 µm O.D., 20 µm I.D., 10 µm tip I.D., New Objective, Woburn, MA) according to the procedure previously published [24], [25]. The nano-LC column was C_18_ PepMap 100 (3 µm film thickness, 75 µm I.D. ×15 cm, Dionex Corp., Sunnyvale, CA) at a flow of ca. 180 nL/min. MS/MS spectra were interpreted with Mascot (Matrix Science, London, UK) via Biotools 2.2 software (Bruker). Peak lists for protein identification were created by Compass 1.3 of Bruker for esquireHCT^ultra^. Peptide mass fingerprint (PMF) searches and sequence alignments were performed in Swiss-Prot through the Mascot sever with database of *Drosophila melanogaster*. UniProt classification was used to search cellular functions of identified proteins. Peptides were assumed to be monoisotopic, oxidized at methionine residues and carbamidomethylated at cysteine residues. Up to one missed trypsin cleavage was allowed, although matches that contained any missed cleavages were not noticed. Peptide mass and MS/MS tolerances were set at ±1.0 and ±0.8 Da, respectively. Probability-based molecular weight search (MOWSE) scores were estimated and were reported as: 10×log_10_ (*p*), where *p* is the absolute probability. Scores in Mascot larger than the MOWSE score at *p* = 0.05 were considered statistically significant, meaning that the probability of the match being a random event is lower than 0.05. The false-positive rate (FPR) was estimated according to the method of Elias et al. [26] and was smaller than 1% [FPR  =  FP/(FP + TP), where FP is the number of FPR hits; TP is the number of true-positive hits]. Only proteins identified with at least two peptide hits, with each peptide containing two tryptic termini, were accepted. In addition, the MS/MS spectra of all positively identified peptides were manually confirmed twice. Proteins of pupae A and B were identified in triplicate and are summarized (Tables S1 and S2).

MALDI TOF/TOF MS was used to verify proteins identified with LC-ITMS. Protein spots on 2D GE were excised manually with a one touch spot picker (The Gel Company, San Francisco, CA), followed by on-gel digestion of proteins in the same matter as proteins on 1D SDS-PAGE. Trypsin-digested peptides (1 µL) from each spot were mixed with an equal volume of α-cyano-4-hydroxycinnamic acid, and then were applied directly onto a metal MTP 386 target plate. The samples were analyzed on a Bruker UltraflexIII MALDI-TOF/TOF mass spectrometer equipped with a Smartbeam™ laser.

Proteins detected in pupae B were compared with those in pupae A (control). Detection of a protein in pupae B but not in pupae A is referred to as over-expressed or over-represented, whereas a protein undetected in pupae B but detected in pupae A is referred to as under-expressed or under-represented.

### Pathway and network analysis

Proteins identified based on mascot search with *D. melanogaster* database were interpreted as human ortholog in IPA (Ingenuity System, Redwood City, CA). Accession numbers of detected proteins (UniProt/SwissProt Ids) were listed in MS Excel and imported into IPA to create canonical pathways and networks of interacting proteins. Protein IDs uploaded into IPA were converted to the corresponding human Entrez Gene symbols for display in pathways and networks. The data were analyzed in the context of humans (IPA ID No.: 7571344).

### Quantification of L-leucine by LC-mass selective detector (LC-MSD)

L-Leucine was analyzed on an Agilent 1100 LC-MSD system equipped with a binary pump and an Atlantis HILIC silica column (4.6×250 mm, 5 µm; Waters, Milford, MA) housed in a thermostatic compartment at 20°C. The mobile phase was set linear gradient starting from 100% ACN and ending at 30% 6.5 mM ammonium acetate pH 5.5 and 70% ACN after 40 min at a constant flow rate of 0.4 mL/min. The injection volume was 10 µL. The MS was operated in electrospray ionization mode. The drying gas was nitrogen at a flow rate of 10.0 L/min at 350°C, creating a nebulizing pressure of 40 psi. The capillary voltage was set at 5 kV. Mass spectra were collected in positive ion mode with selected ion monitoring of m/z fragments at 131.2 amu with a fragmentation voltage of 150 V. Leucine in pupal samples was identified by comparison of retention times and MS spectra against L-leucine standard. Leucine concentrations were measured with calibration curves of a mixture of L- and D-leucine standards at concentrations of 2–200 µg/mL. All standards and samples were diluted with ACN/water (1/1, v/v) as appropriate.

## Results and Discussion

### Dietary effects on flight ability of medfly

**Figure 1:**
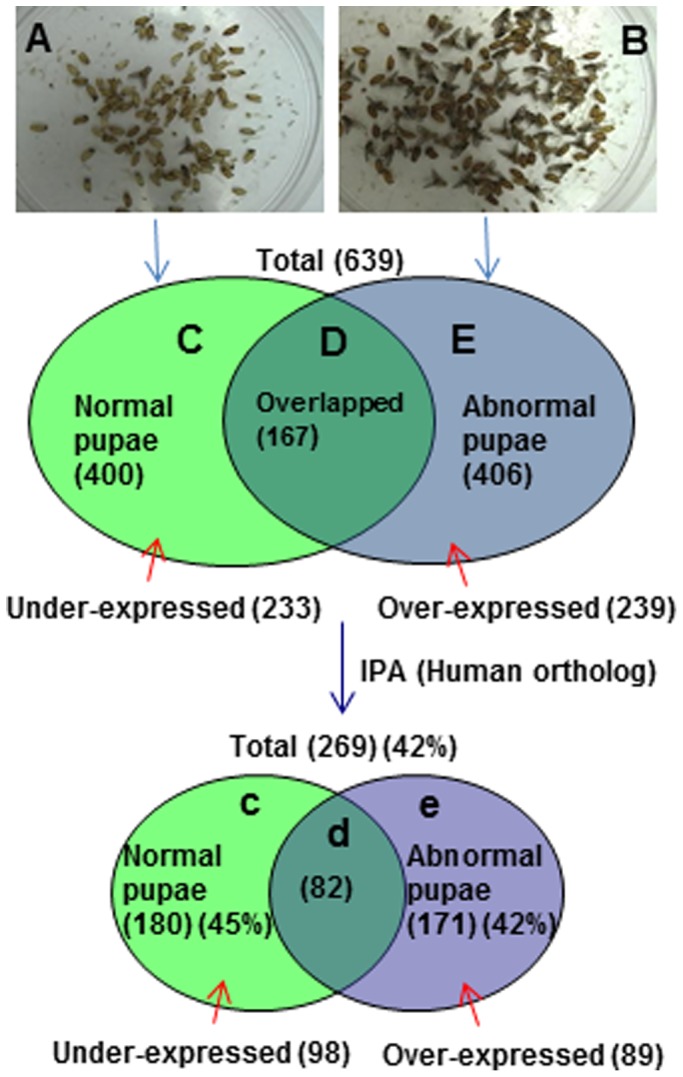
Photograph of medfly flight ability assay remains (A–B), number of protein identified (C–E), and number of proteins mapped to human ortholog via IPA. **A** and **B**: are pupal emergence remains, which the larvae were reared on diet A (standard mill feed diet) and diet B (liquid diet), respectively. **C**, **D** and **E** are Venn diagrams showing numbers of proteins differentially identified in the parentheses, of which 167, 233 and 239 proteins were overlapped, under-expressed and over-expressed, respectively. **c**, **d** and **e** are Venn diagrams showing 269 of the 639 identified proteins successfully mapped in 13 biological networks and 17 pathways of interacting protein clusters by IPA (human database).

When medfly larvae were reared on a conventional mill feed diet (designated as diet A) and a fatty acid deficient liquid diet (designated as diet B), full flight ability was displayed in 97±1% adult flies from diet A, but only 20±8% from diet B (Proc ANOVA; *P*<0.0001, df = 1, 7, F = 96.40) (Figure 1A, B). The results signify the diet changes affect flight ability of medfly. Recent studies showed essential dietary fatty acids are necessary for normal development of the eggs, larvae [27], and adult flies of *Bactrocera dorsalis* [28].

### Differential expression of proteins in pupae A and B

The LC-ITMS analyses showed a large difference in protein profiles between pupae A (normal) reared on diet A and pupae B (abnormal) reared on diet B (Figure 1C–E). A total of 400 proteins (Table S1) and 406 proteins (Table S2) were detected in pupae A and B, respectively, while 167 of these proteins were detected in both pupae A and B. Mascot search and differential comparison of the proteins showed that 239 proteins (Table S3) were detected in pupae B, but not in pupae A (referred to as over-expressed proteins in pupae B), while 233 proteins (Table S4) were detected in pupae A, but not in pupae B (referred to as under-expressed proteins in pupae B).

#### Over-expressed proteins

Among the 239 over-expressed proteins were fli-I, LRR-containing G protein-coupled receptor 2, LRR protein soc-2, paramyosin, tyrosine protein kinase Fps85D, angiotensin-converting enzyme-related protein, spectrin beta chain, fructose-bisphosphate aldolase, glyceraldehyde-3-phosphate dehydrogenase 2, ring finger protein, E3 ubiquitin-protein ligase Su (dx) and E3 ubiquitin-protein ligase highwire involved in the notch signaling pathway, imaginal disc-derived wing morphogenesis and the Ubiquitin conjugation pathway (Table S3). Protein wings apart-like, stubble-stubbloid protein, serine hydrolase, retrovirus-related polyprotein and maternal protein tudor were also detected in only pupae B. These proteins are related to chromosome partitioning, hydrolysis, detoxification and differentiation (Oogenesis). The detected proteins relevant to transcription regulation include protein tamozhennic, protein spint, polycomb protein Asx, alpha-adaptin, protein abrupt, transcription initiation factor IIF, and protein male-specific lethal 3. Abnormal spindle protein, mitosis initiation protein and centrosomin were detected in pupae B. These proteins are related to cell division and actin filament reorganization during cell cycle. Protein slit, tyrosine-protein kinase transmembrane receptor R and netrin-A that are involved in neurogenesis were over- expressed in pupae B.

#### Under-expressed proteins

Inositol-1, 4, 5-trisphosphate receptor (InsP_3_R), protocadherin-like wing polarity protein stan (starry night protein) and several Wnt pathway proteins were under-expressed in pupae B in comparison with pupae A (Table S4). InsP_3_R gene (*itpr*) is essential for the development of the flight circuit during pupation [29]. In particular, InsP3R is indispensable on development of the neural circuit that functions as the central pattern generator for air puff-induced flight [30]. Starry night protein that is a component of the fz signaling pathway controls wing tissue polarity and the Wnt receptor signaling pathway [31].

### Relations between fli-I over-expression and flightlessness

Fli-I, LRR-containing G protein-coupled receptor 2, LRR protein soc-2 and protein wings apart-like were over-expressed in pupae B (Figure S1 and Table S3). They were detected with LC-ITMS in the second gel slice with MWs between approximately 144 and 185 KD (Figure S1 III). However, they were not detected in pupae A. Five tryptic peptides were identified and matched with the fli-I amino acid sequence in the database with 20% coverage and a Mascot score of 36 (Table S2). These five tryptic peptides were further fragmented in MS/MS mode to confirm their amino acid sequences (Figure S1, II). Differential detection of fli-I in pupae B, but not pupae A was also confirmed with 2D GE (Figure S2). The analysis of the 2D GE images showed large difference in the protein scatter plot between pupae A and B (75% of match rate, not shown). In addition to fli-I, over-expression of Hsp70Ab, paramysosin, LRR protein soc-2, LRR containing G protein-coupled receptor 2, E3 ubiquitin-protein ligase Su and protein wing apart-like was found in pupae B with 2D GE and MALDI-TOF/TOF MS analysis (Figure S2, Table S6), which agreed with the results of LC-ITMS (Table S3). The results indicate that induction of fli-I in pupae B by nutritional effects may link with medfly flightlessness. It is known that over-expression of fli-I causes flightlessness in *D. melanogaster* [6]–[12]. The results of the present study showed that the fatty acid deficient liquid diet caused the medfly flightlessness, which was related to fli-I over-expression in pupae B. To our knowledge, this is the first observation of relationships between nutritional deficiency, fli-I over-presentation and medfly flightlessness.

### Protein networks

**Figure 2:**
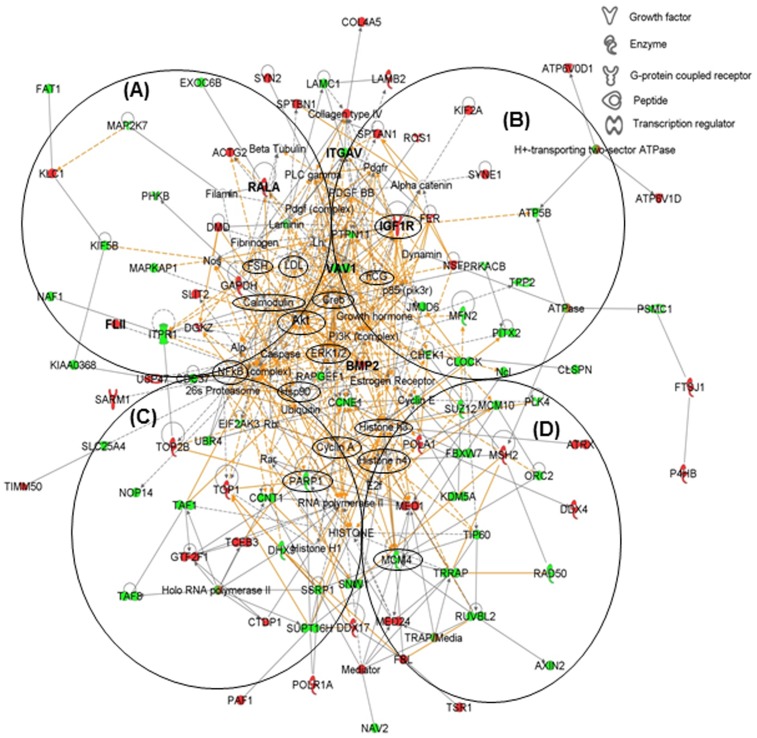
Biological networks of differentially expressed proteins mapped via IPA which consist of the 4 highest scored networks of gene expression (A), cellular assembly and organization (B), cell morphology (C), and cell cycle (D). Small circles identify proteins that serve as hubs to interconnect groups of proteins to form protein clusters of pathways. Proteins in red are the over-expressed, while proteins in green are under-expressed. Solid lines represent direct relationships. Lines connecting the proteins indicate known interrelationships from the IPA database.

Out of the 233, 239 and 167 of correspondingly under-expressed, over-expressed and overlapped proteins, 98, 89 and 82 proteins (Figure 1, Table S5), respectively, were mapped in 13 biological networks and 17 pathways of interacting protein clusters according to the identifiers' HomoloGene to the ortholog information in the Ingenuity Knowledge Base (IKB). The biological network of differentially expressed proteins shows inter-relationships and relevant signaling pathways. Medfly proteins were interpreted as in human ortholog in IPA and the resulted networks would be for humans. One overall network was merged from the 4 highest scored networks of gene expression, cellular assembly and organization, cell morphology, and cell cycle (Figure 2). Thirty seven hub proteins in small circles interconnected groups of proteins to form protein clusters of pathways (Figure 2).

Among 13 over-expressed protein hubs (Figure 2, Table S5), v-ral simian leukemia viral oncogene homolog A (RALA) functions as a negative regulation of JNK cascade. Insulin-like growth factor 1 receptor (IGF1R) is a transmembrane receptor in plasma membrane. Bone morphogenetic protein 2 (BMP2) is associated with BMP signaling pathway. Fer tyrosine kinase (FER) is actin filament bundle assembly. N-Ethylmaleimide-sensitive factor (NSF) and glyceraldehyde-3-phosphate dehydrogenase (GAPDH) are related to glycolysis. Mediator complex subunit 1 (MED1) regulates transcription from RNA polymerase II promoter. DEAD (Asp-Glu-Ala-Asp) box polypeptide 17 (DDX17) is related to RNA-mediated gene silencing. General transcription factor IIF (CTF2F1) acts positive regulation of transcription. Over-expressed proteins that include FLII (i.e., fli-I), topoisomerase 1 (TOP1), sterile α and TIR motif containing 1 (SARM1), eye-specific diacylglycerol kniase (DGKZ), E3 ubiquitin-protein ligase nedd-4 (NEDD4) in pupae B have direct relationships with the nuclear factor of kappa light polypeptide gene enhancer of activated B cell (NF-*k*B) complex that was detected in both pupae A and B. NF-*k*B plays a key role in regulating the immune response to infection since kappa light chains are components of immunoglobulins [32].

Among 11 under-expressed hub proteins (Figure 2, Table S5), integrin alpha V (ITGAV) is related to cell adhesion. Vav 1 guanine nucleotide exchange factor (VAV1) is associated with the actin filament organization. Inositol 1,4,5-trisphosphate receptor (ITPR1) acts as the second messenger that mediates the release of intracellular calcium or calcium transport. Checkpoint kinase 1 (CHEK1), Cyclin E (CCNE1), cell division cycle 37 homolog (CDC37) and TATA box binding protein (TBP)-associated factor (TAF1) are associated with cell cycle. Clock homolog (CLOCK) is related to cellular response to the circadian regulation of gene expression. Rap guanine nucleotide exchange factor 1 (RAPGEF1) is related to the Ras protein signal transduction. Suppressor of zeste 12 homolog (SUZ12) is related to the dendrite morphogenesis. Minichromosome maintenance complex component 10 (MCM10) is related to DNA replication. Eukaryotic translation initiation factor 2-alpha kinase 3 (EIF2AK3) is related to stress response. Poly (ADP-ribose) polymerase 1 (PARP1) has a function on chromatin modification. Cyclin-T 1 (CCNT1) is related to the actin filament organization. Transformation/transcription domain-associated protein (TRRAP) and suppressor of Ty 16 homolog (SUPT16H) have a role of transcription regulation. DEAH (Asp-Glu-Ala-His) box polypeptide 9 (DHX9) is related to axon extension. Structure specific recognition protein 1 (SSRP1) is related to DNA damage or DNA repair.

### Differential expression and regulation of proteins in Wnt signaling pathway

**Figure 3:**
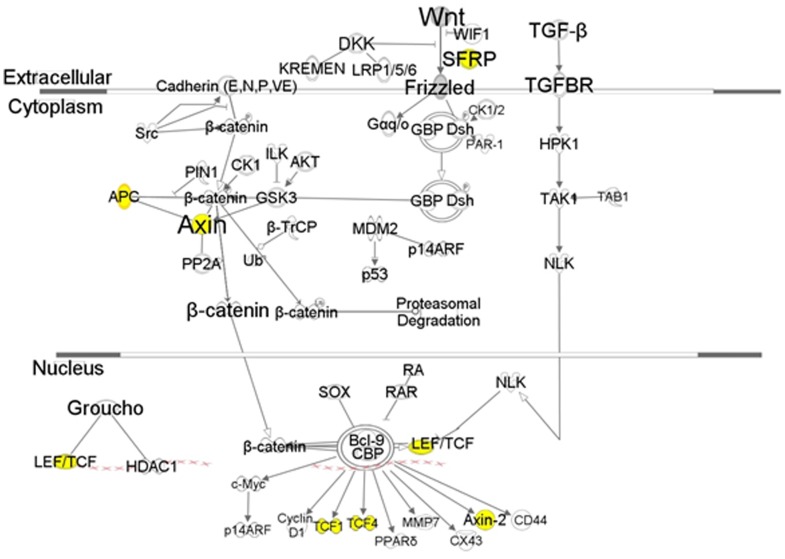
Canonical overview of Wnt signaling. AXlN1, CELSR1, EXT1, NDST2, RUVBL2, SPEN, and WNT5B were under-expressed in pupae B (liquid diet). Yellow marked proteins are encoded by genes that are tumor suppressor genes.

Axin (AXIN1), protocadherin-like wing polarity protein stan (CELSR1), bifunctional heparan sulfate N-deacetylase (NDST2), RuvB-like helicase 2 (RUVBL2), protein Wnt-5 (WNT5B), exostosin-1 (EXT1) and protein split ends (SPEN), which are related to the Wnt signaling pathway, were detected in pupae A and mapped in the network (Figure 2). However, these proteins were not observed in pupae B. Exostosin-2 and protein BCL9 were detected in pupae B even though they were not mapped into the network (Table S3). Apolipophorins (RFABG) and frizzled-2 (FZ2) were detected in both pupae A and B. Both proteins are related to the Wnt/ß-catenin signaling pathway (Figure 3), which comprises a large family of highly conserved growth factors that are responsible for important developmental and homeostatic processes throughout the animal kingdom [33]. In general, Wnt signaling regulates cell proliferation. Notch-inducible signaling molecules modulate cell proliferation in wings. Wnt signaling is required to support cell survival and to promote cell proliferation during the rapid phase of wing disc growth. Cells deprived of Wnt signaling die as a result of cell competition and apoptosis. In the absence of cell competition, loss of Wnt signaling still results in cell death, thus supporting the idea that Wnt signaling functions as a survival factor to the cells in wings [34]. Misregulation of the Wnt pathway can lead to a variety of abnormalities and degenerative diseases. Zinc finger protein 2 (imaginal disc-derived wing morphogenesis), G protein-coupled receptor kinase 1 (specifically phosphorylates the activated forms of G protein-coupled receptors) and putative gustatory receptor 98a (G-protein coupled receptor protein signaling pathway) were under-expressed in pupae B (Table S4).

### Depression of Wnt signaling by fli-I

Wnt signaling is mediated by ß-catenin (Figure 3). ß-Catenin dependent transcription is known to be activated by exogenous fli-I LRR associated protein 1 (FLAP-1) and depressed by fli-I [33]. Over-expression of fli-I can cause developmental defects, such as medfly flightlessness (Figure 1, Figure S1), by causing deregulation of the Wnt/ß-catenin pathway [35]. The finding suggests that over-expression of fli-I depressed Wnt signaling and subsequently may result in flightlessness.

### Diet-induced fli-I over-expression in medfly suggests a possible link between nutrition deficiency and the Smith-Magenis syndrome in humans

In addition to the differential detection of fli-I in pupae B but not in pupae A, the concentrations of L-leucine determined by LC-MSD were 0.21±0.01 and 0.23±0.01 mg/g (wet pupae body weight) in pupae A and B, respectively, which were confirmed with Fourier Transform infrared spectroscopy (Figure S3) and LC-fluorescence analyses (Figure S4). It is noteworthy that an LRR domain is part of fli-I [13]–[15]. Interestingly, adult medflies whose pupae had a high leucine content had an increased flightlessness rate (Figure S4). The *fli-I* in humans encodes fli-I and is located within the SMS region on chromosome 17 [19]. Human fli-I is similar to a *D. melanogasters* protein involved in early embryogenesis and the structural organization of indirect flight muscle. It is not known whether the phenomenon of fatty acid deficiency-induced fli-I over-expression and incapable flying observed in the present study would be species-specific to medfly or not.

## Conclusion

This study showed effects of nutritional deficiency on over-expression of fli-I and medfly flightlessness. The adult medflies emerged from pupae whose larvae were reared on a fatty acid deficient liquid diet looked normal, but could not fly, whereas the control flies emerged from pupae whose larvae were reared on the standard mill feed diet looked normal and had normal flight ability. The two groups of larvae showed large differential differences of protein profiles. In comparison, fli-I, LRR-containing G protein-coupled receptor 2, LRR protein soc-2 and protein wings apart-like were over-expressed in pupae from larvae fed with the fatty acid deficient liquid diet. The results signify that nutritional deficiency induces fli-I over-expression in medfly. Fli-I over-expression may depress the Wnt signaling, as indicated by under-expression of several Wnt pathway proteins, and thus cause flightlessness. As the *fli-I* gene is highly conserved across life forms from insects to humans and SMS is due to an abnormality in the short (p) arm of chromosome 17 where *fli-I* gene is located, we would postulate that nutritional deficiency might be associated with human SMS.

## Supporting Information

Table S1 A list of 400 proteins detected in pupae A whose larvae were reared in the standard mill feed diet.
(DOC)

Table S2 A list of 406 proteins detected in pupae B whose larvae were reared in the liquid diet.
(DOC)

Table S3 A list of 239 over-expressed proteins detected in pupae B whose adult flies showed a low flight rate.
(DOC)

Table S4 A list of 233 under-expressed proteins in pupae B whose adult flies showed a low flight rate.
(DOC)

Table S5 Medfly protein identifiers (269) converted to the corresponding Entrez Genes in Human ortholog.
(DOC)

Table S6 Over-expressed proteins in pupae B reared with the liquid diet, which were identified by MALDI-TOF/TOF and de *novo sequence* analysis.
(DOC)

Figure S1 **Overlay of extracted LC-ITMS total ion chromatograms of tryptic peptides from proteins in the second gel slice from pupae A and B (Ia and Ib, respectively), MS (IIa) and MS/MS (IIb) spectra of a fli-I tryptic peptide, and 1D SDS-PAGE image of proteins extracted from pupae A (IIIa) and B (IIIb).**
(DOC)

Figure S2 **Images of 2D GE of proteins extracted from pupae A (A) and B (B).**
(DOC)

Figure S3 **Stacked spectra of L-leucine standard (260 ppm) in water and extracts of pupae A (control) and pupae B which the latter showed a low flight rate.**
(DOC)

Figure S4 **LC-fluorescence chromatograms of L-leucine in medfly pupal extracts.**
(DOC)
