# Supplementary material for: Diet-Induced Over-Expression of Flightless-I Protein and Its Relation to Flightlessness in Mediterranean Fruit Fly, Ceratitis capitata
Source: PLoS One. 2013 Dec 3;8(12):e81099. doi: 10.1371/journal.pone.0081099 (PMC3849048; doi:10.1371/journal.pone.0081099)
Supplement: Table S1 — A list of 400 proteins detected in pupae A whose larvae were reared in the standard mill feed diet. (DOC) [file pone.0081099.s001.doc]

**Supporting Information (SI)**

**Diet-induced over-expression of flightless-I protein and its relation to flightlessness in Mediterranean fruit fly, *Ceratitis capitata***

Il Kyu Cho^1^, Chiou Ling Chang^2^ and Qing X. Li^1^*

^1^ Department of Molecular Biosciences and Bioengineering, University of Hawaii, Honolulu, Hawaii, USA.

^2^ U.S. Pacific Basin Agricultural Research Center, Hilo, Hawaii, USA.

**Table S1**. **A list of 400 proteins detected in pupae A whose larvae were reared in the standard mill feed diet.**. The LC-MS/MS data were matched with *Drosophila melanogaster* database via MASCOT for the sequence alignments.

| No. | Protein names | No. of matched peptides | Mascot Scores (p=0.05) | Accession numbers | Biological functions |
| --- | --- | --- | --- | --- | --- |
| 1 | Zinc finger protein hangover | 7 | 73 (33) | Q9VXG1 | Response to ethanol |
| 2 | Bifunctional arginine demethylase and lysyl-hydroxylase PSR | 5 | 70 (33) | Q9VD28 | Dioxygenase |
| 3 | Short spindle protein 4 | 5 | 68 (33) | A1ZAU8 | Cell cycle |
| 4 | Protein pecanex | 6 | 66 (33) | P18490 | Differentiation/Neurogenesis |
| 5 | E3 ubiquitin-protein ligase HERC2 | 5 | 65 (33) | Q9VR91 | Ubl conjugation pathway |
| 6 | Neuropathy target esterase sws | 5 | 62 (33) | B3NY03 | Neurogenesis/Endoplasmic reticulum |
| 7 | Titin | 15 | 61 (33) | Q9I7U4 | Cell cycle |
| 8 | Interference hedgehog | 5 | 61 (33) | B4GKZ8 | Smoothened signaling pathway |
| 9 | Protein split ends | 9 | 63 (26) | Q8SX83 | Wnt receptor signaling pathway |
| 10 | PERQ amino acid-rich with GYF domain-containing protein CG11148 | 4 | 60 (33) | Q7KQM6 | Belongs to the PERQ family |
| 11 | Nuclear factor NF-kappa-B p110 subunit | 4 | 59 (33) | Q94527 | Immune response |
| 12 | Serine/threonine-protein kinase PLK4 | 4 | 57 (33) | O97143 | Centriole replication |
| 13 | Histone H1 | 8 | 54 (33) | P02255 | Nucleosome assembly |
| 14 | Protein KIAA0664 | 4 | 53 (33) | B4GAM1 | [KIAA0664/TIF31 family](http://www.uniprot.org/uniprot/?query=family:"KIAA0664%2FTIF31+family").c |
| 15 | G protein-coupled receptor kinase 1 | 4 | 35 (29) | P32865 | Specifically phosphorylates the activated forms of G protein-coupled receptors |
| 16 | Protein cubitus interruptus | 4 | 51 (33) | P19538 | Cuticle pattern formation |
| 17 | Mitoferrin | 3 | 51 (33) | Q9VAY3 | Mitochondrial iron ion transport |
| 18 | VPRBP-like protein | 4 | 51 (33) | Q9W2F2 | Phosphoprotein |
| 19 | MPN domain-containing protein CG4751 | 4 | 50 (33) | Q9VKJ1 | Probable protease |
| 20 | T-complex protein 1 subunit alpha | 6 | 50 (33) | P12613 | Mitotic spindle organization |
| 21 | Helicase domino | 5 | 49 (33) | Q9NDJ2 | Cell cycle |
| 22 | Protein lin-54 | 5 | 49 (33) | A1Z9E2 | Adult lifespan |
| 23 | Female-specific protein transformer | 5 | 49 (33) | P11596 | Cell differentiation |
| 24 | Protein chiffon | 5 | 48 (33) | Q9NK54 | DNA replication/ |
| 25 | Protein extra-macrochaetae | 3 | 56 (33) | P18491 | R8 cell fate commitment |
| 26 | Protein daughter of sevenless | 3 | 47 (33) | Q9VZZ9 | signaling from various receptor tyrosine kinases such as sevenless |
| 27 | Neurobeachin | 4 | 36 (29) | Q9W4E2 | Compound eye cone cell differentiation/eye photoreceptor cell development |
| 28 | Protein disks lost | 2 | 46 (33) | Q8T626 | Cellular process |
| 29 | RNA-binding protein orb2 | 3 | 46 (33) | Q9VSR3 | Long-term memory |
| 30 | Axin | 5 | 46 (33) | Q9V407 | Wnt signaling pathway |
| 31 | Transmembrane protein 41 | 3 | 45 (33) | Q9VX39 | Integral to membrane |
| 32 | Mediator of RNA polymerase II transcription subunit 26 | 4 | 45 (33) | Q29CV2 | Transcription regulation |
| 33 | Mediator of RNA polymerase II transcription subunit 13 | 6 | 45 (33) | Q7KTX8 | Transcription regulation |
| 34 | Eukaryotic translation initiation factor 3 subunit B | 3 | 54 (33) | B4J6D5 | Protein biosynthesis |
| 35 | Eukaryotic translation initiation factor 3 subunit D-2 | 5 | 44 (33) | B4QT07 | Protein biosynthesis |
| 36 | Origin recognition complex subunit 2 | 4 | 44 (33) | Q24168 | DNA replication/nucleus |
| 37 | Putative epidermal cell surface receptor | 6 | 44 (33) | Q04164 | Instar larval development |
| 38 | Tyrosine-protein phosphatase corkscrew | 3 | 44 (33) | Q24708 | Protein amino acid dephosphorylation |
| 39 | Protein ELYS | 4 | 44 (33) | Q9VWE6 | [Phosphoprotein](http://www.uniprot.org/keywords/KW-0597) |
| 40 | Maternal protein tudor | 5 | 44 (33) | P25823 | Differentiation |
| 41 | Myosin heavy chain, non-muscle | 6 | 43 (33) | Q99323 | Malpighian tubule morphogenesis |
| 42 | Tyrosine-protein kinase Abl | 5 | 43 (33) | P00522 | Axon guidance |
| 43 | Negative elongation factor E | 2 | 43 (33) | Q95ZE9 | Transcription regulation/Nucleus |
| 44 | DNA polymerase subunit gamma-1 | 4 | 42 (33) | Q27607 | DNA replication |
| 45 | Histone-lysine N-methyltransferase, H3 lysine-79 specific | 5 | 54 (33) | Q8INR6 | Chromatin silencing at telomere |
| 46 | Serine/threonine-protein kinase Genghis Khan | 4 | 42 (33) | Q9W1B0 | Actin polymerization or depolymerization |
| 47 | Cell division cycle 2-like protein kinase CG7597 | 4 | 41 (33) | Q9VP22 | Protein amino acid phosphorylation |
| 48 | Ring canal kelch protein | 5 | 41 (33) | Q04652 | Differentiation/Oogenesis |
| 49 | Bloom syndrome protein | 4 | 41 (33) | Q9VGI8 | DNA replication |
| 50 | Diacylglycerol kinase 1 | 5 | 41 (33) | Q01583 | Activation of protein kinase C activity by G-protein coupled receptor protein signaling pathway |
| 51 | cAMP-specific 3',5'-cyclic phosphodiesterase | 3 | 40 (33) | P12252 | Axon extension |
| 52 | Nucleolar protein 14 | 3 | 40 (33) | Q9VEJ2 | Ribosome biogenesis/rRNA processing |
| 53 | Protein sickie | 8 | 73 (33) | Q9VIQ9 | Immune response/Innate immunity |
| 54 | Histone-lysine N-methyltransferase Mes-4 | 4 | 39 (33) | Q8MT36 | Histone methyltransferase |
| 55 | Plexin-B | 4 | 41 (32) | Q9V4A7 | Differentiation/Oogenesis |
| 56 | Inhibitor of nuclear factor kappa-B kinase | 2 | 39 (33) | Q9VEZ5 | Immune response/Innate immunity |
| 57 | Tyrosine-protein phosphatase corkscrew | 3 | 39 (33) | P29349 | Epidermal growth factor receptor signaling pathway |
| 58 | Ubiquitin carboxyl-terminal hydrolase 36 | 6 | 41 (32) | B4KXJ5 | Ubl conjugation pathway |
| 59 | 60 kDa heat shock protein | 4 | 43 (33) | Q9VPS5 | Protein folding |
| 60 | Histone-lysine N-methyltransferase CG1716 | 6 | 39 (33) | Q9VYD1 | Ecdysone receptor-mediated signaling pathway |
| 61 | Muscle-specific homeobox protein tinman | 12 | 39 (33) | P22711 | Cardiac muscle cell differentiation |
| 62 | La-related protein CG11505 | 5 | 39 (33) | Q9I7T7 | [RNA binding](http://www.ebi.ac.uk/ego/DisplayGoTerm?id=GO:0003723) |
| 63 | Cadherin-87A | 2 | 39 (33) | Q9VGG5 | Cell adhesion |
| 64 | Putative 115 kDa protein in type-1 retrotransposable element R1DM | 4 | 31 (29) | P16425 | RNA-dependent DNA replication |
| 65 | Conserved oligomeric Golgi complex | 3 | 38 (33) | Q9VJD3 | Differentiation |
| 66 | Homeotic protein spalt-major | 6 | 32 (29) | P39770 | [Transcription regulation](http://www.uniprot.org/keywords/KW-0805) |
| 67 | Dual specificity mitogen-activated protein kinase kinase hemipterous | 2 | 38 (33) | Q23977 | JNK (c-Jun N-terminal kinase) cascade |
| 68 | Centaurin-gamma-1A | 12 | 37 (33) | Q9NGC3 | Regulation of ARF GTPase activity |
| 69 | Protein aurora borealis | 2 | 37 (33) | Q9VVR2 | Cell cycle |
| 70 | Glutamate dehydrogenase | 2 | 37 (33) | P54385 | NADH oxidation |
| 71 | Serine/threonine-protein phosphatase 2B catalytic | 3 | 37 (33) | P48456 | Neurotransmitter secretion |
| 72 | Microtubule-associated protein futsch | 9 | 72 (32) | Q9W596 | Axon cargo transport/Cytoplasm |
| 73 | Circadian locomoter output cycles protein kaput | 2 | 36 (33) | O61735 | Behavioral response to cocaine |
| 74 | Homeotic protein female sterile | 4 | 36 (33) | P13709 | Multicellular organismal development |
| 75 | Kinesin heavy chain | 2 | 36 (33) | P17210 | Axon cargo transport/microtubule |
| 76 | Protein zer-1 | 2 | 36 (33) | Q9W0E8 | Ubl conjugation pathway/Leucine-rich repeat Repeat |
| 77 | Protein FAM21 | 3 | 35 (33) | A1ZBW7 | Phosphoprotein |
| 78 | Eukaryotic translation initiation factor 3 | 5 | 35 (33) | B3LZN3 | Protein biosynthesis |
| 79 | Protein spire | 3 | 35 (33) | Q29KT5 | Multicellular organismal development |
| 80 | Polycomb protein Su(z)12 | 4 | 40 (33) | Q9NJG9 | Dendrite morphogenesis |
| 81 | RRP12-like protein | 4 | 38 (33) | Q9VYA7 | Phosphoprotein |
| 82 | Protein matrimony | 2 | 34 (33) | P83733 | Cell cycle |
| 83 | Cyclic AMP response element-binding protein A | 2 | 34 (33) | P29747 | Chitin-based larval cuticle pattern formation |
| 84 | LIM domain kinase 1 | 3 | 34 (29) | Q8IR79 | Actin cytoskeleton organization |
| 85 | Transcription-associated protein 1 | 5 | 42 (33) | Q8I8U7 | Transcription regulation |
| 86 | Host cell factor | 2 | 34 (33) | Q9V4C8 | Cell cycle |
| 87 | Serrate RNA effector molecule | 3 | 34 (33) | Q9V9K7 | RNA-mediated gene silencing |
| 88 | Histone-lysine N-methyltransferase ash1 | 7 | 70 (33) | Q9VW15 | Chromatin-mediated maintenance of transcription |
| 89 | Exocyst complex component 6 | 5 | 67 (33) | Q9VDE6 | Exocytosis/cytoplasmic vesicle |
| 90 | Protein slender lobes | 8 | 66 (33) | Q8INM3 | Multicellular organismal development |
| 91 | Borealin | 5 | 64 (33) | Q9VLD6 | Cell cycle/cytokinesis |
| 92 | Beta-amyloid-like protein | 4 | 64 (33) | P14599 | Differentiation/Neurogenesis |
| 93 | Dystrophin, isoforms A/C/F/G/H | 7 | 62 (33) | **Q9VDW6** | Establishment of cell polarity |
| 94 | Protein three rows | 5 | 61 (33) | P42286 | Cell cycle |
| 95 | Serrate protein | 7 | 61 (33) | P18168 | Differentiation/Notch signaling pathway |
| 96 | Transcriptional regulator ATRX homolog | 6 | 58 (33) | Q9GQN5 | DNA damage |
| 97 | MAP kinase-activating death domain protein | 6 | 34 (33) | Q9VXY2 | Activation of MAPK activity |
| 98 | Neurogenic locus notch protein | 4 | 44 (32) | P07207 | Notch signaling pathway |
| 99 | Chromosomal serine/threonine-protein kinase JIL-1 | 5 | 43 (32) | Q9V3I5 | Transcription regulation |
| 100 | Integrin alpha-PS4 | 7 | 56 (33) | Q9V7A4 | Cell adhesion |
| 101 | Nuclear hormone receptor HR96 | 5 | 46 (32) | Q24143 | Regulation of transcription/DNA-dependent |
| 102 | Cadherin-related tumor suppressor | 6 | 54 (33) | P33450 | Cell adhesion |
| 103 | Exostosin-1 (Protein tout-velu) | 9 | 54 (33) | Q9V730 | Wnt signaling pathway |
| 104 | Ryanodine receptor 44F | 9 | 58 (32) | Q24498 | Calcium transport |
| 105 | Cell cycle regulator Mat89Bb | 4 | 52 (33) | B4QX59 | Cell cycle/regulation of mitotic cell cycle |
| 106 | Poly(A) RNA polymerase gld-2 homolog B | 6 | 52 (33) | Q9VYS4 | mRNA processing |
| 107 | Calpain-D | 5 | 52 (33) | P27398 | Nervous system development |
| 108 | Spastin | 5 | 51 (33) | B4G437 | Cell cycle |
| 109 | Serine protease nudel | 6 | 51 (33) | P98159 | Toll signaling pathway |
| 110 | FACT complex subunit spt16 | 5 | 51 (33) | Q8IRG6 | Transcription regulation |
| 111 | Polycomb group protein Psc | 10 | 63 (32) | P35820 | Chromatin remodeling |
| 112 | Protein lava lamp | 13 | 35 (33) | Q8MSS1 | Cellularization/Golgi apparatus |
| 113 | Protein cramped | 7 | 50 (33) | Q8MX88 | Regulation of transcription |
| 114 | Cyclin-T | 4 | 49 (33) | O96433 | Actin filament organization |
| 115 | Dual specificity tyrosine-phosphorylation-regulated kinase 2 | 5 | 49 (33) | Q9V3D5 | Olfactory behavior |
| 116 | Lysine-specific demethylase lid | 5 | 48 (33) | Q9VMJ7 | Histone H3-K4 demethylation |
| 117 | Enhancer of split m8 protein | 13 | 48 (33) | Q07291 | Cell differentiation |
| 118 | CCR4-NOT transcription complex | 4 | 48 (33) | Q9V3G6 | Phosphoprotein |
| 119 | Intraflagellar transport protein | 5 | 48 (33) | Q9W040 | Multicellular organismal development |
| 120 | Lateral signaling target protein | 3 | 47 (33) | B4IC49 | Negative regulator of epidermal growth factor receptor (EGFR) signaling |
| 121 | Myosin heavy chain 95F | 5 | 46 (33) | Q01989 | Actin cytoskeleton organization |
| 122 | Protein suppressor of variegation 3-7 | 2 | 33 (32) | P20193 | Dose-limiting factor in position-effect variegation |
| 123 | Elongation factor G | 5 | 46 (33) | B4KKD5 | Protein biosynthesis |
| 124 | Histone-lysine N-methyltransferase trithorax | 10 | 45 (33) | Q24742 | Chromatin modification |
| 125 | Proteasome-associated protein ECM29 | 7 | 70 (26) | Q9V677 | Protein catabolic process |
| 126 | Uncharacterized protein CG31531 | 5 | 44 (33) | Q0KIC3 | Not known |
| 127 | N-acetyltransferase eco | 6 | 55 (33) | Q9VS50 | Cell cycle |
| 128 | Glutamate [NMDA] receptor | 3 | 43 (33) | B4KD90 | Ion transport |
| 129 | Protein piwi | 7 | 52 (29) | Q9VKM1 | [RNA-mediated gene silencing](http://www.uniprot.org/keywords/KW-0943) |
| 130 | NADPH-cytochrome P450 reductase | 2 | 30 (29) | Q27597 | Oxidation reduction |
| 131 | Mitochondrial import inner membrane translocase | 12 | 42 (33) | Q9W4V8 | Protein transport |
| 132 | Caprin | 11 | 42 (33) | Q9I7D3 | Monolayer-surrounded lipid storage body |
| 133 | Putative N(4)-(beta-N-acetylglucosaminyl)-L-asparaginase GL17147 | 3 | 42 (33) | B4GGF2 | Protein deglycosylation |
| 134 | Homeobox protein extradenticle | 3 | 42 (33) | P40427 | Transcription |
| 135 | UPF0171 protein CG8783 | 4 | 41 (33) | Q9VUB4 | Protein binding |
| 136 | Apolipophorins | 19 | 32 (29) | **Q9V496** | [Lipid transport](http://www.uniprot.org/keywords/KW-0445)/[Wnt signaling pathway](http://www.uniprot.org/keywords/KW-0879) |
| 137 | Kinesin-like protein CG14535 | 6 | 42 (33) | Q9VLW2 | Microtubule-based movement |
| 138 | GDP-fucose transporter | 2 | 41 (33) | Q9VHT4 | Sugar transport/Golgi apparatus |
| 139 | Pituitary homeobox 1 | 12 | 41 (33) | O18400 | Transcription |
| 140 | Ankyrin repeat and KH domain-containing protein mask | 6 | 40 (33) | Q9VCA8 | Antimicrobial humoral response |
| 141 | Protein furry | 7 | 40 (33) | Q9VT28 | Transcription regulation/Phosphoprotein |
| 142 | Gamma-aminobutyric acid receptor alpha-like | 4 | 40 (33) | Q24352 | Ion transport (GABA, an inhibitory neurotransmitter) |
| 143 | Multidrug resistance protein homolog 49 | 4 | 39 (33) | Q00449 | Transport |
| 144 | ATP-dependent RNA helicase pitchoune | 4 | 39 (33) | Q9VD51 | Helicase/Hydrolase |
| 145 | Sodium- and chloride-dependent GABA transporter ine | 3 | 39 (33) | Q9VR07 | Differentiation/Neurogenesis |
| 146 | Tyrosine-protein kinase hopscotch | 5 | 39 (33) | Q24592 | Transcription regulation |
| 147 | Protein tramtrack, alpha isoform | 6 | 30 (29) | P42282 | [Transcription regulation](http://www.uniprot.org/keywords/KW-0805) |
| 148 | Potassium voltage-gated channel protein eag | 4 | 39 (33) | Q02280 | Differentiation/Ion transport/Neurogenesis |
| 149 | Open rectifier potassium channel protein 1 | 3 | 38 (33) | Q94526 | Ion transport |
| 150 | Protein eyes shut | 13 | 38 (33) | A0A1F4 | Rhabdomere development |
| 151 | Molybdenum cofactor sulfurase | 12 | 38 (33) | B4JXP7 | Molybdenum cofactor biosynthesis |
| 152 | GDP-mannose 4,6 dehydratase | 4 | 38 (33) | Q9VMW9 | GDP-L-fucose biosynthetic process/GDP-mannose metabolic process/intracellular |
| 153 | Succinate dehydrogenase [ubiquinone] flavoprotein | 11 | 43 (33) | Q94523 | Electron transport |
| 154 | Protein hook | 6 | 37 (33) | B4G831 | Endocytosis |
| 155 | Protein disabled | 5 | 37 (33) | P98081 | Differentiation/Neurogenesis |
| 156 | Putative fat-like cadherin-related tumor suppressor | 6 | 37 (33) | Q9VW71 | Cell adhesion |
| 157 | Zinc finger protein 2 | 4 | 37 (33) | P28167 | Imaginal disc-derived wing morphogenesis |
| 158 | Furin-like protease 1, isoform 1-CRR | 3 | 37 (33) | P30430 | Proteolysis/integral to membrane |
| 159 | E3 ubiquitin-protein ligase mind-bomb | 3 | 37 (33) | Q9VUX2 | Notch signaling pathway/Ubl conjugation pathway |
| 160 | Serendipity locus protein H-1 | 3 | 37 (33) | P15619 | Multicellular organismal development |
| 161 | Tripeptidyl-peptidase 2 | 2 | 36 (33) | Q9V6K1 | Protein homooligomerization |
| 162 | Formin-like protein CG32138 | 2 | 36 (33) | Q9VUC6 | Actin cytoskeleton organization |
| 163 | Protein SMG8 | 3 | 36 (33) | B4GH42 | Nonsense-mediated mRNA decay |
| 164 | Cytochrome P450 28d2 | 2 | 36 (33) | Q9VMT6 | Oxidation reduction |
| 165 | Transcription initiation factor TFIID subunit 8 | 13 | 36 (33) | Q9VWY6 | Regulation of transcription |
| 166 | Lethal(2) giant larvae protein | 5 | 36 (33) | P08111 | Cell cycle |
| 167 | RNA-directed DNA polymerase from transposon X-element | 3 | 35 (33) | Q9NBX4 | RNA-dependent DNA replication |
| 168 | Soluble guanylate cyclase 88E | 3 | 35 (33) | Q8INF0 | cGMP biosynthesis |
| 169 | PP2C-like domain-containing protein CG9801 | 6 | 52 (33) | Q0KIA2 | Not known |
| 170 | Putative dual specificity tyrosine-phosphorylation-regulated kinase 3 | 3 | 35 (33) | P83102 | Protein amino acid phosphorylation |
| 171 | Serine/threonine-protein kinase grp | 5 | 35 (29) | O61661 | Cell cycle |
| 172 | Poly [ADP-ribose] polymerase | 3 | 41 (33) | P35875 | Chromatin modification |
| 173 | Tyrosine-protein phosphatase Lar | 9 | 35 (33) | P16621 | Cell adhesion |
| 174 | Hormone receptor 4 | 2 | 34 (33) | Q9W539 | Transcription regulation |
| 175 | Eukaryotic translation initiation factor 3 | 4 | 34 (33) | B4GDX4 | Protein biosynthesis |
| 176 | Protein MCM10 | 6 | 34 (33) | Q9VIE6 | DNA replication |
| 177 | Myosin heavy chain, muscle | 3 | 34 (33) | P05661 | Epithelial cell migration |
| 178 | Disks large 1 tumor suppressor protein | 4 | 34 (33) | P31007 | Cell adhesion/Cell junction |
| 179 | Neither inactivation nor after potential protein C | 5 | 34 (33) | P10676 | Sensory transduction/Vision |
| 180 | Serine-enriched protein | 5 | 34 (33) | O61366 | Not known |
| 181 | Semaphorin-1A | 4 | 33 (29) | Q24322 | Differentiation/Neurogenesis (a role in growth cones guidance) |
| 182 | Raf homolog serine/threonine-protein kinase phl | 3 | 52 (33) | P11346 | Border follicle cell migration |
| 183 | Putative mitochondrial inner membrane protein | 3 | 34 (29) | P91928 | Integral to mitochondrial inner membrane |
| 184 | Protein purity of essence | 5 | 52 (33) | Q9VLT5 | Differentiation/Neurogenesis |
| 185 | Protein rigor mortis | 4 | 48 (33) | Q86BY9 | Transcription regulation |
| 186 | Protocadherin-like wing polarity protein stan | 5 | 45 (33) | Q9V5N8 | Cell adhesion/Cell membrane |
| 187 | Origin recognition complex subunit 1 | 6 | 46 (29) | O16810 | [DNA replication](http://www.uniprot.org/keywords/KW-0235) |
| 188 | Bipolar kinesin KRP-130 | 2 | 43 (33) | P46863 | Cell cycle/Microtubule |
| 189 | Protein BCL9 | 5 | 42 (33) | Q961D9 | Wnt signaling pathway |
| 190 | FACT complex subunit Ssrp1 | 4 | 42 (33) | Q05344 | DNA damage/DNA repair |
| 191 | Protein male-specific lethal-2 | 2 | 36 (33) | P50534 | Dosage compensation complex assembly |
| 192 | AP-2 complex subunit alpha | 3 | 31 (29) | P91926 | Endocytosis |
| 193 | H/ACA ribonucleoprotein complex non-core subunit NAF1 | 2 | 41 (33) | Q9VJ62 | Ribosome biogenesis/rRNA processing |
| 194 | Transcription elongation factor S-II | 2 | 33 (32) | P20232 | RNA elongation |
| 195 | Methionyl-tRNA synthetase | 2 | 39 (33) | Q9VFL5 | Protein biosynthesis |
| 196 | Phosphorylase b kinase regulatory | 3 | 38 (33) | Q9VLS1 | Carbohydrate/Glycogen metabolism |
| 197 | DNA repair protein RAD50 | 2 | 37 (33) | Q9W252 | Cell cycle/Chromosomal protein/ |
| 198 | DNA-binding protein modulo | 3 | 36 (33) | P13469 | Cell proliferation |
| 199 | Putative gustatory receptor 98a | 4 | 36 (33) | Q9VB30 | G-protein coupled receptor protein signaling pathway |
| 200 | Cadherin-86C | 3 | 36 (33) | Q9VGW1 | Cell adhesion |
| 201 | Kinesin-like protein GA13060 | 3 | 35 (33) | Q29MB2 | Microtubule-based movement |
| 202 | ATP synthase subunit beta | 3 | 35 (33) | Q05825 | ATP synthesis coupled proton transport |
| 203 | Putative tyrosine-protein kinase Wsck | 4 | 35 (33) | P83097 | Protein amino acid phosphorylation |
| 204 | Bifunctional heparan sulfate N-deacetylase/N-sulfotransferase | 2 | 35 (33) | Q9V3L1 | Wnt signaling pathway |
| 205 | Transmembrane GTPase Marf | 3 | 34 (33) | Q7YU24 | Mitochondrial fusion |
| 206 | Chromodomain-helicase-DNA-binding protein 1 | 2 | 34 (32) | Q7KU24 | Chromatin assembly or disassembly |
| 207 | cAMP-dependent protein kinase catalytic | 2 | 34 (33) | P12370 | Anterior/posterior pattern formation, imaginal disc/plasma membrane |
| 208 | Hsp90 co-chaperone Cdc37 | 4 | 30 (29) | Q24276 | Cell cycle |
| 209 | Yemanuclein-alpha | 2 | 34 (32) | P25992 | Egg organization/transcriptional regulator |
| 210 | Claspin | 3 | 45 (32) | Q8IRB5 | Phosphoprotein |
| 211 | Diacylglycerol kinase eta | 5 | 44 (32) | B4JHJ7 | Activation of protein kinase C activity by G-protein coupled receptor protein signaling pathway |
| 212 | Nucleosome-remodeling factor subunit NURF301 | 4 | 43 (32) | Q9W0T1 | Dendrite morphogenesis |
| 213 | Another transcription unit protein | 5 | 42 (32) | Q94546 | Phosphoprotein |
| 214 | Protein CLP1 | 2 | 40 (32) | B4HQJ2 | mRNA processing |
| 215 | F-box/WD repeat-containing protein 7 | 3 | 39 (32) | Q9VZF4 | Cell cycle/Ubl conjugation pathway |
| 216 | Actin-binding protein anillin | 2 | 39 (32) | Q9V4P1 | Cell cycle |
| 217 | Protein single-minded | 2 | 37 (32) | P05709 | Differentiation/Neurogenesis |
| 218 | Uncharacterized protein CG4951 | 2 | 37 (32) | A1A708 | Phosphoprotein |
| 219 | Puff-specific protein Bx42 | 2 | 37 (32) | P39736 | Embryonic development via the syncytial blastoderm/eye-antennal disc development |
| 220 | L-asparaginase CG7860 | 2 | 36 (32) | Q9VXT7 | Asparagine catabolic process via L-aspartate |
| 221 | Broad-complex core protein | 4 | 35 (32) | Q24206 | Autophagy |
| 222 | Serine protease HTRA2 | 3 | 35 (32) | B4K835 | Apoptosis |
| 223 | Protein scabrous | 2 | 34 (32) | P21520 | Notch signaling pathway/extracellular space |
| 224 | Fragile X mental retardation syndrome-related protein 1 | 4 | 33 (29) | Q9NFU0 | RNA-mediated gene silencing |
| 225 | SH3 domain-binding protein 5 | 3 | 45 (29) | Q9V785 | Anterior/posterior pattern formation |
| 226 | Cytochrome P450 4aa1 | 2 | 32 (29) | Q9V7G5 | Oxidation reduction (metabolism of insect hormones and breakdown of synthetic insecticides) |
| 227 | rRNA-processing protein EBP2 | 4 | 30 (29) | Q9V9Z9 | Ribosome biogenesis |
| 228 | DNA replication licensing factor MCM4 | 8 | 30 (29) | Q26454 | Mitotic DNA replication |
| 229 | Myosin-VIIa | 3 | 40 (33) | Q29P71 | Actin filament-based movement |
| 230 | Bicaudal D-related protein | 5 | 87 (33) | Q8SWR2 | Belongs to the [BICDR family](http://www.uniprot.org/uniprot/?query=family:"BICDR+family") |
| 231 | Flotillin-2 | 4 | 41 (32) | O61492 | Cell adhesion |
| 232 | Transcription initiation factor TFIID subunit 4 | 2 | 36 (32) | P47825 | Transcription regulation |
| 233 | Multidrug resistance-associated protein lethal(2)03659 | 7 | 36 (29) | P91660 | Multicellular organismal development |
| 234 | DNA repair protein complementing XP-C cells | 3 | 34 (32) | Q24595 | DNA damage/DNA repair |
| 235 | 26S protease regulatory subunit 4 | 6 | 32 (29) | P48601 | Cell proliferation (ATP-dependent degradation of ubiquitinated proteins) |
| 236 | RuvB-like helicase 2 | 2 | 35 (33) | Q9V3K3 | Wnt receptor signaling pathway |
| 237 | Serine/threonine-protein kinase ATR | 2 | 34 (33) | Q9VXG8 | DNA damage checkpoint/chromosome |
| 238 | Transcription initiation factor TFIID subunit 1 | 4 | 57 (33) | P51123 | Cell cycle |
| 239 | Regulator of telomere elongation helicase 1 | 4 | 46 (33) | B3MSG8 | DNA damage/DNA repair/Nucleus |
| 240 | Cytochrome P450 309a1 | 19 | 37 (29) | Q9VQD2 | Oxidation reduction |
| 241 | Protein Shroom | 6 | 39 (33) | A1Z9P3 | Cell migration |
| 242 | Polypeptide N-acetyl galactosaminyl transferase 35A | 3 | 38 (33) | Q8MVS5 | Oligosaccharide biosynthetic process/Golgi stack |
| 243 | Queuine tRNA-ribosyltransferase | 2 | 38 (33) | B4PEV9 | Queuosine biosynthesis/tRNA processing |
| 244 | Dynein heavy chain | 4 | 36 (33) | P37276 | RNA transport |
| 245 | Defective chorion-1 protein, FC125 | 2 | 34 (32) | P18169 | Eggshell chorion assembly |
| 246 | Cytochrome P450 4d14 | 3 | 34 (33) | O46051 | Oxidation reduction |
| 247 | Protein strawberry notch | 5 | 59 (33) | A8JUV0 | Notch signaling pathway |
| 248 | Integrin alpha-PS2 | 7 | 37 (29) | P12080 | Cell adhesion |
| 249 | CWF19-like protein 2 | 2 | 40 (33) | Q9VXT5 | Phosphoprotein |
| 250 | Eukaryotic translation initiation factor 3 subunit C | 2 | 40 (33) | B4MRZ8 | Protein biosynthesis |
| 251 | Polycomb protein Scm | 3 | 37 (33) | Q9VHA0 | Axonogenesis |
| 252 | Rho GTPase-activating protein CG5521 | 2 | 37 (33) | Q9VB98 | Regulation of small GTPase mediated signal transduction |
| 253 | Neural/ectodermal development factor IMP-L2 | 2 | 36 (33) | Q09024 | Cell adhesion/extracellular space |
| 254 | Maternal protein exuperantia-1 | 2 | 35 (33) | Q24618 | Multicellular organismal development |
| 255 | Protein vav | 3 | 47 (33) | Q9NHV9 | Actin filament organization |
| 256 | JNK-interacting protein 3 | 3 | 43 (32) | Q9GQF1 | Axon cargo transport/regulation of JNK cascade |
| 257 | Voltage-dependent calcium channel type D subunit alpha-1 | 5 | 32 (29) | Q24270 | Calcium transport |
| 258 | Receptor-mediated endocytosis protein 6 | 2 | 37 (33) | Q9VZ08 | Endocytosis |
| 259 | Sodium channel protein 60E | 2 | 37 (33) | Q9W0Y8 | Olfactory behavior/voltage-gated sodium channel complex |
| 260 | Longitudinals lacking protein, isoform G | 2 | 36 (33) | P42283 | Differentiation/Neurogenesis |
| 261 | V-type proton ATPase catalytic subunit A | 2 | 34 (33) | P48602 | ATP synthesis coupled proton transport |
| 262 | Ribosomal RNA processing protein | 6 | 38(29) | Q9VJZ7 | rRNA processing |
| 263 | Protein bowel | 3 | 42 (32) | Q9VQU9 | Transcription regulation |
| 264 | E3 ubiquitin-protein ligase hyd | 6 | 50 (32) | P51592 | Ubl conjugation pathway |
| 265 | Eukaryotic translation initiation factor 3 subunit I | 2 | 36 (32) | B4LUA5 | Protein biosynthesis |
| 266 | Integrin alpha-PS1 | 4 | 36 (32) | Q24247 | Cell adhesion |
| 267 | Serine/threonine-protein kinase polo | 3 | 30 (29) | P52304 | Cytokinesis |
| 268 | Protein 4.1 | 5 | 57 (32) | Q9V8R9 | Chitin-based embryonic cuticle biosynthetic process |
| 269 | 205 kDa microtubule-associated protein | 3 | 38 (32) | P23226 | Phosphoprotein |
| 270 | Glucose-dehydrogenase | 12 | 45 (29) | P18172 | Alcohol metabolic process |
| 271 | SpoIIM-like stage II sporulation protein M related | 4 | 40 (29) | Q9V182 | Not known |
| 272 | Protein bicaudal D | 4 | 37 (29) | P16568 | Germarium-derived oocyte fate determination |
| 273 | Protein O-mannosyl-transferase 2 | 8 | 36 (29) | Q9W5D4 | Lipid glycosylation ( in association with each other to generate and maintain normal muscle development |
| 274 | Polypeptide N-acetylgalactosaminyl- transferase 3 | 4 | 35 (29) | Q9Y117 | Protein glycosylation |
| 275 | Putative neural-cadherin 2 | 4 | 33 (29) | Q9VJB6 | Calcium dependent cell adhesion proteins |
| 276 | Frizzled-2 | 3 | 32 (29) | Q9VVX3 | Wnt signaling pathway |
| 277 | Guanine nucleotide-releasing factor 2 | 7 | 32 (29) | **O77086** | Ras protein signal transduction |
| 278 | Calpain-C | 4 | 31 (29) | Q9VXH6 | Not known (proteolysis) |
| 279 | E3 ubiquitin-protein ligase Smurf1 | 7 | 30 (29) | Q9V853 | Ubl conjugation pathway |
| 280 | 120.7 kDa protein in NOF-FB transposable element | 6 | 30 (29) | P16320 | Transposition of NOF-FB and other FB elements |
| 281 | Spindle assembly abnormal protein 6 | 3 | 30 (29) | Q9VAC8 | Cell cycle |
| 282 | Laminin subunit alpha | 7 | 45 (30) | Q00174 | Cell adhesion (mediation of the attachment) |
| 283 | DNA mismatch repair protein Msh6 | 10 | 60 (29) | Q9VUM0 | Post-replicative DNA-mismatch repair |
| 284 | Polycomb protein Asx | 4 | 36 (30) | Q9V727 | Transcription regulation |
| 285 | Protein abnormal spindle | 7 | 35 (30) | Q9VC45 | Required to maintain the structure of the centrosomal microtubule-organizing center (MTOC) during mitosis |
| 286 | E3 ubiquitin-protein ligase Bre1 | 4 | 43 (29) | Q9VRP9 | Notch signaling pathway/Ubl conjugation pathway |
| 287 | Eukaryotic translation initiation factor 2-alpha kinase | 2 | 30 (29) | Q9NIV1 | Stress response/Translation regulation/Unfolded protein response |
| 288 | Protein sevenless | 4 | 35 (30) | P20806 | Sensory transduction |
| 289 | G1/S-specific cyclin-E | 4 | 44 (29) | **P54733** | Essential for the control of the cell cycle at the G1/S (start) transition |
| 290 | Stress-activated map kinase-interacting protein 1 | 7 | 36 (29) | Q9V719 | Apoptosis |
| 291 | Glutaminyl-tRNA synthetase | 5 | 33 (30) | Q9Y105 | Protein biosynthesis |
| 292 | Modifier of mdg4 | 5 | 32 (30) | **Q86B87** | Apoptosis |
| 293 | Protein ariadne-1 | 15 | 42 (30) | Q94981 | Ubl conjugation pathway |
| 294 | FK506-binding protein 59 | 2 | 30 (29) | Q9VL78 | Sensory transduction (phototransduction; inhibits or prevents Ca^2+^ induced stimulation of the trpl ion channel) |
| 295 | Hepatocyte growth factor-regulated tyrosine kinase substrate | 3 | 40 (29) | Q960X8 | Border follicle cell migration |
| 296 | Restin | 8 | 37 (29) | **Q9VJE5** | Cellularization |
| 297 | Protein phosphatase PHLPP-like protein | 2 | 30 (29) | Q9VJ07 | Apoptosis |
| 298 | Laminin subunit gamma-1 | 5 | 38 (29) | **P15215** | Cell adhesion |
| 299 | Protein Wnt-5 | 3 | 37 (29) | P28466 | Wnt signaling pathway |
| 300 | Attacin-C | 2 | 30 (29) | **Q95NH6** | Innate immunity |
| 301 | Dosage compensation regulator | 28 | 40 (29) | **P24785** | Axon extension |
| 302 | ADP, ATP carrier protein | 22 | 30 (29) | Q26365 | Transport |
| 303 | Putative cytoplasmic aminopeptidase | 5 | 38 (29) | **Q9V3D8** | Proteolysis |
| 304 | DNA topoisomerase 3-alpha | 7 | 37 (29) | Q9NG98 | DNA topological change |
| 305 | Putative ribosomal RNA methyltransferase CG7009 | 18 | 30 (29) | Q9VDD9 | rRNA processing |
| 306 | Nuclear hormone receptor FTZ-F1 | 7 | 34 (29) | P33244 | Cofactor to fushi tarazu (ftz) |
| 307 | Putative 1-phosphatidylinositol-3-phosphate 5-kinase | 4 | 30 (29) | O96838 | Cellular protein metabolic process |
| 308 | Heat shock 70 kDa protein Ba | 7 | 41 (29) | **Q8INI8** | Stress response |
| 309 | CG15580, isoform A | 3 | 48 (36) | Q9VNK8 | Protein binding |
| 310 | CG18255-PA | 6 | 47 (37) | Q8MLD9 | Not known |
| 311 | DOMON domain-containing protein CG14681 | 6 | 32 (28) | Q9VGY6 | Catecholamine metabolic process/Histidine catabolic process |
| 312 | CG13917 | 3 | 42 (36) | Q9W0D3 | Protein binding |
| 313 | LD27161p | 8 | 34 (40) | Q8MRI5 | Ovarian follicle cell development |
| 314 | Surf6-PA | 8 | 40 (36) | Q8I151 | Not known |
| 315 | Lethal (1) G0060, isoform A | 16 | 46 (36) | Q9W485 | Not known |
| 316 | Papilin | 13 | 80 (26) | Q868Z9 | Extracellular matrix organization |
| 317 | CG7516 | 6 | 50 (36) | Q9V3P2 | Protein binding |
| 318 | Srp72 | 5 | 54 (36) | Q9VDK7 | SRP-dependent cotranslational protein targeting to membrane |
| 319 | BcDNA.LD27873 | 10 | 48 (36) | Q9V3H9 | Phagocytosis, engulfment |
| 320 | GH14426p | 28 | 50 (36) | Q6NNA4 | Not known |
| 321 | LP12301p | 6 | 46 (36) | Q960H1 | Oxidation reduction |
| 322 | Aromatic-L-amino-acid decarboxylase | 7 | 72 (36) | Q7Z0J7 | Carboxylic acid metabolic process |
| 323 | CG14864, isoform A | 9 | 80 (37) | Q9VFA3 | Not known |
| 324 | Mekk1, isoform B | 8 | 54 (37) | Q8MSQ4 | MAPKKK cascade |
| 325 | CG12187 | 8 | 40 (37) | Q9VZY3 | Protein binding |
| 326 | SD02424p | 10 | 54 (37) | Q8MSS0 | Binding |
| 327 | CG5792, isoform C | 7 | 44 (36) | Q9VK58 | Not known |
| 328 | CG10631 | 6 | 42 (36) | Q9VIS5 | Nucleic acid binding |
| 329 | CG11008-PA | 7 | 40 (36) | Q8I174 | DNA binding |
| 330 | LD01527p | 12 | 40 (36) | Q9VJ35 | Mitotic spindle elongation |
| 331 | CG7971, isoform A | 10 | 39 (36) | Q7YZ99 | Nuclear mRNA splicing, via spliceosome |
| 332 | RE22456p | 5 | 53 (36) | Q8SXT9 | Contractile ring contraction involved in cell cycle cytokinesis |
| 333 | AT29074p | 8 | 40 (26) | Q6NNX7 | Type-B carboxylesterase/lipase family |
| 334 | CG9313 | 3 | 42 (36) | Q7KVQ2 | ATPase activity, uncoupled |
| 335 | LD41783p | 3 | 38 (36) | Q8SWR4 | Not known |
| 336 | Inositol 1,4,5-trisphosphate receptor (InsP_3_R) | 5 | 43 (33) | P29993 | Second messenger that mediates the release of intracellular calcium/Calcium transport |
| 337 | Probable serine/threonine-protein kinase zyg-1 | 20 | 60 (26) | Q621J7 | Cell cycle ***Caenorhabditis briggsa*e** |
| 338 | Dynein beta chain, ciliary | 19 | 36 (25) | P23098 | Cilium biogenesis/degradation ***Tripneustes gratilla* (Hawaian sea urchin)** |
| 339 | Cytoplasmic polyadenylation element-binding protein 3 | 5 | 35 (26) | Q6E3D5 | RNA binding ***Caenorhabditis briggsae*** |
| 340 | Dynein beta chain, ciliary | 22 | 57 (26) | P39057 | Cilium biogenesis/degradation ***Anthocidaris crassispina* (Sea urchin)** |
| 341 | Sodium channel protein 1 brain | 6 | 30 (26) | Q05973 | Sodium transport ***Loligo bleekeri* (Bleeker's squid) (Doryteuthis bleekeri)** |
| 342 | Hemocytin | 7 | 30 (26) | P98092 | Cell adhesion ***Bombyx mori* (silk moth)** |
| 343 | Nuclear hormone receptor E75 | 3 | 30 (26) | O77245 | Regulation of transcription, DNA-dependent ***Metapenaeus ensis* (Greasyback shrimp) (Sand shrimp)** |
| 344 | Enolase | 4 | 29 (26) | Q27655 | Glycolysis ***Fasciola hepatica* (Liver fluke)** |
| 345 | DNA topoisomerase 2 | 6 | 28 (26) | O16140 | DNA topological change ***Bombyx mori* (silk moth)** |
| 346 | Vitellogenin | 7 | 36 (26) | Q05808 | Lipid transport ***Anthonomus grandis* (Boll weevil) (Anthonomus thurberiae)** |
| 347 | U17-ctenitoxin-Pn1a | 2 | 27 (26) | P83996 | Pathogenesis **Phoneutria nigriventer *Brazilian armed spider* (Ctenus nigriventer)** |
| 348 | 60S ribosomal protein L13a | 12 | 31 (26) | Q8MUR4 | Ribonucleoprotein **Choristoneura parallela (Spotted fireworm moth)** |
| 349 | Muscle calcium channel subunit alpha-1 | 7 | 31 (26) | Q25452 | Calcium transport **Musca domestica (House fly)** |
| 350 | Non-neuronal cytoplasmic intermediate filament protein | 5 | 27 (25) | P22488 | Structural molecule activity ***Helix aspersa* (Brown garden snail)** |
| 351 | Venom allergen 5 | 3 | 30 (26) | P81657 | Disulfide bond **Vespa mandarinia (Hornet)** |
| 352 | Nucleolar complex protein 3 homolog | 13 | 28 (26) | Q61LN7 | CBF/MAK21 family ***Caenorhabditis briggsae*** |
| 353 | 40S ribosomal protein S3a | 4 | 38 (26) | P49395 | Translation ***Aplysia californica* (California sea hare)** |
| 354 | 60S ribosomal protein L13a | 11 | 27 (26) | Q962U0 | Ribonucleoprotein ***Spodoptera frugiperda* (Fall armyworm)** |
| 355 | Insulin-like receptor | 4 | 28 (25) | Q93105 | Insulin receptor signaling pathway ***Aedes aegypti* (Yellowfever mosquito) (Culex aegypti)** |
| 356 | Aminopeptidase N | 5 | 34 (25) | Q10737 | Proteolysis ***Haemonchus contortus* (Barber pole worm)** |
| 357 | Squidulin | 3 | 27 (26) | P14533 | Calcium ion binding ***Loligo pealeii* (Longfin inshore squid) (Loligo pallida)** |
| 358 | Protein ultraspiracle homolog | 4 | 40 (25) | P54779 | Regulation of transcription, DNA-dependent ***Manduca sexta* (Tobacco hawkmoth) (Tobacco hornworm)** |
| 359 | Major antigen | 11 | 37 (25) | P21249 | Myofibrillar protein ***Onchocerca volvulus*** |
| 360 | Resact receptor | 4 | 34 (25) | P11528 | cGMP biosynthesis ***Arbacia punctulata* (Punctuate sea urchin)** |
| 361 | Alpha-scruin | 6 | 30 (25) | Q25390 | Actin bundling protein found in the acrosomal sperm process ***Limulus polyphemus* (Atlantic horseshoe crab)** |
| 362 | Severin | 26 | 34 (26) | Q24800 | Actin filament capping ***Echinococcus granulosus*** |
| 363 | Vitellogenin-A1 | 5 | 28 (25) | Q16927 | Lipid transport ***Aedes aegypti* (Yellowfever mosquito) (Culex aegypti)** |
| 364 | Histone H2B.2, embryonic | 3 | 28 (25) | P02288 | Nucleosome assembly ***Psammechinus miliaris* (Sand sea urchin)** |
| 365 | Asparaginyl-tRNA synthetase, cytoplasmic | 5 | 31 (25) | P10723 | Protein biosynthesis ***Brugia malayi* (Filarial nematode worm)** |
| 366 | Cadmium-metallothionein | 7 | 30 (25) | P81695 | High content of cysteine residues that bind various heavy metals ***Eisenia foetida* (Common brandling worm)** |
| 367 | Puff II/9-2 protein | 3 | 26 (25) | P22312 | Glycoprotein ***Sciara coprophila* (Fungus gnat)** |
| 368 | Arylphorin subunit A4 | 5 | 26 (25) | P28513 | Transport ***Calliphora vicina* (Blue blowfly) (Calliphora erythrocephala)** |
| 369 | Contryphan-Sm | 2 | 31 (24) | P58787 | Pathogenesis ***Conus stercusmuscarum* (Fly-specked cone)** |
| 370 | 40S ribosomal protein S23 | 2 | 28 (26) | Q9GRJ3 | Ribonucleoprotein ***Lumbricus rubellus* (Humus earthworm)** |
| 371 | GTP-binding nuclear protein Ran | 8 | 41 (25) | P38542 | Protein transport ***Brugia malayi* (Filarial nematode worm)** |
| 372 | 77 kDa echinoderm microtubule-associated protein | 6 | 28 (25) | Q26613 | Proteolysis ***Strongylocentrotus purpuratus* (Purple sea urchin)** |
| 373 | Actin, muscle-type A2 | 4 | 27 (26) | P07837 | Actin family ***Bombyx mori* (silk moth)** |
| 374 | Nitric oxide synthase, salivary gland | 3 | 31 (25) | Q26240 | Nitric oxide biosynthetic process ***Rhodnius prolixus* (Triatomid bug)** |
| 375 | Vitellogenin-2 | 11 | 30 (25) | Q9BPS0 | Lipid transport ***Periplaneta americana* (American cockroach) (Blatta americana)** |
| 376 | S-crystallin 3 | 4 | 28 (25) | Q25626 | Structural components of squids and octopi eye lens ***Octopus vulgaris*** |
| 377 | Major yolk protein | 5 | 27 (25) | P19615 | Cellular iron ion homeostasis ***Strongylocentrotus purpuratus* (Purple sea urchin)** |
| 378 | V-type proton ATPase subunit H | 3 | 26 (25) | Q9U5N0 | Hydrogen ion transport/Ion transport ***Manduca sexta* (Tobacco hawkmoth) (Tobacco hornworm)** |
| 379 | Glutamate carboxypeptidase 2 homolog | 7 | 27 (25) | Q5WN23 | Proteolysis ***Caenorhabditis briggsae*** |
| 380 | Spindle-defective protein 2 | 4 | 40 (25) | Q61DP2 | Cell cycle ***Caenorhabditis briggsae*** |
| 381 | Calcium-dependent protein kinase C | 5 | 33 (25) | Q16974 | Protein amino acid phosphorylation ***Aplysia californica* (California sea hare)** |
| 382 | DNA (cytosine-5)-methyltransferase PliMCI | 5 | 30 (25) | Q27746 | Methylates CpG residues ***Paracentrotus lividus* (Common sea urchin)** |
| 383 | 40S ribosomal protein S24 | 25 | 29 (25) | Q962Q6 | Ribonucleoprotein ***Spodoptera frugiperda* (Fall armyworm)** |
| 384 | Glutathione S-transferase class-mu 28 kDa isozyme | 5 | 29 (25) | P30114 | Transferase ***Schistosoma haematobium* (Blood fluke)** |
| 385 | Metallothionein | 20 | 27 (25) | P55953 | High content of cysteine residues that bind various heavy metals ***Sterechinus neumayeri* (Antarctic sea urchin)** |
| 386 | Orphan steroid hormone receptor 2 | 6 | 51 (26) | Q26622 | Transcription regulation ***Strongylocentrotus purpuratus* (Purple sea urchin)** |
| 387 | Protein SpAN | 4 | 30 (26) | P98068 | Multicellular organismal development ***Strongylocentrotus purpuratus* (Purple sea urchin)** |
| 388 | Metallothionein-A | 21 | 27 (26) | Q26497 | High content of cysteine residues that bind various heavy metals ***Sphaerechinus granularis* (Purple sea urchin)** |
| 389 | Bifunctional arginine demethylase and lysyl-hydroxylase psr-1 | 3 | 26 (25) | Q623U2 | Transcription regulation ***Caenorhabditis briggsae*** |
| 390 | Myosin heavy chain, striated muscle | 12 | 30 (25) | P24733 | Motor protein ***Aequipecten irradians* (Bay scallop) (Argopecten irradians)** |
| 391 | Luciferin 4-monooxygenase | 3 | 36 (26) | Q01158 | Luminescence ***Luciola lateralis* (Firefly)** |
| 392 | Heat shock protein 70 | 3 | 32 (26) | P91902 | Stress response ***Ceratitis capitata* (Mediterranean fruit fly) (Tephritis capitata)** |
| 393 | Sex-determining transformer protein 2 | 7 | 31 (26) | Q17307 | Sexual differentiation ***Caenorhabditis briggsae*** |
| 394 | Tropomyosin | 6 | 31 (25) | Q8T380 | Central role in the calcium dependent regulation of muscle contraction ***Lepisma saccharina* (silverfish)** |
| 395 | Molt-inhibiting hormone-like | 2 | 27 (26) | P55322 | Neuropeptide signaling pathway ***Litopenaeus vannamei* (Whiteleg shrimp) (Penaeus vannamei)** |
| 396 | Vitellogenin | 2 | 31 (25) | Q27309 | Lipid transport ***Bombyx mori* (silk moth)** |
| 397 | Kinesin light chain | 5 | 28 (25) | Q05090 | Organelle transport ***Strongylocentrotus purpuratus* (Purple sea urchin)** |
| 398 | Dihydropyrimidinase 1 | 5 | 28 (25) | Q60Q85 | Hydrolase ***Caenorhabditis briggsae*** |
| 399 | Egg-lysin | 3 | 26 (25) | Q01383 | Fertilization ***Haliotis sorenseni* (White abalone)** |
| 400 | GMP reductase | 3 | 36 (25) | P27442 | Nucleotide metabolic process ***Ascaris suum* (Pig roundworm) (Ascaris lumbricoides)** |
