# Supplementary material for: Diet-Induced Over-Expression of Flightless-I Protein and Its Relation to Flightlessness in Mediterranean Fruit Fly, Ceratitis capitata
Source: PLoS One. 2013 Dec 3;8(12):e81099. doi: 10.1371/journal.pone.0081099 (PMC3849048; doi:10.1371/journal.pone.0081099)
Supplement: Table S2 — A list of 406 proteins detected in pupae B whose larvae were reared in the liquid diet. (DOC) [file pone.0081099.s002.doc]

**Supporting Information (SI)**

**Diet-induced over-expression of flightless-I protein and its relation to flightlessness in Mediterranean fruit fly, *Ceratitis capitata***

Il Kyu Cho^1^, Chiou Ling Chang^2^ and Qing X. Li^1^*

^1^ Department of Molecular Biosciences and Bioengineering, University of Hawaii, Honolulu, Hawaii, USA.

^2^ U.S. Pacific Basin Agricultural Research Center, Hilo, Hawaii, USA.

**Table S2**. **A list of 406 proteins detected in pupae B whose larvae were reared in the liquid diet.** The LC-MS/MS data were matched with *Drosophila melanogaster* database via MASCOT for the sequence alignments.

| No. | Protein names | No. of matched peptides | Mascot scores (p=0.05) | Accession numbers | Biological functions |
| --- | --- | --- | --- | --- | --- |
| 1 | DNA topoisomerase 3-alpha | 3 | 41 (30) | Q9NG98 | DNA topological change/chromosome |
| 2 | 39 kDa FK506-binding nuclear protein | 3 | 44 (30) | P54397 | Protein folding |
| 3 | Ribosomal L1 domain-containing protein CG13096 | 8 | 47 (30) | Q9VLK2 | RNA processing |
| 4 | Methyltransferase-like protein 13 | 6 | 40 (30) | Q29LW1 | Metabolic process |
| 5 | Diacylglycerol kinase eta | 10 | 45 (30) | B3LXF2 | Protein kinase C activity by G-protein coupled receptor protein Signaling pathway |
| 6 | Ras-related protein Ral-a | 7 | 38 (30) | P48555 | Negative regulation of JNK cascade/small GTPase mediated Signal transduction |
| 7 | Protein wings apart-like | 7 | 52 (30) | Q9W517 | Chromosome partition |
| 8 | Serine/threonine-protein kinase Genghis Khan | 14 | 57 (30) | **Q9W1B0** | Downstream effector for the regulation of actin polymerization by Cdc42 |
| 9 | Ribosome biogenesis protein WDR12 | 10 | 36 (30) | B4KKN1 | Ribosome biogenesis/rRNA processing |
| 10 | Protein bicaudal D | 9 | 36 (30) | P16568 | Differentiation |
| 11 | Titin | 12 | 91 (30) | Q9I7U4 | Cell cycle/Cell division/Mitosis |
| 12 | Intraflagellar transport protein osm-1 | 3 | 35 (30) | Q9W040 | Maintenance and formation of cilia |
| 13 | Polypeptide N-acetyl galactosaminyl transferase 35A | 3 | 35 (30) | Q8MVS5 | Essential glycotransferase |
| 14 | Ecdysone-induced protein 75B, isoform B | 3 | 35 (30) | P13055 | Regulation of ecdysone-triggered gene hierarchies |
| 15 | Kinesin light chain | 2 | 34 (30) | P46824 | Microtubule-associated force-producing protein that may play a role in organelle transport |
| 16 | Capicua | 3 | 37 (30) | Q9U1H0 | Transcription regulation |
| 17 | Nucleolar complex protein 3 | 13 | 58 (31) | Q9VI82 | Binding |
| 18 | WD repeat-containing protein on Y chromosome | 8 | 48 (31) | B7FF08 | Repeat |
| 19 | DNA topoisomerase 1 | 5 | 41 (31) | P30189 | DNA topological change |
| 20 | Mediator of RNA polymerase II transcription subunit 1 | 6 | 53 (31) | Q9VP05 | Regulation of transcription from RNA polymerase II promoter |
| 21 | Protein abnormal spindle | 8 | 53 (29) | Q9VC45 | Cell cycle |
| 22 | Protein zer-1 | 3 | 37 (31) | Q9W0E8 | Ubl conjugation pathway/Cul2-RING ubiquitin ligase complex |
| 23 | Protein KIAA0664 homolog | 2 | 36 (31) | B4MY63 | KIAA0664/TIF31 family |
| 24 | Protein piwi | 2 | 36 (31) | Q9VKM1 | RNA-mediated gene silencing |
| 25 | ATP-dependent RNA helicase CG8611 | 2 | 34 (31) | Q86B47 | ATP-dependent RNA helicase/hydrolase |
| 26 | Multidrug resistance-associated protein Lethal (2) 03659 | 7 | 36 (26) | P91660 | Multicellular organismal development |
| 27 | Synapsin | 6 | 31 (31) | Q24546 | Behavior/cell junction |
| 28 | 205 kDa microtubule-associated protein | 2 | 31 (31) | P23226 | Regulation of microtubule assembly and interaction |
| 29 | PHD finger protein rhinoceros | 13 | 72 (26) | Q7YZH1 | Negative regulator of the EGFR/Ras/MAPK signaling pathway during eye development |
| 30 | Protein dopey-1 | 7 | 48 (31) | Q292H2 | Protein transport |
| 31 | Origin recognition complex subunit 1 | 2 | 45 (31) | O16810 | DNA replication |
| 32 | Protein still life, isoforms C/siF type 2 | 6 | 45 (30) | P91620 | Intracellular signaling cascade/regulation of Rho protein signal transduction/cell junction |
| 33 | Fasciclin-1 | 3 | 42 (31) | P10674 | Cell adhesion |
| 34 | Chromodomain-helicase-DNA-binding protein 1 | 3 | 41 (31) | Q7KU24 | Transcription regulation |
| 35 | Histone-lysine N-methyltransferase CG171 | 6 | 47 (31) | Q9VYD1 | Specific tags for epigenetic transcriptional activation or repression |
| 36 | Nucleolar protein 6 | 3 | 36 (31) | B4NIM9 | RNA binding |
| 37 | WASH complex subunit FAM21 homolog | 2 | 36 (31) | A1ZBW7 | Phosphoprotein |
| 38 | Neither inactivation nor after potential protein C | 7 | 35 (31) | P10676 | Sensory transduction/photoreceptor cell (combines putative serine/threonine-protein kinase and myosin activities) |
| 39 | Protein bric-a-brac 1 | 19 | 35 (31) | Q9W0K7 | Transcription regulation |
| 40 | RNA-binding protein CG14230 | 5 | 41 (31) | Q9VWD4 | RNA binding |
| 41 | Nuclear RNA export factor 2 | 3 | 41 (31) | Q9VV73 | Export of mRNA from the nucleus to the cytoplasm |
| 42 | Pescadillo | 4 | 37 (31) | B3N8H0 | Ribosome biogenesis/rRNA processing |
| 43 | Protein unc-80 | 5 | 37 (31) | Q9VB11 | Cation homeostasis |
| 44 | Regulator of telomere elongation helicase 1 | 5 | 35 (31) | B4I0K4 | DNA damage/DNA repair |
| 45 | Axoneme-associated protein mst101(1) | 3 | 34 (31) | Q08695 | Sperm axoneme assembly |
| 46 | DNA-binding protein D-ETS-6 | 3 | 32 (31) | P29776 | dendrite morphogenesis/nucleus |
| 47 | cAMP-specific 3~, 5~-cyclic phosphodiesterase | 2 | 32 (31) | P12252 | A key regulator of many important physiological processes |
| 49 | Polypeptide N-acetyl galactosaminyl transferase 1 | 21 | 31 (31) | Q6WV20 | Oligosaccharide biosynthetic process |
| 50 | Epidermal growth factor receptor | 4 | 38 (31) | P04412 | Muscle cell fate specification |
| 51 | Protein spire | 3 | 37 (31) | Q9U1K1 | Transport |
| 52 | Protein suppressor of white apricot | 3 | 36 (31) | **P12297** | Transcription regulation |
| 53 | DM7 family protein CG15332 | 5 | 34 (31) | Q9W3M2 | DM7 family |
| 54 | Pre-rRNA-processing protein TSR1 | 3 | 33 (31) | Q9VP47 | Ribosome biogeneis |
| 55 | Uncharacterized 50 kDa protein in type I retrotransposable element R1DM | 9 | 32 (31) | P16424 | CCHC-type zinc fingers |
| 56 | Ankyrin repeat and KH domain-containing protein mask | 2 | 40 (31) | Q9VCA8 | Mediator of receptor tyrosine kinase (RTK) signaling |
| 57 | Mediator of RNA polymerase II transcription subunit 24 | 2 | 31 (31) | Q9VSF2 | Transcription regulation |
| 58 | Dumpy | 28 | 47 (36) | Q8IQ18 | Apposition of dorsal and ventral imaginal disc-derived wing surfaces |
| 59 | Ribosome biogenesis protein BOP1 | 10 | 44 (30) | B4KQU8 | Ribosome biogenesis/rRNA processing |
| 60 | Protein 4.1 homolog | 3 | 51 (30) | Q9V8R9 | Septate junction |
| 61 | Protein hedgehog | 4 | 40 (30) | B4NJP3 | Cell-cell signaling involved in cell fate specification |
| 62 | Flap endonuclease GEN | 8 | 59 (31) | Q9VRJ0 | DNA catabolic process, endonucleolytic |
| 63 | Protein UBASH3A homolog | 3 | 36 (30) | Q9VCE9 | Not known |
| 64 | Nucleolar protein 6 | 34 | 40 (31) | B4GFN6 | RNA binding |
| 65 | Heat shock protein 83 | 2 | 35 (30) | O16068 | Stress response/cytoplasm |
| 66 | Serine/threonine-protein kinase PITSLRE | 2 | 34 (30) | Q9VPC0 | Negative regulator of the normal cell cycle progression |
| 67 | AT25667p | 5 | 43 (36) | Q8MSL6 | Acyltransferase |
| 68 | Tyrosine-protein kinase Fps85D | 6 | 45 (31) | P18106 | Actin filament bundle assembly |
| 69 | Bifunctional aminoacyl-tRNA synthetase | 3 | 38 (31) | P28668 | Protein biosynthesis |
| 70 | Protein white | 6 | 42 (36) | **P10090** | Membrane-spanning permease system (necessary for the transport of pigment precursors into pigment cells responsible for eye color) |
| 71 | Protein arginine N-methyltransferase 7 | 8 | 33 (30) | B3NP10 | Peptidyl-arginine methylation |
| 72 | Cohesin loading complex subunit SCC4 | 2 | 32 (30) | B4NKT1 | Cell cycle |
| 73 | Protein dopey-1 | 6 | 42 (30) | A1ZBE8 | Protein traffic between late Golgi and early endosomes |
| 74 | WD repeat-containing protein 55 | 5 | 41 (30) | B3P4F8 | WD repeat WDR55 family |
| 75 | POU domain protein 2, isoform B | 3 | 40 (30) | Q9VK71 | Transcription regulation |
| 76 | DNA polymerase alpha catalytic subunit | 9 | 34 (30) | P26019 | DNA damage/DNA repair/DNA replication |
| 77 | Protein disulfide-isomerase | 4 | 30 (30) | P54399 | Cell redox homeostasis |
| 78 | E3 ubiquitin-protein ligase Nedd-4 | 3 | 39 (31) | Q9VVI3 | Notch Signaling pathway/Ubl conjugation pathway |
| 79 | Diphenoloxidase subunit A3 | 6 | 41 (31) | Q8I1F6 | Melanin biosynthesis |
| 80 | Vesicle-fusing ATPase 2 | 4 | 39 (31) | P54351 | ER-Golgi transport |
| 81 | Tyrosine-protein kinase Abl | 4 | 38 (31) | P00522 | Axon guidance |
| 82 | Vitellogenin-1 | 3 | 36 (31) | P02843 | Yolk protein of eggs during embryogenesis |
| 83 | Serine/threonine-protein kinase ATR | 29 | 34 (31) | Q9VXG8 | DNA damage/repair |
| 84 | Calcium-binding mitochondrial carrier protein Aralar1 | 3 | 33 (31) | Q9VA73 | Transport |
| 85 | Protein dachsous | 7 | 49 (26) | Q24292 | Cell adhesion |
| 86 | WD repeat-containing protein on Y chromosome | 7 | 64 (31) | **B4F7L9** | Protein transport |
| 87 | Mitochondrial import inner membrane translocase subunit TIM50-B | 2 | 37 (30) | Q9W0S3 | Protein transport |
| 88 | Vinculin | 3 | 33 (30) | O46037 | Cell adhesion |
| 89 | DNA-directed RNA polymerase I subunit RPA1 | 8 | 53 (26) | P91875 | Transcription |
| 90 | Transcription initiation factor IIB | 2 | 32 (30) | Q9NHP7 | Transcription regulation |
| 91 | Protein distal antenna | 7 | 31 (30) | Q29CW2 | Transcription regulation |
| 92 | Membrane-associated protein Hem | 2 | 35 (31) | P55162 | Axonogenesis |
| 93 | Cytoplasmic tRNA 2-thiolation protein 2 | 8 | 33 (31) | B3NM45 | tRNA processing |
| 94 | Protein sidekick | 3 | 54 (31) | O97394 | Cell adhesion |
| 95 | Protein bride of sevenless | 2 | 38 (31) | Q24738 | G-protein coupled receptor protein signaling pathway |
| 96 | V-type proton ATPase subunit D 1 | 2 | 33 (31) | Q9V7D2 | Hydrogen ion transport |
| 97 | Eukaryotic translation initiation factor 3 subunit A | 2 | 32 (31) | B3LY22 | Protein biosynthesis |
| 98 | UPF0493 protein CG14299 | 4 | 55 (31) | Q9VE34 | Phosphoprotein |
| 99 | Another transcription unit protein | 5 | 50 (26) | **Q94546** | Phosphoprotein |
| 100 | Tenectin | 3 | 45 (36) | Q9VC00 | Extracellular matrix |
| 101 | Putative gamma-glutamylcyclotransferase CG2811 | 3 | 37 (31) | Q9W0Y2 | Acyltransferase |
| 102 | E3 ubiquitin-protein ligase HERC2 | 11 | 53 (26) | Q9VR91 | Protein modification; protein ubiquitination |
| 103 | Serine protease HTRA2, mitochondrial | 5 | 35 (31) | Q297U2 | Apoptosis |
| 104 | 28S ribosomal protein S10 | 3 | 33 (31) | Q9VFB2 | Translation |
| 105 | Netrin-A | 2 | 33 (31) | Q24567 | Differentiation/Neurogenesis |
| 106 | Apolipophorins | 5 | 55 (30) | Q9V496 | Wnt Signaling pathway |
| 107 | Calcium-dependent secretion activator | 2 | 39 (30) | Q9NHE5 | Exocytosis |
| 108 | Protein chiffon | 4 | 38 (30) | Q9NK54 | DNA replication/Activation of the chorion gene origins |
| 109 | Enhancer of yellow 2 transcription factor | 2 | 38 (30) | B4H2S0 | Transcription regulation |
| 110 | Transcription elongation factor B polypeptide 3 | 3 | 37 (30) | Q9VCP0 | Transcription regulation |
| 111 | Histone-lysine N-methyltransferase trithorax | 10 | 36 (30) | **P20659** | Transcription regulation |
| 112 | CG15580, isoform A | 3 | 47 (36) | Q9VNK8 | Protein binding |
| 113 | Putative gustatory receptor 22c | 2 | 33 (30) | P58952 | G-protein coupled receptor protein signaling pathway |
| 114 | Sterile alpha and TIR motif-containing protein 1 | 2 | 42 (30) | Q6IDD9 | Innate immunity |
| 115 | WD repeat-containing protein 55 homolog | 7 | 39 (30) | **Q8T088** | WD repeat WDR55 family. |
| 116 | Protein three rows | 9 | 39 (26) | Q6V3V8 | Malpighian tubule morphogenesis |
| 117 | Putative ribosomal RNA methyltransferase CG5220 | 7 | 33 (30) | Q9VEP1 | rRNA processing |
| 118 | Spectrin alpha chain | 2 | 31 (30) | P13395 | Cell shape |
| 119 | Polycomb group protein Psc | 10 | 63 (32) | P35820 | chromatin remodeling |
| 120 | Nuclear hormone receptor HR96 | 5 | 46 (32) | Q24143 | Transcription regulation |
| 121 | Neurogenic locus Notch protein | 4 | 44 (32) | P07207 | Notch signaling pathway |
| 122 | Chromosomal serine/threonine-protein kinase JIL-1 | 5 | 43 (32) | Q9V3I5 | Transcription regulation |
| 123 | Protein bowel | 3 | 42 (32) | Q9VQU9 | Imaginal disc-derived leg joint morphogenesis |
| 124 | Flotillin-2 | 4 | 41 (32) | O61492 | Cell adhesion |
| 125 | Ring canal kelch protein | 5 | 41 (32) | Q04652 | Differentiation/Oogenesis |
| 126 | Plexin-B | 3 | 41 (32) | Q9V4A7 | Differentiation/Oogenesis |
| 127 | Ubiquitin carboxyl-terminal hydrolase 36 | 3 | 41 (32) | B4KXJ5 | Ubl conjugation pathway |
| 128 | E3 ubiquitin-protein ligase hyd | 6 | 40 (32) | P51592 | Ubl conjugation pathway |
| 129 | Calpain-D | 2 | 39 (32) | P27398 | Nervous system development/eye |
| 130 | V-type proton ATPase subunit d 1 | 4 | 40 (30) | Q9W4P5 | Hydrogen ion transport |
| 131 | Eukaryotic translation initiation factor 3 subunit I | 2 | 36 (32) | B4LUA5 | Protein biosynthesis |
| 132 | Integrin alpha-PS1 | 4 | 36 (32) | Q24247 | Cell adhesion/a receptor for laminin |
| 133 | Neuropathy target esterase sws | 2 | 35 (32) | B4JLX2 | Neurogenesis |
| 134 | Defective chorion-1 protein, FC125 isoform | 2 | 34 (32) | P18169 | Eggshell chorion assembly |
| 135 | Protein ELYS | 3 | 34 (32) | Q9VWE6 | ELYS/MEL-28 family |
| 136 | Nucleosome-remodeling factor subunit NURF301 | 10 | 51 (26) | Q9W0T1 | Transcription regulation |
| 137 | N-acetyltransferase eco | 2 | 33 (32) | Q9VS50 | Cell cycle |
| 138 | Transcription elongation factor S-II | 2 | 33 (32) | P20232 | Transcription regulation |
| 139 | MAP kinase-activating death domain protein | 6 | 33 (32) | Q9VXY2 | Apoptosis/activation of MAPK activity |
| 140 | Furin-like protease 1, isoforms 1/1-X/2 | 3 | 32 (32) | P26016 | Proteolysis |
| 141 | RNA helicase armi | 6 | 32 (26) | Q6J5K9 | RNA-mediated gene silencing |
| 142 | Probable DNA mismatch repair protein Msh6 | 5 | 44 (29) | **Q9VUM0** | Post-replicative DNA-mismatch repair |
| 143 | Serine proteinase stubble | 4 | 40 (29) | Q05319 | Actin filament bundle assembly |
| 144 | La-related protein | 6 | 38 (29) | Q9VAW5 | Mitochondrion inheritance |
| 145 | AP-2 complex subunit alpha | 5 | 35 (29) | P91926 | Endocytosis |
| 146 | Protein abrupt | 3 | 33 (29) | Q24174 | Transcription regulation |
| 147 | Spindle assembly abnormal protein 6 | 7 | 31 (29) | Q9VAC8 | Cell cycle |
| 148 | Protein sevenless | 5 | 45 (30) | P13368 | Sensory transduction/Receptor for an extracellular signal required to instruct a cell to differentiate into an R7 photoreceptor |
| 149 | RING finger protein unkempt | 5 | 43 (30) | Q86B79 | Essential for late larval and early pupal development |
| 150 | Retrovirus-related Pol polyprotein from type-2 retrotransposable element R2DM | 5 | 41 (30) | P16423 | RNA-dependent DNA replication |
| 151 | Angiotensin-converting enzyme-related protein | 4 | 39 (30) | Q9VLJ6 | Heart development/specific maturation |
| 152 | Spectrin beta chain | 8 | 38 (30) | Q00963 | Actin filament capping |
| 153 | Protein flightless-1 | 5 | 36 (30) | Q24020 | Flight behavior/Structural role in indirect flight muscle |
| 154 | FK506-binding protein 59 | 3 | 35 (30) | Q9VL78 | Phototransduction; inhibits or prevents Ca^2+^ induced stimulation of the trpl ion channel |
| 155 | Dystrophin | 10 | 38 (26) | Q9VDW6 | Anchoring the cytoskeleton to the plasma membrane |
| 156 | Protein slit | 4 | 32 (30) | P24014 | Differentiation/Neurogenesis |
| 157 | 40S ribosomal protein S23 | 6 | 31 (30) | Q8T3U2 | Translation |
| 158 | E3 ubiquitin-protein ligase Su(dx) | 5 | 37 (29) | Q9Y0H4 | Notch signaling pathway/Ubl conjugation pathway |
| 159 | Tyrosine-protein kinase transmembrane receptor Ror | 3 | 31(30) | Q24488 | Central nervous system development |
| 160 | Histone-lysine N-methyltransferase trithorax | 11 | 41 (29) | Q24742 | Transcription regulation |
| 161 | Collagen alpha-1(IV) chain | 4 | 33 (29) | P08120 | Dorsal closure |
| 162 | Nuclear pore complex protein Nup88 | 4 | 31 (29) | Q9GYU8 | Immunity/Antimicrobial humoral response |
| 163 | Retrovirus-related Gag polyprotein from transposon HMS-Beagle | 4 | 30 (29) | Q967S7 | Strongly basic protein |
| 164 | Probable glutaminyl-tRNA synthetase | 6 | 32 (28) | Q9Y105 | Protein biosynthesis |
| 165 | Maternal protein tudor | 3 | 48 (29) | P25823 | Differentiation/Oogenesis |
| 166 | General transcription factor IIF subunit 1 | 5 | 44 (29) | Q05913 | Positive regulation of transcription |
| 167 | Mitosis initiation protein fs(1)Ya | 2 | 40 (29) | P25028 | Cell cycle/Cell division/Mitosis |
| 168 | Laminin subunit alpha | 8 | 46 (29) | Q00174 | Cell adhesion |
| 169 | E3 ubiquitin-protein ligase highwire | 8 | 52 (26) | Q9NB71 | Ubl conjugation pathway |
| 170 | Trehalase | 3 | 30 (29) | Q9W2M2 | Trehalose metabolic process |
| 171 | Serine hydrolase | 3 | 39 (29) | O18391 | Detoxification/Digestion |
| 172 | Protein snail | 3 | 37 (29) | P08044 | Essential for the correct specification of ventral-dorsal patterns |
| 173 | Protein male-specific lethal-3 | 3 | 36 (29) | P50536 | Chromatin assembly or disassembly |
| 174 | T-complex protein 1 subunit gamma | 3 | 33 (29) | P48605 | Mitotic spindle organization |
| 175 | Cleavage and polyadenylation specificity factor subunit1 | 2 | 30 (29) | Q9V726 | mRNA processing |
| 176 | Uncharacterized protein CG42248 | 3 | 30 (29) | Q9W5D0 | Phosphoprotein |
| 177 | CG18255-PA | 6 | 45 (37) | Q8MLD9 | Not known |
| 178 | Endoribonuclease Dcr-1 | 3 | 30 (28) | Q9VCU9 | RNA-mediated gene silencing |
| 179 | rRNA-processing protein EBP2 | 2 | 30 (28) | Q9V9Z9 | Ribosome biogenesis |
| 180 | DOMON domain-containing protein CG14681 | 6 | 29 (28) | Q9VGY6 | Catecholamine metabolic process/Histidine catabolic process |
| 181 | DNA mismatch repair protein spellchecker 1 | 3 | 30 (29) | P43248 | DNA damage/DNA repair |
| 182 | HEAT repeat-containing protein 1 homolog | 12 | 29 (28) | Q9VM75 | Ribosome biogenesis/rRNA processing |
| 183 | Larval serum protein 1 beta chain | 3 | 29 (28) | P11996 | A store of amino acids for synthesis of adult proteins |
| 184 | Major heat shock 70 kDa protein Ba | 7 | 43 (28) | Q8INI8 | Stress response |
| 185 | Centrosomin | 3 | 47 (32) | P54623 | Central nervous system development |
| 186 | Putative 1-phosphatidylinositol-3-phosphate 5-kinase | 2 | 29 (28) | O96838 | Cellular protein metabolic process |
| 187 | Retrovirus-related Pol polyprotein from transposon 297 | 11 | 30 (28) | P20825 | Aspartyl protease |
| 188 | Bloom syndrome protein | 4 | 29 (28) | Q9VGI8 | DNA replication |
| 189 | CG13917 | 3 | 42 (36) | Q9W0D3 | Protein binding |
| 190 | Dystrophin, isoform E | 10 | 38 (36) | Q7YU29 | Establishment of cell polarity |
| 191 | Vitellogenin | 4 | 33 (24) | Q05808 | Lipid transport |
| 192 | Calpain-C | 3 | 43 (29) | Q9VXH6 | Proteolysis |
| 193 | E3 ubiquitin-protein ligase Smurf1 | 5 | 39 (29) | Q9V853 | Ubl conjugation pathway |
| 194 | Ribosomal RNA processing protein | 5 | 35 (29) | Q9VJZ7 | rRNA processing |
| 195 | Protein tamozhennic | 3 | 32 (29) | Q9W1A4 | Multicellular organismal development |
| 196 | Frizzled-2 | 6 | 32 (29) | Q9VVX3 | Wnt signaling pathway |
| 197 | Pol polyprotein | 12 | 41 (36) | **O76326** | DNA integration |
| 198 | Fructose-bisphosphate aldolase | 3 | 159 (40) | **P07764** | Glycolysis |
| 199 | Actin, larval muscle | 3 | 119 (40) | **P02574** | Highly conserved proteins that are involved in various types of cell motility and are ubiquitously expressed in all eukaryotic cells |
| 200 | Glyceraldehyde-3-phosphate dehydrogenase 2 | 2 | 79 (40) | **P07487** | Glycolysis |
| 201 | CG7289 | 2 | 66 (40) | Q9VQ60 | Not known |
| 202 | CG3699 | 3 | 42 (40) | **Q9U1L2** | Oxidation reduction |
| 203 | LD22412p | 2 | 58 (40) | Q8T078 | Dendrite morphogenesis |
| 204 | CG12252 | 2 | 54 (40) | Q9W147 | Hydrolase |
| 205 | CG17255, isoform A | 2 | 54 (40) | Q9W2U7 | Entrainment of circadian clock |
| 206 | Histone-lysine N-methyltransferase ash1 | 3 | 53 (40) | Q8MQX5 | Chromatin-mediated maintenance of transcription |
| 207 | Ubiquitin-protein ligase | 2 | 51 (40) | Q9NGB1 | Ubl conjugation pathway |
| 208 | Neurobeachin | 12 | 56 (26) | Q9W4E2 | Eye photoreceptor cell development/ Compound eye cone cell differentiation |
| 209 | Leucine-rich repeat-containing G protein-coupled receptor 2 | 6 | 36 (27) | Q9BN18 | G-protein coupled receptor signaling |
| 210 | Protein spint | 3 | 29 (26) | Q8MQW8 | Developmental protein, GTPase activation |
| 211 | **Strn-Mlck** | 6 | 45 (30) | A1ZA72 | Protein amino acid phosphorylation |
| 212 | Polycomb protein Asx | 4 | 36 (30) | Q9V727 | Transcription regulation/Sex comb  development |
| 213 | Pollux, isoform A | 2 | 48 (40) | Q9VNG9 | Cell adhesion mediated by integrin  /Regulation of Rab GTPase activity |
| 214 | H/ACA ribonucleoprotein complex | 3 | 41 (40) | **Q7KVQ0** | Ribosome biogenesis/rRNA processing |
| 215 | Kinesin-like protein Klp10A | 3 | 44 (40) | Q960Z0 | Cell cycle |
| 216 | LD27161p | 2 | 44 (40) | Q8MRI5 | Ovarian follicle cell development |
| 217 | Surf6-PA | 8 | 44 (36) | Q8I151 | Not known |
| 218 | Lethal (1) G0060, isoform A | 16 | 36 (36) | Q9W485 | Not known |
| 219 | Papilin | 13 | 73 (26) | Q868Z9 | Extracellular matrix organization |
| 220 | CG7516 | 6 | 48 (36) | Q9V3P2 | Protein binding |
| 221 | Srp72 | 5 | 44 (36) | Q9VDK7 | SRP-dependent cotranslational protein targeting to membrane |
| 222 | BcDNA.LD27873 | 2 | 36 (36) | Q9V3H9 | Phagocytosis, engulfment |
| 223 | GH14426p | 28 | 38 (36) | Q6NNA4 | Not known |
| 224 | LP12301p | 4 | 36 (36) | Q960H1 | Oxidation reduction |
| 225 | Aromatic-L-amino-acid decarboxylase | 7 | 62 (36) | Q7Z0J7 | Carboxylic acid metabolic process |
| 226 | CG14864, isoform A | 9 | 50 (37) | Q9VFA3 | Not known |
| 227 | Mekk1, isoform B | 2 | 44 (37) | Q8MSQ4 | MAPKKK cascade |
| 228 | CG12187 | 7 | 42 (37) | Q9VZY3 | Protein binding |
| 229 | SD02424p | 14 | 41 (37) | Q8MSS0 | Binding |
| 230 | CG5792, isoform C | 7 | 43 (36) | Q9VK58 | Not known |
| 231 | CG10631 | 3 | 39 (36) | Q9VIS5 | Nucleic acid binding |
| 232 | Flap endonuclease GEN | 7 | 42 (36) | Q9U9Q6 | DNA catabolic process, endonucleolytic |
| 233 | Nesprin | 3 | 37 (36) | Q71JA7 | Actin filament organization |
| 234 | Antimeros | 6 | 38 (36) | Q9VN55 | Protein binding |
| 235 | CG11008-PA | 7 | 42 (36) | Q8I174 | DNA binding |
| 236 | LD01527p | 2 | 42 (36) | Q9VJ35 | Mitotic spindle elongation |
| 237 | CG7971, isoform A | 9 | 37 (36) | Q7YZ99 | Nuclear mRNA splicing, via spliceosome |
| 238 | RE22456p | 6 | 49 (36) | Q8SXT9 | Contractile ring contraction involved in cell cycle cytokinesis |
| 239 | Ryanodine receptor 44F | 7 | 53 (26) | Q24498 | Calcium transport |
| 240 | Leucine-rich repeat protein soc-2 | 3 | 44 (33) | B5DX45 | Leucine-rich repeat |
| 241 | Metallothionein-1 | 3 | 58 (36) | P61873 | Detoxification |
| 242 | Protein timeless | 4 | 37 (36) | **O17482** | Biological rhythms |
| 243 | Protein serrate | 6 | 49 (26) | P18168 | Differentiation |
| 244 | LIM domain kinase 1 | 6 | 49 (26) | Q8IR79 | Actin cytoskeleton organization |
| 245 | Cadherin-related tumor suppressor | 8 | 46 (26) | P33450 | Cell adhesion |
| 246 | DNA topoisomerase 2 | 4 | 44 (26) | P15348 | DNA topological change |
| 247 | Putative vitellogenin receptor | 7 | 41 (26) | P98163 | Endocytosis |
| 248 | Laminin subunit beta-1 | 4 | 40 (26) | P11046 | Cell adhesion |
| 249 | Protein expanded | 3 | 39 (26) | Q07436 | Transcription regulation |
| 250 | Polycomb protein Scm | 4 | 39 (26) | Q9VHA0 | Transcription regulation |
| 251 | ATP-dependent RNA helicase vasa | 7 | 38 (26) | P09052 | Differentiation/ Oogenesis |
| 252 | Cytochrome P450 4g1 | 5 | 38 (26) | Q9V3S0 | Lipid metabolic process |
| 253 | ATP-dependent RNA helicase p62 | 4 | 36 (26) | P19109 | RNA-mediated gene silencing |
| 254 | Defective chorion-1 protein, FC177 | 8 | 34 (26) | P18171 | Eggshell chorion assembly |
| 255 | Ubiquitin carboxyl-terminal hydrolase 64E | 6 | 34 (26) | Q24574 | Ubl conjugation pathway |
| 256 | Serine/threonine-protein kinase Smg1 | 4 | 34 (26) | Q70PP2 | Nonsense-mediated mRNA decay |
| 257 | Zinc finger protein hangover | 6 | 33 (26) | Q9VXG1 | Response to ethanol |
| 258 | CAD protein | 4 | 33 (26) | P05990 | Pyrimidine biosynthesis |
| 259 | Dynein heavy chain, cytoplasmic | 12 | 38 (26) | P37276 | Motor for the intracellular retrograde motility of vesicles and organelles along microtubules |
| 260 | Neural-cadherin | 4 | 32 (26) | O15943 | Cell adhesion |
| 261 | Conserved oligomeric Golgi complex subunit 4 | 3 | 32 (26) | Q95TN4 | Protein transport |
| 262 | Gametogenetin-binding protein 2-like | 5 | 32 (26) | Q9VNG1 | Not known |
| 263 | DNA replication licensing factor Mcm6 | 3 | 32 (26) | Q29JI9 | Cell cycle |
| 264 | Lysine-specific demethylase 4B | 4 | 32 (26) | Q9V6L0 | Histone H3-K36 demethylation |
| 265 | Trifunctional purine biosynthetic protein adenosine-3 | 4 | 31 (26) | P00967 | Purine biosynthesis |
| 266 | Protein bcn92 | 3 | 31 (26) | P82116 | Not Known |
| 267 | Supporter of activation of yellow protein | 5 | 30 (26) | Q9VWF2 | Negative regulation of transcription, DNA-dependent |
| 268 | Homeobox protein prospero | 5 | 30 (26) | Q9U6A1 | Multicellular organismal development |
| 269 | Transcription initiation factor TFIID subunit 4 | 3 | 29 (26) | P47825 | Dendrite morphogenesis |
| 270 | Prominin-like protein | 2 | 29 (26) | P82295 | Integral to membrane |
| 271 | Modifier of mdg4 | 3 | 28 (26) | Q86B87 | Apoptosis |
| 272 | Lysozyme P | 3 | 28 (26) | P29615 | Cell wall macromolecule catabolic process |
| 273 | Muscle LIM protein Mlp84B | 2 | 28 (26) | Q24400 | Muscle organ development |
| 274 | Putative U5 small nuclear ribonucleoprotein 200 kDa helicase | 4 | 28 (26) | Q9VUV9 | mRNA processing |
| 275 | Ubiquitin-like protein 5 | 2 | 27 (26) | Q9V998 | Ubl conjugation pathway |
| 276 | Protein nullo | 3 | 27 (26) | P32845 | Cell-cell junction assembly |
| 277 | N6-adenosine-methyltransferase MT-A70-like protein | 3 | 27 (26) | Q9VCE6 | RNA methylation |
| 278 | Cytochrome P450 4ad1 | 2 | 27 (26) | Q9V4T3 | Oxidation reduction |
| 279 | Tyrosine-protein kinase PR2 | 6 | 27 (26) | Q9I7F7 | Protein amino acid phosphorylation |
| 280 | Eye-specific diacylglycerol kinase | 4 | 28 (26) | Q09103 | Phospholipid turnover within the photoreceptor |
| 281 | Opsin Rh6 | 2 | 27 (26) | O01668 | Sensory transduction |
| 282 | Serine/threonine-protein phosphatase 2B catalytic subunit 1 | 2 | 27 (26) | P48456 | Neurotransmitter secretion |
| 283 | JNK-interacting protein 3 | 3 | 27 (26) | Q9GQF1 | Regulation of JNK cascade |
| 284 | Paramyosin | 5 | 29 (25) | P35415 | Major structural component of many thick filaments isolated from invertebrate muscles |
| 285 | Alpha-(1,3)-fucosyltransferase C | 8 | 27 (26) | P83088 | Protein amino acid glycosylation |
| 286 | RNA-binding protein cabeza | 4 | 71 (36) | Q27294 | Nuclear mRNA splicing, via spliceosome |
| 287 | AT29074p | 2 | 37 (26) | Q6NNX7 | Type-B carboxylesterase/lipase family |
| 288 | CG9313 | 3 | 39 (36) | Q7KVQ2 | ATPase activity, uncoupled |
| 289 | LD41783p | 2 | 38 (36) | Q8SWR4 | Not known |
| 290 | Short spindle protein 4 | 8 | 58 (36) | A1ZAU8 | Cell cycle |
| 291 | Protein pecanex | 6 | 56 (36) | P18490 | Differentiation/Neurogenesis |
| 292 | PERQ amino acid-rich with GYF domain-containing protein CG11148 | 8 | 50 (36) | Q7KQM6 | Belongs to the PERQ family |
| 293 | Nuclear factor NF-kappa-B p110 subunit | 4 | 49 (36) | Q94527 | Immune response |
| 294 | Voltage-dependent calcium channel type D subunit alpha-1 | 5 | 38 (29) | Q24270 | Calcium transport |
| 295 | Serine/threonine-protein kinase polo | 4 | 40 (29) | P52304 | Cytokinesis |
| 296 | Mitoferrin | 4 | 61 (33) | Q9VAY3 | Mitochondrial iron ion transport |
| 297 | Protein extra-macrochaetae | 4 | 56 (33) | P18491 | R8 cell fate commitment |
| 298 | Transmembrane protein 41 | 5 | 50 (33) | Q9VX39 | Integral to membrane |
| 299 | Mediator of RNA polymerase II transcription subunit 26 | 4 | 55 (33) | Q29CV2 | Transcription regulation |
| 300 | Mediator of RNA polymerase II transcription subunit 13 | 8 | 35 (33) | Q7KTX8 | Transcription regulation |
| 301 | Putative epidermal cell surface receptor | 6 | 54 (33) | Q04164 | Instar larval development |
| 302 | MPN domain-containing protein CG4751 | 4 | 35 (33) | Q9VKJ1 | Probable protease |
| 303 | T-complex protein 1 subunit alpha | 6 | 40 (33) | P12613 | Mitotic spindle organization |
| 304 | Helicase domino | 4 | 50 (33) | Q9NDJ2 | Cell cycle |
| 305 | Female-specific protein transformer | 5 | 39 (33) | P11596 | Cell differentiation |
| 306 | Cadherin-87A | 3 | 35 (33) | Q9VGG5 | Cell adhesion |
| 307 | Muscle-specific homeobox protein tinman | 4 | 36 (33) | P22711 | Cardiac muscle cell differentiation |
| 308 | 60 kDa heat shock protein | 6 | 45 (33) | Q9VPS5 | Protein folding |
| 309 | Protein matrimony | 2 | 38 (33) | P83733 | Cell cycle |
| 310 | Protein FAM21 | 3 | 37 (33) | A1ZBW7 | Phosphoprotein |
| 311 | Protein slender lobes | 8 | 69 (33) | Q8INM3 | Multicellular organismal development |
| 312 | Borealin | 3 | 54 (33) | Q9VLD6 | Cell cycle/cytokinesis |
| 313 | Beta-amyloid-like protein | 2 | 54 (33) | P14599 | Differentiation/Neurogenesis |
| 314 | Protein cramped | 7 | 50 (33) | Q8MX88 | Regulation of transcription |
| 315 | Elongation factor G | 5 | 36 (33) | B4KKD5 | Protein biosynthesis |
| 316 | Enhancer of split m8 protein | 13 | 38 (33) | Q07291 | Cell differentiation |
| 317 | CCR4-NOT transcription complex | 4 | 38 (33) | Q9V3G6 | Phosphoprotein |
| 318 | NADPH-cytochrome P450 reductase | 8 | 30 (29) | Q27597 | Oxidation reduction |
| 319 | Mitochondrial import inner membrane translocase | 12 | 40 (33) | Q9W4V8 | Protein transport |
| 320 | UPF0171 protein CG8783 | 3 | 40 (33) | Q9VUB4 | Protein binding |
| 321 | Multidrug resistance protein homolog 49 | 4 | 39 (33) | Q00449 | Transport |
| 322 | ATP-dependent RNA helicase pitchoune | 4 | 39 (33) | Q9VD51 | Helicase/Hydrolase |
| 323 | Tyrosine-protein kinase hopscotch | 2 | 49 (30) | Q24592 | Transcription regulation |
| 324 | Potassium voltage-gated channel protein eag | 4 | 39 (33) | Q02280 | Differentiation/Ion transport/Neurogenesis |
| 325 | Open rectifier potassium channel protein 1 | 6 | 48 (33) | Q94526 | Ion transport |
| 326 | Serendipity locus protein H-1 | 8 | 47 (33) | P15619 | Multicellular organismal development |
| 327 | Lethal(2) giant larvae protein | 5 | 46 (33) | P08111 | Cell cycle |
| 328 | RNA-directed DNA polymerase from transposon X-element | 6 | 44 (33) | Q9NBX4 | RNA-dependent DNA replication |
| 329 | Soluble guanylate cyclase 88E | 12 | 38 (36) | Q8INF0 | cGMP biosynthesis |
| 330 | Tyrosine-protein phosphatase Lar | 9 | 45 (36) | P16621 | Cell adhesion |
| 331 | Hormone receptor 4 | 2 | 38 (33) | Q9W539 | Transcription regulation |
| 332 | Lethal(2) giant larvae protein | 5 | 46 (33) | P08111 | Cell cycle |
| 333 | Tyrosine-protein phosphatase Lar | 2 | 35 (33) | P16621 | Cell adhesion |
| 334 | Putative 115 kDa protein in type-1 retrotransposable element R1DM | 4 | 38 (29) | P16425 | RNA-dependent DNA replication |
| 335 | Homeotic protein spalt-major | 6 | 38 (29) | P39770 | [Transcription regulation](http://www.uniprot.org/keywords/KW-0805) |
| 336 | Disks large 1 tumor suppressor protein | 6 | 37 (33) | P31007 | Cell adhesion/Cell junction |
| 337 | Raf homolog serine/threonine-protein kinase phl | 8 | 42 (26) | P11346 | Border follicle cell migration |
| 338 | Putative mitochondrial inner membrane protein | 3 | 30 (26) | P91928 | Integral to mitochondrial inner membrane |
| 339 | Myosin heavy chain, muscle | 7 | 41 (28) | P05661 | Epithelial cell migration, open tracheal system |
| 340 | Transcription factor grauzone | 3 | 30 (28) | Q9U405 | Meiosis |
| 341 | Calbindin-32 | 3 | 30 (28) | P41044 | Calcium ion binding |
| 342 | rRNA 2'-O-methyltransferase fibrillarin | 3 | 30 (28) | Q9W1V3 | rRNA processing |
| 343 | Phenylalanyl-tRNA synthetase | 4 | 30 (28) | O16129 | Protein biosynthesis |
| 344 | Slowpoke-binding protein | 5 | 60 (32) | Q8IPH9 | Regulator of calcium channel/regulation of synaptic transmission |
| 345 | Insuline-like receptor | 18 | 80 (26) | P09208 | Differentiation/Growth regulation/Neurogenesis |
| 346 | Regulator of nonsense transcripts 1 homolog | 8 | 67 (26) | Q9CVYS3 | Nuclear-transcribed mRNA catabolic process, nonsense-mediated decay |
| 347 | Nucleic-acid-binding protein from mobile element jocker | 6 | 60 (26) | P21330 | Nucleic acid binding |
| 348 | Protein decapentaplegic | 8 | 54 (26) | P07713 | BMP (bone morphogenetic protein) signaling pathway (a series of molecular signal generated as a consequence of any member of the BMP family binding to a cell surface receptor) |
| 349 | Vesicular-fusion ATPase 1 | 5 | 47 (26) | P46461 | ER-Golgi transport |
| 350 | Protein crumbs | 5 | 45 926) | P10040 | Differentiation |
| 351 | Cytochrome-P450 4p1 | 3 | 41 (26) | Q9V558 | Oxidation reduction |
| 352 | Elongation factor 1-alpha | 2 | 27 (25) | P27592 | Protein biosynthesis ***Onchocerca volvulus*** |
| 353 | signal recognition particle 72 kDa protein | 2 | 32 (25) | P49965 | SRP-dependent cotranslational protein targeting to membrane ***Schistosoma mansoni* (Blood fluke)** |
| 354 | Sex-determining transformer protein 2 | 3 | 33 (24) | Q9NIW4 | Sexual differentiation ***Caenorhabditis Remanei* (Caenorhabditis vulgaris)** |
| 355 | Mite group 2 allergen Der f 2 | 2 | 28 (24) | Q00855 | Extracellular region ***Dermatophagoides Farinae* (American house dust mite)** |
| 356 | Tropomyosin | 3 | 27 (24) | O02389 | Central role in the calcium dependent regulation of muscle contraction ***Chlamys nipponensis akazara* (Akazara scallop) (Japanese scallop)** |
| 357 | Guanine nucleotide-binding protein G(s) subunit alpha | 3 | 28 (25) | P30669 | G-protein coupled receptor protein signaling pathway ***Schistosoma mansoni*(Blood fluke)** |
| 358 | Toxin Aah6 | 2 | 27 (25) | P56743 | Defense response pathogenesis ***Androctonus australis* (Sahara scorpion)** |
| 359 | G2/mitotic-specific cyclin-B | 9 | 31 (24) | P18063 | Cell cycle ***Asterina pectinifera*** |
| 360 | Heat shock protein 70 | 2 | 27 (25) | P91902 | Stress response ***Ceratitis capitata* (Mediterranean fruit fly) (Tephritis capitata)** |
| 361 | Glutamate carboxypeptidase 2 homolog | 7 | 40 (25) | Q5WN23 | Proteolysis ***Caenorhabditis briggsae*** |
| 362 | Muscle calcium channel subunit alpha-1 | 3 | 31 (25) | Q25452 | Calcium transport ***Musca domestica* (House fly)** |
| 363 | Hemocytin | 6 | 30 (25) | P98092 | Cell adhesion ***Bombyx mori* (silk moth)** |
| 364 | Dynein beta chain, ciliary | 22 | 55 (26) | P23098 | Cilium biogenesis/degradation ***Tripneustes gratilla* (Hawaian sea urchin)** |
| 365 | Guanine nucleotide-binding protein alpha-17 subunit | 3 | 27 (25) | Q86FX7 | Chemotaxi/G-protein coupled receptor protein signaling pathway |
| 366 | Hemocyanin subunit B | 2 | 26 (25) | Q8IFJ8 | Oxygen transport ***Scutigera coleoptrata* (House centipede)** |
| 367 | 227 kDa spindle- and centromere-associated protein | 6 | 35 (24) | O61308 | Cell cycle ***Parascaris univalens*** |
| 368 | Extracellular matrix protein 3 | 9 | 33 (24) | Q9GV77 | Cell adhesion ***Lytechinus variegatus*** |
| 369 | Annexin-B12 | 5 | 32 (24) | P26256 | Calcium-dependent phospholipid binding ***Hydra attenuata*** |
| 370 | Vitellogenin-1 | 3 | 26 (25) | Q9U8M0 | Lipid transport ***Periplaneta Americana* (American cockroach) (Blatta americana)** |
| 371 | Arginine kinase | 2 | 30 (25) | P51541 | Phosphorylation ***Limulus polyphemus* (Atlantic horseshoe crab)** |
| 372 | Syntaxin | 3 | 35 (25) | Q16932 | Neurotransmitter transport ***Aplysia californica* (California sea hare)** |
| 373 | Vitellogenin-A1 | 4 | 32 (25) | Q16927 | Lipid transport ***Aedes aegypti* (Yellowfever mosquito)** |
| 374 | Armadillo segment polarity protein | 2 | 27 (24) | Q7QHW5 | Cell adhesion/Wnt signaling pathway ***Anopheles gambiae* (African malaria mosquito)** |
| 375 | Toxin BeM14 | 2 | 26 (24) | P09982 | Defense response ***Buthus eupeus*** |
| 376 | DNA (cytosine-5)-methyltransferase PliMCI | 3 | 31 (25) | Q27746 | Methylates CpG residues ***Paracentrotus lividus* (Common sea urchin)** |
| 377 | Cathepsin B-like cysteine proteinase | 2 | 29 (25) | P43157 | Proteolysis ***Schistosoma japonicum* (Blood fluke)** |
| 378 | Ring canal kelch homolog | 3 | 26 (25) | Q70JS2 | Ring canal kelch homolog ***Anopheles Stephensi* (Indo-Pakistan malaria mosquito)** |
| 379 | Venom allergen 5.01 | 3 | 26 (24) | P35781 | **Secreted *Vespa crabro*** |
| 380 | Kinesin light chain | 8 | 34 (25) | Q05090 | Microtubule motor activity ***Strongylocentrotus purpuratus*** |
| 381 | Cadmium-metallothionein | 4 | 27 (24) | P81695 | High content of cysteine residues that bind various heavy metals ***Eisenia foetida*** |
| 382 | DNA-directed RNA polymerase II subunit RPB1 | 3 | 29 (25) | P35074 | Transcription from RNA polymerase II promoter ***Caenorhabditis briggsae*** |
| 383 | Fasciclin-2 | 5 | 29 (25) | P22648 | Cell adhesion ***Schistocerca americana* (American grasshopper)** |
| 384 | Histone H1, early embryonic | 3 | 26 (25) | P19375 | Nucleosome assembly ***Strongylocentrotus purpuratus* (Purple sea urchin)** |
| 385 | Antichymotrypsin-1 | 5 | 27 (24) | Q03383 | Inhibits chymotrypsin activity ***Bombyx mori* (silk moth)** |
| 386 | Potassium channel toxin TsTXK-beta | 3 | 33 (26) | P69940 | Pathogenesis ***Tityus serrulatus*(Brazilian scorpion)** |
| 387 | Major antigen | 5 | 30 (26) | P21249 | Myofibrillar protein ***Onchocerca volvulus*** |
| 388 | Metallothionein 20-I isoforms A and B | 6 | 29 (26) | P80251 | Cellular sequestration of toxic metal ions **Mytilus edulis** |
| 389 | Nitrophorin-1 | 2 | 28 (24) | Q26239 | Vasodilation ***Rhodnius prolixus*** |
| 390 | 60S acidic ribosomal protein P0 | 8 | 28 (26) | Q9U3U0 | Ribosome biogenesis ***Ceratitis capitata* (Mediterranean fruit fly) (Tephritis capitata)** |
| 391 | Asparaginyl-tRNA synthetase, cytoplasmic | 5 | 28 (26) | P10723 | Protein biosynthesis ***Brugia malayi*** |
| 392 | Trifunctional purine biosynthetic protein adenosine-3 | 2 | 27 (26) | Q26255 | Purine biosynthesis ***Chironomus tentans*** |
| 393 | Triosephosphate isomerase | 4 | 27 (26) | Q9GTX8 | Fatty acid biosynthesis ***Taenia solium*** |
| 394 | 60S ribosomal protein L13 | 3 | 27 (26) | O46157 | Ribonucleoprotein ***Lumbricus rubellus*** |
| 395 | Potassium channel toxin alpha-KTx 2.6 | 5 | 33 (25) | P59849 | Pathogenesis ***Centruroides limbatus*** |
| 396 | Aminopeptidase N | 6 | 29 (25) | Q10737 | Proteolysis ***Haemonchus contortus* (Barber pole worm)** |
| 397 | Severin | 13 | 27 (25) | Q24800 | Actin filament capping ***Echinococcus granulosus*** |
| 398 | Serine/threonine-protein kinase zyg-1 | 5 | 29 (24) | Q621J7 | Cell cycle ***Caenorhabditis briggsae*** |
| 399 | Metallothionein-B | 10 | 27 (26) | Q26496 | Metal ion binding ***Sphaerechinus granularis*** |
| 400 | Acetylcholine receptor subunit alpha-L1 | 4 | 31 (25) | P23414 | Ion transport ***Schistocerca gregaria* (Desert locust)** |
| 401 | 78 kDa glucose-regulated protein | 2 | 32 (25) | Q16956 | Assembly of multimeric protein complexes inside the ER ***Aplysia californica* (California sea hare)** |
| 402 | Metallothionein | 6 | 29 (25) | O02033 | Metal ion binding ***Lytechinus pictus* (Painted sea urchin)** |
| 403 | Stress-activated protein kinase JNK-1 | 2 | 29 (25) | Q9U6D2 | JNK cascade ***Ancylostoma caninum* (Dog hookworm)** |
| 404 | Vitellogenin | 5 | 28 (25) | Q27309 | Lipid transport ***Bombyx mori* (silk moth)** |
| 405 | Adhesive plaque matrix protein | 2 | 27 (25) | Q25460 | Adhesiveness to the mussel's foot ***Mytilus edulis* (Blue mussel)** |
| 406 | Apolipophorins | 5 | 31 (24) | Q9U943 | Wnt receptor signaling pathway ***Locusta migratoria*** |
