# Supplementary material for: Diet-Induced Over-Expression of Flightless-I Protein and Its Relation to Flightlessness in Mediterranean Fruit Fly, Ceratitis capitata
Source: PLoS One. 2013 Dec 3;8(12):e81099. doi: 10.1371/journal.pone.0081099 (PMC3849048; doi:10.1371/journal.pone.0081099)
Supplement: Table S3 — A list of 239 over-expressed proteins detected in pupae B whose adult flies showed a low flight rate. (DOC) [file pone.0081099.s003.doc]

**Supporting Information (SI)**

**Diet-induced over-expression of flightless-I protein and its relation to flightlessness in Mediterranean fruit fly, *Ceratitis capitata***

Il Kyu Cho^1^, Chiou Ling Chang^2^ and Qing X. Li^1^*

^1^ Department of Molecular Biosciences and Bioengineering, University of Hawaii, Honolulu, Hawaii, USA.

^2^ U.S. Pacific Basin Agricultural Research Center, Hilo, Hawaii, USA.

**Table S3**. **A list of 239 over-expressed proteins detected in pupae B whose adult flies showed a low flight rate**. The LC-MS/MS data were matched with *Drosophila melanogaster* database via MASCOT for the sequence alignments.

| No. | Protein names | No. of matched peptides | Mascot Scores (p=0.05) | Accession numbers | Biological functions |
| --- | --- | --- | --- | --- | --- |
| 1 | Ribosomal L1 domain-containing protein CG13096 | 8 | 47 (30) | Q9VLK2 | RNA processing/ribosome |
| 2 | Methyltransferase-like protein 13 | 6 | 40 (30) | Q29LW1 | Metabolic process |
| 3 | Diacylglycerol kinase eta | 10 | 45 (30) | B3LXF2 | G-protein coupled receptor protein signaling pathway |
| 4 | Ras-related protein Ral-a | 7 | 38 (30) | P48555 | Negative regulation of JNK cascade |
| 5 | Protein wings apart-like | 7 | 52 (30) | Q9W517 | Chromosome partition |
| 6 | Ribosome biogenesis protein WDR12 | 10 | 36 (30) | B4KKN1 | Ribosome biogenesis/rRNA processing |
| 7 | Polypeptide N-acetyl galactosaminyl transferase 35A | 3 | 35 (30) | Q8MVS5 | Essential glycotransferase |
| 8 | Ecdysone-induced protein 75B, isoform B | 3 | 35 (30) | P13055 | Regulation of ecdysone-triggered gene hierarchies |
| 9 | Kinesin light chain | 2 | 34 (30) | P46824 | Microtubule-associated force-producing protein that may play a role in organelle transport |
| 10 | Putative transcription factor capicua | 3 | 37 (30) | Q9U1H0 | Transcription regulation |
| 11 | Nucleolar complex protein 3 | 13 | 58 (31) | Q9VI82 | Binding |
| 12 | WD repeat-containing protein on Ychromosome | 8 | 48 (31) | B7FF08 | Repeat |
| 13 | DNA topoisomerase 1 | 5 | 41 (31) | P30189 | DNA topological change |
| 14 | Mediator of RNA polymerase II transcription subunit 1 | 6 | 53 (31) | Q9VP05 | Regulation of transcription from RNA polymerase II promoter |
| 15 | Protein KIAA0664 homolog | 2 | 36 (31) | B4MY63 | KIAA0664/TIF31 family |
| 16 | ATP-dependent RNA helicase CG8611 | 2 | 34 (31) | Q86B47 | ATP-dependent RNA helicase/hydrolase |
| 17 | Synapsin | 6 | 31 (31) | Q24546 | Behavior/cell junction |
| 18 | PHD finger protein rhinoceros | 13 | 72 (26) | Q7YZH1 | Negative regulator of the EGFR/Ras/MAPK signaling pathway during eye development |
| 19 | Protein dopey-1 | 7 | 48 (31) | Q292H2 | Protein transport |
| 20 | Protein still life, isoforms C/siF type 2 | 6 | 45 (30) | P91620 | Intracellular signaling cascade/regulation of Rho protein signal transduction |
| 21 | Fasciclin-1 | 3 | 42 (31) | P10674 | Cell adhesion |
| 22 | Nucleolar protein 6 | 3 | 36 (31) | B4NIM9 | RNA binding |
| 23 | Protein bric-a-brac 1 | 19 | 35 (31) | Q9W0K7 | Transcription regulation/nucleus |
| 24 | RNA-binding protein CG14230 | 5 | 41 (31) | Q9VWD4 | RNA binding |
| 25 | Nuclear RNA export factor 2 | 3 | 41 (31) | Q9VV73 | mRNA transport (export of mRNA from the nucleus to the cytoplasm) |
| 26 | Pescadillo | 4 | 37 (31) | B3N8H0 | Ribosome biogenesis/rRNA processing |
| 27 | Protein unc-80 | 5 | 37 (31) | Q9VB11 | Cation homeostasis |
| 28 | Regulator of telomere elongation helicase 1 | 5 | 35 (31) | B4I0K4 | DNA damage/DNA repair |
| 29 | Axoneme-associated protein mst101(1) | 3 | 34 (31) | Q08695 | Sperm axoneme assembly |
| 30 | DNA-binding protein D-ETS-6 | 3 | 32 (31) | P29776 | dendrite morphogenesis/nucleus |
| 31 | Polypeptide N-acetyl galactosaminyl transferase 1 | 2 | 31 (31) | Q6WV20 | Oligosaccharide biosynthetic process |
| 32 | Epidermal growth factor receptor | 4 | 38 (31) | P04412 | Muscle cell fate specification |
| 33 | Protein spire | 3 | 37 (31) | Q9U1K1 | Transport |
| 34 | Protein suppressor of white apricot | 3 | 36 (31) | P12297 | Transcription regulation |
| 35 | DM7 family protein CG15332 | 5 | 34 (31) | Q9W3M2 | DM7 family |
| 36 | Pre-rRNA-processing protein TSR1 | 3 | 33 (31) | Q9VP47 | Ribosome biogenesis |
| 37 | Uncharacterized 50 kDa protein in type I retrotransposable element R1DM | 9 | 32 (31) | P16424 | CCHC-type zinc fingers |
| 38 | Mediator of RNA polymerase II transcription subunit 24 | 2 | 31 (31) | Q9VSF2 | Transcription regulation |
| 39 | Dumpy | 8 | 47 (36) | Q8IQ18 | Apposition of dorsal and ventral imaginal disc-derived wing surfaces/chitin-based cuticle attachment to epithelium |
| 40 | Ribosome biogenesis protein BOP1 | 10 | 44 (30) | B4KQU8 | Ribosome biogenesis/rRNA processing |
| 41 | Protein hedgehog | 4 | 40 (30) | B4NJP3 | Cell-cell signaling involved in cell fate specification |
| 42 | Flap endonuclease GEN | 8 | 59 (31) | Q9VRJ0 | DNA catabolic process, endonucleolytic |
| 43 | Protein UBASH3A homolog | 3 | 36 (30) | Q9VCE9 | Not known |
| 44 | Nucleolar protein 6 | 34 | 40 (31) | B4GFN6 | RNA binding |
| 45 | Heat shock protein 83 | 2 | 35 (30) | O16068 | Stress response |
| 46 | Serine/threonine-protein kinase PITSLRE | 2 | 34 (30) | Q9VPC0 | Negative regulator of the normal cell cycle progression |
| 47 | AT25667p | 5 | 43 (36) | Q8MSL6 | Acyltransferase |
| 48 | Tyrosine-protein kinase Fps85D | 6 | 45 (31) | P18106 | Actin filament bundle assembly |
| 49 | Bifunctional aminoacyl-tRNA synthetase (Glutamyl-tRNA synthetase, Prolyl-tRNA synthetase) | 3 | 38 (31) | P28668 | Protein biosynthesis |
| 50 | Protein white | 6 | 42 (36) | P10090 | Transport (membrane-spanning permease system) |
| 51 | Protein arginine N-methyltransferase 7 | 8 | 33 (30) | B3NP10 | Peptidyl-arginine methylation |
| 52 | Cohesin loading complex subunit SCC4 | 2 | 32 (30) | B4NKT1 | Cell cycle |
| 53 | Protein dopey-1 | 6 | 42 (30) | A1ZBE8 | Protein traffic between late Golgi and early endosomes |
| 54 | WD repeat-containing protein 55 | 5 | 41 (30) | B3P4F8 | WD repeat WDR55 family |
| 55 | POU domain protein 2, isoform B | 3 | 40 (30) | Q9VK71 | Transcription regulation |
| 56 | DNA polymerase alpha catalytic subunit | 9 | 34 (30) | P26019 | DNA damage/DNA repair/DNA replication |
| 57 | Protein disulfide-isomerase | 4 | 30 (30) | P54399 | Cell redox homeostasis |
| 58 | E3 ubiquitin-protein ligase Nedd-4 | 3 | 39 (31) | Q9VVI3 | Notch signaling pathway/Ubl conjugation pathway |
| 59 | Diphenoloxidase subunit A3 | 6 | 41 (31) | Q8I1F6 | Melanin biosynthesis |
| 60 | Vesicle-fusing ATPase 2 | 4 | 39 (31) | P54351 | ER-Golgi transport |
| 61 | Vitellogenin-1 | 3 | 36 (31) | P02843 | Yolk protein of eggs during embryogenesis |
| 62 | Calcium-binding mitochondrial carrier protein Aralar1 | 3 | 33 (31) | Q9VA73 | Transport |
| 63 | Protein dachsous | 7 | 49 (26) | Q24292 | Cell adhesion |
| 64 | WD repeat-containing protein on Ychromosome | 7 | 64 (31) | B4F7L9 | Protein transport |
| 65 | Mitochondrial import inner membrane translocase subunit TIM50-B | 2 | 37 (30) | Q9W0S3 | Protein transport |
| 66 | Vinculin | 3 | 33 (30) | O46037 | Cell adhesion |
| 67 | DNA-directed RNA polymerase I subunit RPA1 | 8 | 53 (26) | P91875 | Transcription |
| 68 | Transcription initiation factor IIB | 2 | 32 (30) | Q9NHP7 | Transcription regulation |
| 69 | Protein distal antenna | 7 | 31 (30) | Q29CW2 | Transcription regulation |
| 70 | Membrane-associated protein Hem | 2 | 35 (31) | P55162 | Axonogenesis |
| 71 | Cytoplasmic tRNA 2-thiolation protein 2 | 8 | 33 (31) | B3NM45 | tRNA processing |
| 72 | Protein sidekick | 3 | 54 (31) | O97394 | Cell adhesion |
| 73 | Protein bride of sevenless | 2 | 38 (31) | Q24738 | G-protein coupled receptor protein signaling pathway |
| 74 | V-type proton ATPase subunit D 1 | 2 | 33 (31) | Q9V7D2 | Hydrogen ion transport |
| 75 | Eukaryotic translation initiation factor 3 subunit A | 2 | 32 (31) | B3LY22 | Protein biosynthesis |
| 76 | UPF0493 protein CG14299 | 4 | 55 (31) | Q9VE34 | Phosphoprotein |
| 77 | Tenectin | 3 | 45 (36) | Q9VC00 | Extracellular matrix |
| 78 | Putative gamma-glutamylcyclotransferase CG2811 | 3 | 37 (31) | Q9W0Y2 | Acyltransferase |
| 79 | Serine protease HTRA2, mitochondrial | 5 | 35 (31) | Q297U2 | Apoptosis |
| 80 | 28S ribosomal protein S10 | 3 | 33 (31) | Q9VFB2 | Translation |
| 81 | Netrin-A | 2 | 33 (31) | Q24567 | Differentiation/Neurogenesis |
| 82 | Calcium-dependent secretion activator | 2 | 39 (30) | Q9NHE5 | Exocytosis |
| 83 | Enhancer of yellow 2 transcription factor | 2 | 38 (30) | B4H2S0 | Transcription regulation |
| 84 | Transcription elongation factor B polypeptide 3 | 3 | 37 (30) | Q9VCP0 | Transcription regulation |
| 85 | Histone-lysine N-methyltransferase trithorax | 10 | 36 (30) | P20659 | Transcription regulation |
| 86 | Putative gustatory receptor 22c | 2 | 33 (30) | P58952 | G-protein coupled receptor protein signaling pathway |
| 87 | Sterile alpha and TIR motif-containing protein 1 | 2 | 42 (30) | Q6IDD9 | Innate immunity |
| 88 | WD repeat-containing protein 55 homolog | 7 | 39 (30) | Q8T088 | WD repeat WDR55 family |
| 89 | Protein three rows | 9 | 39 (26) | Q6V3V8 | Malpighian tubule morphogenesis |
| 90 | Putative ribosomal RNA methyltransferase CG5220 | 7 | 33 (30) | Q9VEP1 | rRNA processing |
| 91 | Spectrin alpha chain | 2 | 31 (30) | P13395 | Cell shape |
| 92 | V-type proton ATPase subunit d 1 | 4 | 40 (30) | Q9W4P5 | Hydrogen ion transport |
| 93 | Neuropathy target esterase sws | 2 | 35 (32) | B4JLX2 | Neurogenesis |
| 94 | Furin-like protease 1, isoforms 1/1-X/2 | 3 | 32 (32) | P26016 | Proteolysis |
| 95 | RNA helicase armi | 6 | 32 (26) | Q6J5K9 | RNA-mediated gene silencing |
| 96 | Serine proteinase stubble | 4 | 40 (29) | Q05319 | Actin filament bundle assembly |
| 97 | La-related protein | 6 | 38 (29) | Q9VAW5 | Mitochondrion inheritance |
| 98 | Protein abrupt | 3 | 33 (29) | Q24174 | Transcription regulation |
| 99 | Protein sevenless | 5 | 45 (30) | P13368 | Sensory transduction |
| 100 | RING finger protein unkempt | 5 | 43 (30) | Q86B79 | Essential for late larval and early pupal development |
| 101 | Retrovirus-related Pol polyprotein from type-2 retrotransposable element R2DM | 5 | 41 (30) | P16423 | RNA-dependent DNA replication |
| 102 | Angiotensin-converting enzyme-related protein | 4 | 39 (30) | Q9VLJ6 | Heart development/specific maturation |
| 103 | Spectrin beta chain | 8 | 38 (30) | Q00963 | Actin filament capping |
| 104 | Protein flightless-1 (*Flil*) | 5 | 36 (30) | Q24020 | Flight behavior/structural role in indirect flight muscle |
| 105 | Protein slit | 4 | 32 (30) | P24014 | Differentiation/Neurogenesis |
| 106 | 40S ribosomal protein S23 | 6 | 31 (30) | Q8T3U2 | Translation |
| 107 | E3 ubiquitin-protein ligase Su(dx) | 5 | 37 (29) | Q9Y0H4 | Notch signaling pathway/Ubl conjugation pathway |
| 108 | Tyrosine-protein kinase transmembrane receptor Ror | 3 | 31(30) | Q24488 | Central nervous system development |
| 109 | Collagen alpha-1(IV) chain | 4 | 33 (29) | P08120 | Dorsal closure |
| 110 | Nuclear pore complex protein Nup88 | 4 | 31 (29) | Q9GYU8 | Immunity/Antimicrobial humoral response |
| 111 | Retrovirus-related Gag polyprotein from transposon HMS-Beagle | 4 | 30 (29) | Q967S7 | Strongly basic protein |
| 112 | General transcription factor IIF subunit 1 | 5 | 44 (29) | Q05913 | Positive regulation of transcription |
| 113 | Mitosis initiation protein fs(1)Ya | 2 | 40 (29) | P25028 | Cell cycle/Cell division/Mitosis |
| 114 | E3 ubiquitin-protein ligase highwire | 8 | 52 (26) | Q9NB71 | Ubl conjugation pathway |
| 115 | Trehalase | 3 | 30 (29) | Q9W2M2 | Trehalose metabolic process |
| 116 | Serine hydrolase | 3 | 39 (29) | O18391 | Detoxification/Digestion |
| 117 | Protein snail | 3 | 37 (29) | P08044 | Essential for the correct specification of ventral-dorsal patterns |
| 118 | Protein male-specific lethal-3 | 3 | 36 (29) | P50536 | Chromatin assembly or disassembly |
| 119 | T-complex protein 1 subunit gamma | 3 | 33 (29) | P48605 | Mitotic spindle organization |
| 120 | Cleavage and polyadenylation specificity factor subunit1 | 2 | 30 (29) | Q9V726 | mRNA processing |
| 121 | Uncharacterized protein CG42248 | 3 | 30 (29) | Q9W5D0 | Phosphoprotein |
| 122 | Endoribonuclease Dcr-1 | 3 | 30 (28) | Q9VCU9 | RNA-mediated gene silencing |
| 123 | DNA mismatch repair protein spellchecker 1 | 3 | 30 (29) | P43248 | DNA damage/DNA repair |
| 124 | HEAT repeat-containing protein 1 homolog | 12 | 29 (28) | Q9VM75 | Ribosome biogenesis/rRNA processing |
| 125 | Larval serum protein 1 beta chain | 3 | 29 (28) | P11996 | A store of amino acids for synthesis of adult proteins |
| 126 | Centrosomin | 3 | 47 (32) | P54623 | Central nervous system development |
| 127 | Retrovirus-related Pol polyprotein from transposon 297 | 11 | 30 (28) | P20825 | Aspartyl protease |
| 128 | Dystrophin, isoform E | 10 | 38 (36) | Q7YU29 | Specifically required for survival and integrity of the larval musculature |
| 129 | Protein tamozhennic | 3 | 32 (29) | Q9W1A4 | Multicellular organismal development |
| 130 | Pol polyprotein | 12 | 41 (36) | O76326 | DNA integration |
| 131 | Fructose-bisphosphate aldolase | 3 | 159 (40) | P07764 | Glycolysis |
| 132 | Actin, larval muscle | 3 | 119 (40) | P02574 | Actins are highly conserved proteins that are involved in various types of cell motility and are ubiquitously expressed in all eukaryotic cells. |
| 133 | Glyceraldehyde-3-phosphate dehydrogenase 2 | 2 | 79 (40) | P07487 | Glycolysis |
| 134 | CG7289 | 2 | 66 (40) | Q9VQ60 | Not known |
| 135 | CG3699 | 3 | 42 (40) | Q9U1L2 | Oxidation reduction |
| 136 | LD22412p | 2 | 58 (40) | Q8T078 | Dendrite morphogenesis |
| 137 | Fcp1/CTD (carboxy-terminal domain) | 2 | 54 (40) | Q9W147 | Hydrolase |
| 138 | CG17255, isoform A | 2 | 54 (40) | Q9W2U7 | Entrainment of circadian clock |
| 139 | Histone-lysine N-methyltransferase ash1 | 3 | 53 (40) | Q8MQX5 | Chromatin-mediated maintenance of transcription |
| 140 | Ubiquitin-protein ligase | 2 | 51 (40) | Q9NGB1 | Ubl conjugation pathway |
| 141 | Leucine-rich repeat-containing G protein-coupled receptor 2 | 6 | 36 (27) | Q9BN18 | G-protein coupled receptor signaling |
| 142 | Protein spint | 3 | 29 (26) | Q8MQW8 | Developmental protein, GTPase activation |
| 143 | Strn-Mlck | 6 | 45 (30) | A1ZA72 | Protein amino acid phosphorylation |
| 144 | Pollux, isoform A | 2 | 48 (40) | Q9VNG9 | Cell adhesion mediated by integrin/Regulation of Rab GTPase activity |
| 145 | H/ACA ribonucleoprotein complex | 3 | 41 (40) | Q7KVQ0 | Ribosome biogenesis/rRNA processing |
| 146 | Kinesin-like protein Klp10A | 3 | 44 (40) | Q960Z0 | Cell cycle |
| 147 | Flap endonuclease GEN | 7 | 42 (36) | Q9U9Q6 | DNA catabolic process, endonucleolytic |
| 148 | Nesprin | 3 | 37 (36) | Q71JA7 | Actin filament organization |
| 149 | Antimeros | 6 | 38 (36) | Q9VN55 | Protein binding |
| 150 | Leucine-rich repeat protein soc-2 | 3 | 44 (33) | B5DX45 | Leucine-rich repeat |
| 151 | Metallothionein-1 | 3 | 58 (36) | P61873 | Detoxification |
| 152 | Protein timeless | 4 | 37 (36) | O17482 | Biological rhythms |
| 153 | DNA topoisomerase 2 | 4 | 44 (26) | P15348 | DNA topological change |
| 154 | Putative vitellogenin receptor | 7 | 41 (26) | P98163 | Endocytosis |
| 155 | Laminin subunit beta-1 | 4 | 40 (26) | P11046 | Cell adhesion |
| 156 | Protein expanded | 3 | 39 (26) | Q07436 | Transcription regulation |
| 157 | ATP-dependent RNA helicase vasa | 7 | 38 (26) | P09052 | Differentiation/ Oogenesis |
| 158 | Cytochrome P450 4g1 | 5 | 38 (26) | Q9V3S0 | Lipid metabolic process |
| 159 | ATP-dependent RNA helicase p62 | 4 | 36 (26) | P19109 | RNA-mediated gene silencing |
| 160 | Defective chorion-1 protein, FC177 | 8 | 34 (26) | P18171 | Eggshell chorion assembly |
| 161 | Ubiquitin carboxyl-terminal hydrolase 64E | 6 | 34 (26) | Q24574 | Ubl conjugation pathway |
| 162 | Serine/threonine-protein kinase Smg1 | 4 | 34 (26) | Q70PP2 | Nonsense-mediated mRNA decay |
| 163 | CAD protein | 4 | 33 (26) | P05990 | Pyrimidine biosynthesis |
| 164 | Neural-cadherin | 4 | 32 (26) | O15943 | Cell adhesion |
| 165 | Conserved oligomeric Golgi complex subunit 4 | 3 | 32 (26) | Q95TN4 | Protein transport |
| 166 | Gametogenetin-binding protein 2-like | 5 | 32 (26) | Q9VNG1 | Not known |
| 167 | DNA replication licensing factor Mcm6 | 3 | 32 (26) | Q29JI9 | Cell cycle |
| 168 | Lysine-specific demethylase 4B | 4 | 32 (26) | Q9V6L0 | Histone H3-K36 demethylation |
| 169 | Trifunctional purine biosynthetic protein adenosine-3 | 4 | 31 (26) | P00967 | Purine biosynthesis |
| 170 | Protein bcn92 | 3 | 31 (26) | P82116 | Not Known |
| 171 | Supporter of activation of yellow protein | 5 | 30 (26) | Q9VWF2 | Negative regulation of transcription, DNA-dependent |
| 172 | Homeobox protein prospero | 5 | 30 (26) | Q9U6A1 | Multicellular organismal development |
| 173 | Prominin-like protein | 2 | 29 (26) | P82295 | Not known |
| 174 | Lysozyme P | 3 | 28 (26) | P29615 | Cell wall macromolecule catabolic process |
| 175 | Muscle LIM protein Mlp84B | 2 | 28 (26) | Q24400 | Muscle organ development |
| 176 | Putative U5 small nuclear ribonucleoprotein 200 kDa helicase | 4 | 28 (26) | Q9VUV9 | mRNA processing |
| 177 | Ubiquitin-like protein 5 | 2 | 27 (26) | Q9V998 | Ubl conjugation pathway |
| 178 | Protein nullo | 3 | 27 (26) | P32845 | Cell-cell junction assembly |
| 179 | N6-adenosine-methyltransferase MT-A70-like protein | 3 | 27 (26) | Q9VCE6 | RNA methylation |
| 180 | Cytochrome P450 4ad1 | 2 | 27 (26) | Q9V4T3 | Oxidation reduction |
| 181 | Tyrosine-protein kinase PR2 | 6 | 27 (26) | Q9I7F7 | Protein amino acid phosphorylation |
| 182 | Eye-specific diacylglycerol kinase | 4 | 28 (26) | Q09103 | Phospholipid turnover within the photoreceptor |
| 183 | Opsin Rh6 | 2 | 27 (26) | O01668 | Sensory transduction |
| 184 | Paramyosin | 5 | 29 (25) | P35415 | Major structural component of many thick filaments isolated from invertebrate muscles |
| 185 | Alpha-(1,3)-fucosyltransferase C | 8 | 27 (26) | P83088 | Protein amino acid glycosylation |
| 186 | RNA-binding protein cabeza | 4 | 71 (36) | Q27294 | Nuclear mRNA splicing, via spliceosome |
| 187 | Transcription factor grauzone | 3 | 30 (28) | Q9U405 | Meiosis |
| 188 | Calbindin-32 | 3 | 30 (28) | P41044 | Calcium ion binding |
| 189 | rRNA 2'-O-methyltransferase fibrillarin | 3 | 30 (28) | Q9W1V3 | rRNA processing |
| 190 | Phenylalanyl-tRNA synthetase | 4 | 30 (28) | O16129 | Protein biosynthesis |
| 191 | Slowpoke-binding protein | 5 | 60 (32) | Q8IPH9 | Regulator of calcium channel/regulation of synaptic transmission |
| 192 | Insuline-like receptor | 18 | 80 (26) | P09208 | Differentiation/Growth regulation/Neurogenesis |
| 193 | Regulator of nonsense transcripts 1 homolog | 8 | 67 (26) | Q9CVYS3 | Nuclear-transcribed mRNA catabolic process, nonsense-mediated decay |
| 194 | Nucleic-acid-binding protein from mobile element jocker | 6 | 60 (26) | P21330 | Nucleic acid binding |
| 195 | Protein decapentaplegic | 8 | 54 (26) | P07713 | BMP (bone morphogenetic protein) signaling pathway |
| 196 | Vesicular-fusion ATPase 1 | 5 | 47 (26) | P46461 | ER-Golgi transport |
| 197 | Protein crumbs | 5 | 45 926) | P10040 | Differentiation |
| 198 | Cytochrome-P450 4p1 | 3 | 41 (26) | Q9V558 | Oxidation reduction |
| 199 | VPRBP-like protein | 4 | 51 (33) | Q9W2F2 | Phosphoprotein |
| 200 | Elongation factor 1-alpha | 2 | 27 (25) | P27592 | Protein biosynthesis *Onchocerca volvulus* |
| 201 | signal recognition particle 72 kDa protein | 2 | 32 (25) | P49965 | SRP-dependent cotranslational protein targeting to membrane *Schistosoma mansoni* (Blood fluke) |
| 202 | Sex-determining transformer protein 2 | 3 | 33 (24) | Q9NIW4 | Sexual differentiation *Caenorhabditis Remanei (Caenorhabditis vulgaris)* |
| 203 | Mite group 2 allergen Der f 2 | 2 | 28 (24) | Q00855 | Extracellular region *Dermatophagoides Farinae (American house dust mite)* |
| 204 | Tropomyosin | 3 | 27 (24) | O02389 | Central role in the calcium dependent regulation of muscle contraction *Chlamys nipponensis akazara* (Akazara scallop) (Japanese scallop) |
| 205 | Guanine nucleotide-binding protein G(s) subunit alpha | 3 | 28 (25) | P30669 | G-protein coupled receptor protein signaling pathway *Schistosoma mansoni*(Blood fluke) |
| 206 | Toxin Aah6 | 2 | 27 (25) | P56743 | Defense response pathogenesis *Androctonus australis* (Sahara scorpion) |
| 207 | G2/mitotic-specific cyclin-B | 9 | 31 (24) | P18063 | Cell cycle *Asterina pectinifera* |
| 208 | Guanine nucleotide-binding protein alpha-17 subunit | 3 | 27 (25) | Q86FX7 | Chemotaxi/G-protein coupled receptor protein signaling pathway *Caenorhabditis briggsae* |
| 209 | Hemocyanin subunit B | 2 | 26 (25) | Q8IFJ8 | Oxygen transport *Scutigera coleoptrata* (House centipede) |
| 210 | 227 kDa spindle- and centromere-associated protein | 6 | 35 (24) | O61308 | Cell cycle *Parascaris univalens* |
| 211 | Extracellular matrix protein 3 | 9 | 33 (24) | Q9GV77 | Cell adhesion *Lytechinus variegatus* |
| 212 | Annexin-B12 | 5 | 32 (24) | P26256 | Calcium-dependent phospholipid binding *Hydra attenuata* |
| 213 | Vitellogenin-1 | 3 | 26 (25) | Q9U8M0 | Lipid transport *Periplaneta Americana (American cockroach) (Blatta americana)* |
| 214 | Arginine kinase | 2 | 30 (25) | P51541 | Phosphorylation *Limulus polyphemus (Atlantic horseshoe crab)* |
| 215 | Syntaxin | 3 | 35 (25) | Q16932 | Neurotransmitter transport *Aplysia californica* (California sea hare) |
| 216 | Armadillo segment polarity protein | 2 | 27 (24) | Q7QHW5 | Cell adhesion/Wnt signaling pathway *Anopheles gambiae* (African malaria mosquito) |
| 217 | Toxin BeM14 | 2 | 26 (24) | P09982 | Defense response *Buthus eupeus* |
| 218 | Cathepsin B-like cysteine proteinase | 2 | 29 (25) | P43157 | Proteolysis *Schistosoma japonicum* (Blood fluke) |
| 219 | Ring canal kelch homolog | 3 | 26 (25) | Q70JS2 | Ring canal kelch homolog *Anopheles Stephensi* (Indo-Pakistan malaria mosquito) |
| 220 | Venom allergen 5.01 | 3 | 26 (24) | P35781 | Secreted *Vespa crabro* |
| 221 | DNA-directed RNA polymerase II subunit RPB1 | 3 | 29 (25) | P35074 | Transcription from RNA polymerase II promoter *Caenorhabditis briggsae* |
| 223 | Fasciclin-2 | 5 | 29 (25) | P22648 | Cell adhesion *Schistocerca americana* (American grasshopper) |
| 224 | Histone H1, early embryonic | 3 | 26 (25) | P19375 | Nucleosome assembly *Strongylocentrotus purpuratus* (Purple sea urchin) |
| 225 | Antichymotrypsin-1 | 5 | 27 (24) | Q03383 | Inhibits chymotrypsin activity *Bombyx mori* (silk moth) |
| 226 | Potassium channel toxin TsTXK-beta | 3 | 33 (26) | P69940 | Pathogenesis *Tityus serrulatus* (Brazilian scorpion) |
| 227 | Metallothionein 20-I isoforms A and B | 6 | 29 (26) | P80251 | Cellular sequestration of toxic metal ions Mytilus edulis |
| 228 | Nitrophorin-1 | 2 | 28 (24) | Q26239 | Vasodilation *Rhodnius prolixus* |
| 229 | 60S acidic ribosomal protein P0 | 8 | 28 (26) | Q9U3U0 | Ribosome biogenesis *Ceratitis capitata* (Mediterranean fruit fly) (Tephritis capitata) |
| 230 | Trifunctional purine biosynthetic protein adenosine-3 | 2 | 27 (26) | Q26255 | Purine biosynthesis *Chironomus tentans* |
| 231 | Triosephosphate isomerase | 4 | 27 (26) | Q9GTX8 | Fatty acid biosynthesis *Taenia solium* |
| 232 | 60S ribosomal protein L13 | 3 | 27 (26) | O46157 | Ribonucleoprotein *Lumbricus rubellus* |
| 233 | Potassium channel toxin alpha-KTx 2.6 | 5 | 33 (25) | P59849 | Pathogenesis *Centruroides limbatus* |
| 234 | Metallothionein-B | 10 | 27 (26) | Q26496 | Metal ion binding *Sphaerechinus granularis* |
| 235 | Acetylcholine receptor subunit alpha-L1 | 4 | 31 (25) | P23414 | Ion transport *Schistocerca gregaria* (Desert locust) |
| 236 | 78 kDa glucose-regulated protein | 2 | 32 (25) | Q16956 | Assembly of multimeric protein complexes inside the ER *Aplysia californica* (California sea hare) |
| 237 | Metallothionein | 6 | 29 (25) | O02033 | Metal ion binding *Lytechinus pictus* (Painted sea urchin) |
| 238 | Stress-activated protein kinase JNK-1 | 2 | 29 (25) | Q9U6D2 | JNK cascade *Ancylostoma caninum* (Dog hookworm) |
| 239 | Adhesive plaque matrix protein | 2 | 27 (25) | Q25460 | Adhesiveness to the mussel's foot *Mytilus edulis* (Blue mussel) |
