# Supplementary material for: Diet-Induced Over-Expression of Flightless-I Protein and Its Relation to Flightlessness in Mediterranean Fruit Fly, Ceratitis capitata
Source: PLoS One. 2013 Dec 3;8(12):e81099. doi: 10.1371/journal.pone.0081099 (PMC3849048; doi:10.1371/journal.pone.0081099)
Supplement: Table S4 — A list of 233 under-expressed proteins in pupae B whose adult flies showed a low flight rate. (DOC) [file pone.0081099.s004.doc]

**Supporting Information (SI)**

**Diet-induced over-expression of flightless-I protein and its relation to flightlessness in Mediterranean fruit fly, *Ceratitis capitata***

Il Kyu Cho^1^, Chiou Ling Chang^2^ and Qing X. Li^1^*

^1^ Department of Molecular Biosciences and Bioengineering, University of Hawaii, Honolulu, Hawaii, USA.

^2^ U.S. Pacific Basin Agricultural Research Center, Hilo, Hawaii, USA.

**Table S4** **A list of 233 under-expressed proteins in pupae B whose adult flies showed a low flight rate.** The LC-MS/MS data were matched with *Drosophila melanogaster* database via MASCOT for the sequence alignments.

| No. | Protein names | No. of matched peptides | Mascot Scores (p=0.05) | Accession numbers | Biological functions |
| --- | --- | --- | --- | --- | --- |
| 1 | Bifunctional arginine demethylase and lysyl-hydroxylase PSR | 5 | 70 (33) | Q9VD28 | Dioxygenase |
| 2 | Neuropathy target esterase sws | 5 | 62 (33) | B3NY03 | Neurogenesis/Endoplasmic reticulum |
| 3 | Interference hedgehog | 5 | 61 (33) | B4GKZ8 | Smoothened signaling pathway |
| 4 | Protein split ends | 9 | 63 (26) | Q8SX83 | Wnt receptor signaling pathway |
| 5 | Serine/threonine-protein kinase PLK4 | 4 | 57 (33) | O97143 | Centriole replication |
| 6 | Histone H1 | 8 | 54 (33) | P02255 | Nucleosome assembly |
| 7 | Protein KIAA0664 | 4 | 53 (33) | B4GAM1 | KIAA0664/TIF31 family.c |
| 8 | G protein-coupled receptor kinase 1 | 4 | 35 (29) | P32865 | Specifically phosphorylates the activated forms of G protein-coupled receptors |
| 9 | Protein cubitus interruptus | 4 | 51 (33) | P19538 | Cuticle pattern formation |
| 10 | Protein lin-54 | 5 | 49 (33) | A1Z9E2 | Adult lifespan |
| 11 | Protein daughter of sevenless | 3 | 47 (33) | Q9VZZ9 | signaling from various receptor tyrosine kinases such as Sevenless |
| 12 | Protein disks lost | 2 | 46 (33) | Q8T626 | Cellular process |
| 13 | RNA-binding protein orb2 | 3 | 46 (33) | Q9VSR3 | Long-term memory |
| 14 | Axin | 5 | 46 (33) | Q9V407 | Wnt signaling pathway |
| 15 | Eukaryotic translation initiation factor 3 subunit B | 3 | 54 (33) | B4J6D5 | Protein biosynthesis |
| 16 | Eukaryotic translation initiation factor 3 subunit D-2 | 5 | 44 (33) | B4QT07 | Protein biosynthesis |
| 17 | Origin recognition complex subunit 2 | 4 | 44 (33) | Q24168 | DNA replication/nucleus |
| 18 | Tyrosine-protein phosphatase corkscrew | 3 | 44 (33) | Q24708 | Protein amino acid dephosphorylation |
| 19 | Myosin heavy chain, non-muscle | 6 | 43 (33) | Q99323 | Malpighian tubule morphogenesis |
| 20 | Negative elongation factor E | 2 | 43 (33) | Q95ZE9 | Transcription regulation/Nucleus |
| 21 | DNA polymerase subunit gamma-1 | 4 | 42 (33) | Q27607 | DNA replication |
| 22 | Histone-lysine N-methyltransferase, H3 lysine-79 specific | 5 | 54 (33) | Q8INR6 | Chromatin silencing at telomere |
| 23 | Cell division cycle 2-like protein kinase CG7597 | 4 | 41 (33) | Q9VP22 | Protein amino acid phosphorylation |
| 24 | Diacylglycerol kinase 1 | 5 | 41 (33) | Q01583 | Activation of protein kinase C activity by G-protein coupled receptor protein signaling pathway |
| 25 | Nucleolar protein 14 | 3 | 40 (33) | Q9VEJ2 | Ribosome biogenesis/rRNA processing |
| 26 | Protein sickie | 8 | 73 (33) | Q9VIQ9 | Immune response/Innate immunity |
| 27 | Histone-lysine N-methyltransferase Mes-4 | 4 | 39 (33) | Q8MT36 | Histone methyltransferase |
| 28 | Inhibitor of nuclear factor kappa-B kinase | 2 | 39 (33) | Q9VEZ5 | Immune response/Innate immunity |
| 29 | Tyrosine-protein phosphatase corkscrew | 3 | 39 (33) | P29349 | Epidermal growth factor receptor signaling pathway |
| 30 | La-related protein CG11505 | 5 | 39 (33) | Q9I7T7 | RNA binding |
| 31 | Conserved oligomeric Golgi complex | 3 | 38 (33) | Q9VJD3 | Differentiation |
| 32 | Dual specificity mitogen-activated protein kinase kinase hemipterous | 2 | 38 (33) | Q23977 | JNK (c-Jun N-terminal kinase) cascade |
| 33 | Centaurin-gamma-1A | 12 | 37 (33) | Q9NGC3 | Regulation of ARF GTPase activity |
| 34 | Protein aurora borealis | 2 | 37 (33) | Q9VVR2 | Cell cycle |
| 35 | Glutamate dehydrogenase | 2 | 37 (33) | P54385 | NADH oxidation |
| 36 | Circadian locomoter output cycles protein kaput | 2 | 36 (33) | O61735 | Behavioral response to cocaine |
| 37 | Homeotic protein female sterile | 4 | 36 (33) | P13709 | Multicellular organismal development |
| 38 | Kinesin heavy chain | 2 | 36 (33) | P17210 | Axon cargo transport/microtubule |
| 39 | Eukaryotic translation initiation factor 3 | 5 | 35 (33) | B3LZN3 | Protein biosynthesis |
| 40 | Protein spire | 3 | 35 (33) | Q29KT5 | Multicellular organismal development |
| 41 | Polycomb protein Su(z)12 | 4 | 40 (33) | Q9NJG9 | Dendrite morphogenesis |
| 42 | RRP12-like protein | 4 | 38 (33) | Q9VYA7 | Phosphoprotein |
| 43 | Cyclic AMP response element-binding protein A | 2 | 34 (33) | P29747 | Chitin-based larval cuticle pattern formation |
| 44 | Transcription-associated protein 1 | 5 | 42 (33) | Q8I8U7 | Transcription regulation |
| 45 | Host cell factor | 2 | 34 (33) | Q9V4C8 | Cell cycle |
| 46 | Serrate RNA effector molecule | 3 | 34 (33) | Q9V9K7 | RNA-mediated gene silencing |
| 47 | Histone-lysine N-methyltransferase ash1 | 7 | 70 (33) | Q9VW15 | Chromatin-mediated maintenance of transcription |
| 48 | Exocyst complex component 6 | 5 | 67 (33) | Q9VDE6 | Exocytosis/cytoplasmic vesicle |
| 49 | Protein three rows | 5 | 61 (33) | P42286 | Cell cycle |
| 50 | Integrin alpha-PS4 | 7 | 56 (33) | Q9V7A4 | Cell adhesion |
| 51 | Exostosin-1 (Protein tout-velu) | 9 | 54 (33) | Q9V730 | Wnt signaling pathway |
| 52 | Cell cycle regulator Mat89Bb | 4 | 52 (33) | B4QX59 | Cell cycle/regulation of mitotic cell cycle |
| 53 | Poly(A) RNA polymerase gld-2 homolog B | 6 | 52 (33) | Q9VYS4 | mRNA processing |
| 54 | Spastin | 5 | 51 (33) | B4G437 | Cell cycle |
| 55 | Serine protease nudel | 6 | 51 (33) | P98159 | Toll signaling pathway |
| 56 | FACT complex subunit spt16 | 5 | 51 (33) | Q8IRG6 | Transcription regulation |
| 57 | Protein lava lamp | 13 | 35 (33) | Q8MSS1 | Cellularization/Golgi apparatus |
| 58 | Cyclin-T | 4 | 49 (33) | O96433 | Actin filament organization |
| 59 | Dual specificity tyrosine-phosphorylation-regulated kinase 2 | 5 | 49 (33) | Q9V3D5 | Olfactory behavior |
| 60 | Lysine-specific demethylase lid | 5 | 48 (33) | Q9VMJ7 | Histone H3-K4 demethylation |
| 61 | Lateral signaling target protein | 3 | 47 (33) | B4IC49 | Negative regulator of epidermal growth factor receptor (EGFR) signaling |
| 62 | Myosin heavy chain 95F | 5 | 46 (33) | Q01989 | Actin cytoskeleton organization |
| 63 | Protein suppressor of variegation 3-7 | 2 | 33 (32) | P20193 | Dose-limiting factor in position-effect variegation |
| 64 | Proteasome-associated protein ECM29 | 7 | 70 (26) | Q9V677 | Protein catabolic process |
| 65 | Uncharacterized protein CG31531 | 5 | 44 (33) | Q0KIC3 | Not known |
| 66 | Glutamate [NMDA] receptor | 3 | 43 (33) | B4KD90 | Ion transport |
| 67 | Caprin | 11 | 42 (33) | Q9I7D3 | Monolayer-surrounded lipid storage body |
| 68 | Putative N(4)-(beta-N-acetylglucosaminyl)-L-asparaginase GL17147 | 3 | 42 (33) | B4GGF2 | Protein deglycosylation |
| 69 | Homeobox protein extradenticle | 3 | 42 (33) | P40427 | Transcription |
| 70 | Kinesin-like protein CG14535 | 6 | 42 (33) | Q9VLW2 | Microtubule-based movement |
| 71 | GDP-fucose transporter | 2 | 41 (33) | Q9VHT4 | Sugar transport/Golgi apparatus |
| 72 | Pituitary homeobox 1 | 12 | 41 (33) | O18400 | Transcription |
| 73 | Protein furry | 7 | 40 (33) | Q9VT28 | Transcription regulation/Phosphoprotein |
| 74 | Gamma-aminobutyric acid receptor alpha-like | 4 | 40 (33) | Q24352 | Ion transport |
| 75 | Sodium- and chloride-dependent GABA transporter ine | 3 | 39 (33) | Q9VR07 | Differentiation/Neurogenesis |
| 76 | Protein tramtrack, alpha isoform | 6 | 30 (29) | P42282 | Transcription regulation |
| 77 | Protein eyes shut | 13 | 38 (33) | A0A1F4 | Rhabdomere development |
| 78 | Molybdenum cofactor sulfurase | 12 | 38 (33) | B4JXP7 | Molybdenum cofactor biosynthesis |
| 79 | GDP-mannose 4,6 dehydratase | 4 | 38 (33) | Q9VMW9 | GDP-L-fucose biosynthetic process/GDP-mannose metabolic process/intracellular |
| 80 | Succinate dehydrogenase [ubiquinone] flavoprotein | 11 | 43 (33) | Q94523 | Electron transport |
| 81 | Protein hook | 6 | 37 (33) | B4G831 | Endocytosis |
| 82 | Protein disabled | 5 | 37 (33) | P98081 | Differentiation/Neurogenesis |
| 83 | Putative fat-like cadherin-related tumor suppressor | 6 | 37 (33) | Q9VW71 | Cell adhesion |
| 84 | Zinc finger protein 2 | 4 | 37 (33) | P28167 | Imaginal disc-derived wing morphogenesis |
| 85 | Furin-like protease 1, isoform 1-CRR | 3 | 37 (33) | P30430 | Proteolysis/integral to membrane |
| 86 | E3 ubiquitin-protein ligase mind-bomb | 3 | 37 (33) | Q9VUX2 | Notch signaling pathway/Ubl conjugation pathway |
| 87 | Tripeptidyl-peptidase 2 | 2 | 36 (33) | Q9V6K1 | Protein homooligomerization |
| 88 | Formin-like protein CG32138 | 2 | 36 (33) | Q9VUC6 | Actin cytoskeleton organization |
| 89 | Protein SMG8 | 3 | 36 (33) | B4GH42 | Nonsense-mediated mRNA decay |
| 90 | Cytochrome P450 28d2 | 2 | 36 (33) | Q9VMT6 | Oxidation reduction |
| 91 | Transcription initiation factor TFIID | 13 | 36 (33) | Q9VWY6 | Regulation of transcription |
| 92 | PP2C-like domain-containing protein CG9801 | 6 | 52 (33) | Q0KIA2 | Not known |
| 93 | Putative dual specificity tyrosine-phosphorylation-regulated kinase 3 | 3 | 35 (33) | P83102 | Protein amino acid phosphorylation |
| 94 | Serine/threonine-protein kinase grp | 5 | 35 (29) | O61661 | Cell cycle |
| 95 | Poly [ADP-ribose] polymerase | 3 | 41 (33) | P35875 | Chromatin modification |
| 96 | Eukaryotic translation initiation factor 3 | 4 | 34 (33) | B4GDX4 | Protein biosynthesis |
| 97 | Protein MCM10 | 6 | 34 (33) | Q9VIE6 | DNA replication |
| 98 | Serine-enriched protein | 5 | 34 (33) | O61366 | Not known |
| 99 | Semaphorin-1A | 4 | 33 (29) | Q24322 | Differentiation/Neurogenesis (a role in growth cones guidance) |
| 100 | Protein purity of essence | 5 | 52 (33) | Q9VLT5 | Differentiation/Neurogenesis |
| 101 | Protein rigor mortis | 4 | 48 (33) | Q86BY9 | Transcription regulation |
| 102 | Protocadherin-like wing polarity protein stan | 5 | 45 (33) | Q9V5N8 | Cell adhesion/Cell membrane |
| 103 | Bipolar kinesin KRP-130 | 2 | 43 (33) | P46863 | Cell cycle/Microtubule |
| 104 | Protein BCL9 | 5 | 42 (33) | Q961D9 | Wnt signaling pathway |
| 105 | FACT complex subunit Ssrp1 | 4 | 42 (33) | Q05344 | DNA damage/DNA repair |
| 106 | Protein male-specific lethal-2 | 2 | 36 (33) | P50534 | Dosage compensation complex assembly |
| 107 | H/ACA ribonucleoprotein complex non-core subunit NAF1 | 2 | 41 (33) | Q9VJ62 | Ribosome biogenesis/rRNA processing |
| 108 | Methionyl-tRNA synthetase | 2 | 39 (33) | Q9VFL5 | Protein biosynthesis |
| 109 | Phosphorylase b kinase regulatory | 3 | 38 (33) | Q9VLS1 | Carbohydrate/Glycogen metabolism |
| 110 | DNA repair protein RAD50 | 2 | 37 (33) | Q9W252 | Cell cycle/Chromosomal protein/ |
| 111 | DNA-binding protein modulo | 3 | 36 (33) | P13469 | Cell proliferation |
| 112 | Putative gustatory receptor 98a | 4 | 36 (33) | Q9VB30 | G-protein coupled receptor protein signaling pathway |
| 113 | Cadherin-86C | 3 | 36 (33) | Q9VGW1 | Cell adhesion |
| 114 | Kinesin-like protein GA13060 | 3 | 35 (33) | Q29MB2 | Microtubule-based movement |
| 115 | ATP synthase subunit beta | 3 | 35 (33) | Q05825 | ATP synthesis coupled proton transport |
| 116 | Putative tyrosine-protein kinase Wsck | 4 | 35 (33) | P83097 | Protein amino acid phosphorylation |
| 117 | Bifunctional heparan sulfate N-deacetylase/N-sulfotransferase | 2 | 35 (33) | Q9V3L1 | Wnt signaling pathway |
| 118 | Transmembrane GTPase Marf | 3 | 34 (33) | Q7YU24 | Mitochondrial fusion |
| 119 | cAMP-dependent protein kinase catalytic | 2 | 34 (33) | P12370 | Anterior/posterior pattern formation, imaginal disc/plasma membrane |
| 120 | Hsp90 co-chaperone Cdc37 | 4 | 30 (29) | Q24276 | Cell cycle |
| 121 | Yemanuclein-alpha | 2 | 34 (32) | P25992 | Egg organization/transcriptional regulator |
| 122 | Claspin | 3 | 45 (32) | Q8IRB5 | Phosphoprotein |
| 123 | Diacylglycerol kinase eta | 5 | 44 (32) | B4JHJ7 | Activation of protein kinase C activity by G-protein coupled receptor protein signaling pathway |
| 124 | Protein CLP1 | 2 | 40 (32) | B4HQJ2 | mRNA processing |
| 125 | F-box/WD repeat-containing protein 7 | 3 | 39 (32) | Q9VZF4 | Cell cycle/Ubl conjugation pathway |
| 126 | Actin-binding protein anillin | 2 | 39 (32) | Q9V4P1 | Cell cycle |
| 127 | Protein single-minded | 2 | 37 (32) | P05709 | Differentiation/Neurogenesis |
| 128 | Uncharacterized protein CG4951 | 2 | 37 (32) | A1A708 | Phosphoprotein |
| 129 | Puff-specific protein Bx42 | 2 | 37 (32) | P39736 | Embryonic development via the syncytial blastoderm/eye-antennal disc development |
| 130 | L-asparaginase CG7860 | 2 | 36 (32) | Q9VXT7 | Asparagine catabolic process via L-aspartate |
| 131 | Broad-complex core protein | 4 | 35 (32) | Q24206 | Autophagy |
| 132 | Serine protease HTRA2 | 3 | 35 (32) | B4K835 | Apoptosis |
| 133 | Protein scabrous | 2 | 34 (32) | P21520 | Notch signaling pathway/extracellular space |
| 134 | Fragile X mental retardation syndrome-related protein 1 | 4 | 33 (29) | Q9NFU0 | RNA-mediated gene silencing |
| 135 | SH3 domain-binding protein 5 | 3 | 45 (29) | Q9V785 | Anterior/posterior pattern formation |
| 136 | Cytochrome P450 4aa1 | 2 | 32 (29) | Q9V7G5 | Oxidation reduction (metabolism of insect hormones and breakdown of synthetic insecticides) |
| 137 | DNA replication licensing factor MCM4 | 18 | 30 (29) | Q26454 | Mitotic DNA replication |
| 138 | Myosin-VIIa | 3 | 40 (33) | Q29P71 | Actin filament-based movement |
| 139 | Bicaudal D-related protein | 5 | 87 (33) | Q8SWR2 | Belongs to the BICDR family |
| 140 | DNA repair protein complementing XP-C cells | 3 | 34 (32) | Q24595 | DNA damage/DNA repair |
| 141 | 26S protease regulatory subunit 4 | 6 | 32 (29) | P48601 | Cell proliferation (ATP-dependent degradation of ubiquitinated proteins) |
| 142 | RuvB-like helicase 2 | 2 | 35 (33) | Q9V3K3 | Wnt receptor signaling pathway |
| 143 | Transcription initiation factor TFIID subunit 1 | 4 | 57 (33) | P51123 | Cell cycle |
| 144 | Regulator of telomere elongation helicase 1 | 4 | 46 (33) | B3MSG8 | DNA damage/DNA repair/Nucleus |
| 145 | Cytochrome P450 309a1 | 19 | 37 (29) | Q9VQD2 | Oxidation reduction |
| 146 | Protein Shroom | 6 | 39 (33) | A1Z9P3 | Cell migration |
| 147 | Queuine tRNA-ribosyltransferase | 2 | 38 (33) | B4PEV9 | Queuosine biosynthesis/tRNA processing |
| 148 | Cytochrome P450 4d14 | 3 | 34 (33) | O46051 | Oxidation reduction |
| 149 | Protein strawberry notch | 5 | 59 (33) | A8JUV0 | Notch signaling pathway |
| 150 | Integrin alpha-PS2 | 7 | 37 (29) | P12080 | Cell adhesion |
| 151 | CWF19-like protein 2 | 2 | 40 (33) | Q9VXT5 | Phosphoprotein |
| 152 | Eukaryotic translation initiation factor 3 subunit C | 2 | 40 (33) | B4MRZ8 | Protein biosynthesis |
| 153 | Rho GTPase-activating protein CG5521 | 2 | 37 (33) | Q9VB98 | Regulation of small GTPase mediated signal transduction |
| 154 | Neural/ectodermal development factor IMP-L2 | 2 | 36 (33) | Q09024 | Cell adhesion/extracellular space |
| 155 | Maternal protein exuperantia-1 | 2 | 35 (33) | Q24618 | Multicellular organismal development |
| 156 | Protein vav | 3 | 47 (33) | Q9NHV9 | Actin filament organization |
| 157 | Receptor-mediated endocytosis protein 6 | 2 | 37 (33) | Q9VZ08 | Endocytosis |
| 158 | Sodium channel protein 60E | 2 | 37 (33) | Q9W0Y8 | Olfactory behavior/voltage-gated sodium channel complex |
| 159 | Longitudinals lacking protein, isoform G | 2 | 36 (33) | P42283 | Differentiation/Neurogenesis |
| 160 | V-type proton ATPase catalytic subunit A | 2 | 34 (33) | P48602 | ATP synthesis coupled proton transport |
| 161 | Glucose-dehydrogenase | 12 | 45 (29) | P18172 | Alcohol metabolic process |
| 162 | SpoIIM-like stage II sporulation protein M related | 4 | 40 (29) | Q9V182 | Not known |
| 163 | Protein O-mannosyl-transferase 2 | 8 | 36 (29) | Q9W5D4 | Lipid glycosylation |
| 164 | Polypeptide N-acetylgalactosaminyl-transferase 3 | 4 | 35 (29) | Q9Y117 | Protein glycosylation |
| 165 | Putative neural-cadherin 2 | 4 | 33 (29) | Q9VJB6 | Calcium dependent cell adhesion proteins |
| 166 | Guanine nucleotide-releasing factor 2 | 7 | 32 (29) | O77086 | Ras protein signal transduction |
| 167 | 120.7 kDa protein in NOF-FB transposable element | 6 | 30 (29) | P16320 | Transposition of NOF-FB and other FB elements |
| 168 | E3 ubiquitin-protein ligase Bre1 | 4 | 43 (29) | Q9VRP9 | Notch signaling pathway/Ubl conjugation pathway |
| 169 | Eukaryotic translation initiation factor 2-alpha kinase | 2 | 30 (29) | Q9NIV1 | Stress response |
| 170 | Protein sevenless | 4 | 35 (30) | P20806 | Sensory transduction/Vision |
| 171 | G1/S-specific cyclin-E | 4 | 44 (29) | P54733 | Essential for the control of the cell cycle at the G1/S (start) transition |
| 172 | Stress-activated map kinase-interacting protein 1 | 7 | 36 (29) | Q9V719 | Apoptosis |
| 173 | Protein ariadne-1 | 15 | 42 (30) | Q94981 | Ubl conjugation pathway |
| 174 | Hepatocyte growth factor-regulated tyrosine kinase substrate | 3 | 40 (29) | Q960X8 | Border follicle cell migration |
| 175 | Restin | 8 | 37 (29) | Q9VJE5 | Cellularization |
| 176 | Protein phosphatase PHLPP-like protein | 2 | 30 (29) | Q9VJ07 | Apoptosis |
| 177 | Laminin subunit gamma-1 | 5 | 38 (29) | P15215 | Cell adhesion |
| 178 | Protein Wnt-5 | 3 | 37 (29) | P28466 | Wnt signaling pathway |
| 179 | Attacin-C | 2 | 30 (29) | Q95NH6 | Innate immunity |
| 180 | Dosage compensation regulator | 28 | 40 (29) | P24785 | Axon extension |
| 181 | ADP, ATP carrier protein | 22 | 30 (29) | Q26365 | Transport |
| 182 | Putative cytoplasmic aminopeptidase | 5 | 38 (29) | Q9V3D8 | Proteolysis |
| 183 | Putative ribosomal RNA methyltransferase CG7009 | 18 | 30 (29) | Q9VDD9 | rRNA processing |
| 184 | Nuclear hormone receptor FTZ-F1 | 7 | 34 (29) | P33244 | Cofactor to fushi tarazu (ftz) |
| 185 | Inositol 1,4,5-trisphosphate receptor (InsP3R) | 5 | 43 (33) | P29993 | Second messenger that mediates the release of intracellular calcium/Calcium transport |
| 186 | Cytoplasmic polyadenylation element-binding protein 3 | 5 | 35 (26) | Q6E3D5 | RNA binding *Caenorhabditis briggsae* |
| 187 | Dynein beta chain, ciliary | 22 | 57 (26) | P39057 | Cilium biogenesis/degradation *Anthocidaris crassispina* (Sea urchin) |
| 188 | Sodium channel protein 60E | 6 | 30 (26) | Q9W0Y8 | Sensory transduction |
| 189 | Nuclear hormone receptor E75 | 3 | 30 (26) | O77245 | Regulation of transcription, DNA-dependent *Metapenaeus ensis* (Greasyback shrimp) (Sand shrimp) |
| 190 | Enolase | 4 | 29 (26) | Q27655 | Glycolysis *Fasciola hepatica* (Liver fluke) |
| 191 | DNA topoisomerase 2 | 6 | 28 (26) | O16140 | DNA topological change |
| 192 | U17-ctenitoxin-Pn1a | 2 | 27 (26) | P83996 | Neurotoxin (spastic paralysis and death in mice) |
| 193 | 60S ribosomal protein L13a | 12 | 31 (26) | Q8MUR4 | Ribonucleoprotein Choristoneura parallela (Spotted fireworm moth) |
| 194 | Non-neuronal cytoplasmic intermediate filament protein | 5 | 27 (25) | P22488 | Structural molecule activity *Helix aspersa* (Brown garden snail) |
| 195 | Venom allergen 5 | 3 | 30 (26) | P81657 | Disulfide bond *Vespa mandarinia* (Hornet) |
| 196 | Nucleolar complex protein 3 homolog | 13 | 28 (26) | Q61LN7 | CBF/MAK21 family *Caenorhabditis briggsae* |
| 197 | 40S ribosomal protein S3a | 4 | 38 (26) | P49395 | Translation *Aplysia californica* (California sea hare) |
| 198 | 60S ribosomal protein L13a | 11 | 27 (26) | Q962U0 | Ribonucleoprotein *Spodoptera frugiperda* (Fall armyworm) |
| 199 | Insulin-like receptor | 4 | 28 (25) | Q93105 | Insulin receptor signaling pathway *Aedes aegypti* (Yellowfever mosquito) (*Culex aegypti*) |
| 200 | Squidulin | 3 | 27 (26) | P14533 | Calcium ion binding *Loligo pealeii* (Longfin inshore squid) (Loligo pallida) |
| 201 | Protein ultraspiracle homolog | 4 | 40 (25) | P54779 | Regulation of transcription, DNA-dependent *Manduca sexta* (Tobacco hawkmoth) (Tobacco hornworm) |
| 202 | Resact receptor | 4 | 34 (25) | P11528 | cGMP biosynthesis *Arbacia punctulata* (Punctuate sea urchin) |
| 203 | Alpha-scruin | 6 | 30 (25) | Q25390 | Actin bundling protein found in the acrosomal sperm process *Limulus polyphemus* (Atlantic horseshoe crab) |
| 204 | Histone H2B.2, embryonic | 3 | 28 (25) | P02288 | Nucleosome assembly *Psammechinus miliaris* (Sand sea urchin) |
| 205 | Puff II/9-2 protein | 3 | 26 (25) | P22312 | Glycoprotein *Sciara coprophila* (Fungus gnat) |
| 206 | Arylphorin subunit A4 | 5 | 26 (25) | P28513 | Transport *Calliphora vicina* (Blue blowfly) (Calliphora erythrocephala) |
| 207 | Contryphan-Sm | 2 | 31 (24) | P58787 | Pathogenesis *Conus stercusmuscarum* (Fly-specked cone) |
| 208 | 40S ribosomal protein S23 | 2 | 28 (26) | Q9GRJ3 | Ribonucleoprotein *Lumbricus rubellus* (Humus earthworm) |
| 209 | GTP-binding nuclear protein Ran | 8 | 41 (25) | P38542 | Protein transport *Brugia malayi* (Filarial nematode worm) |
| 210 | 77 kDa echinoderm microtubule-associated protein | 6 | 28 (25) | Q26613 | Proteolysis *Strongylocentrotus purpuratus* (Purple sea urchin) |
| 211 | Actin, muscle-type A2 | 4 | 27 (26) | P07837 | Actin family *Bombyx mori* (silk moth) |
| 213 | Nitric oxide synthase, salivary gland | 3 | 31 (25) | Q26240 | Nitric oxide biosynthetic process *Rhodnius prolixus* (Triatomid bug) |
| 214 | Vitellogenin-2 | 11 | 30 (25) | Q9BPS0 | Lipid transport *Periplaneta americana* (American cockroach) (Blatta americana) |
| 215 | S-crystallin 3 | 4 | 28 (25) | Q25626 | Structural components of squids and octopi eye lens *Octopus vulgaris* |
| 216 | Major yolk protein | 5 | 27 (25) | P19615 | Cellular iron ion homeostasis *Strongylocentrotus purpuratus* (purple sea urchin) |
| 217 | V-type proton ATPase subunit H | 3 | 26 (25) | Q9U5N0 | Hydrogen ion transport/Ion transport, *Manduca sexta* (Tobacco hawkmoth) (Tobacco hornworm) |
| 218 | Spindle-defective protein 2 | 4 | 40 (25) | Q61DP2 | Cell cycle *Caenorhabditis briggsae* |
| 219 | Calcium-dependent protein kinase C | 5 | 33 (25) | Q16974 | Protein amino acid phosphorylation *Aplysia californica* (California sea hare) |
| 220 | 40S ribosomal protein S24 | 2 | 29 (25) | Q962Q6 | Ribonucleoprotein *Spodoptera frugiperda* (Fall armyworm) |
| 221 | Glutathione S-transferase class-mu 28 kDa isozyme | 5 | 29 (25) | P30114 | Transferase *Schistosoma haematobium* (Blood fluke) |
| 222 | Metallothionein | 2 | 27 (25) | P55953 | High content of cysteine residues that bind various heavy metals *Sterechinus neumayeri* (Antarctic sea urchin) |
| 223 | Orphan steroid hormone receptor 2 | 6 | 51 (26) | Q26622 | Transcription regulation *Strongylocentrotus purpuratus* (Purple sea urchin) |
| 224 | Protein SpAN | 4 | 30 (26) | P98068 | Multicellular organismal development *Strongylocentrotus purpuratus* (Purple sea urchin) |
| 225 | Metallothionein-A | 2 | 27 (26) | Q26497 | High content of cysteine residues that bind various heavy metals *Sphaerechinus granularis* (Purple sea urchin) |
| 226 | Bifunctional arginine demethylase and lysyl-hydroxylase psr-1 | 3 | 26 (25) | Q623U2 | Transcription regulation *Caenorhabditis briggsae* |
| 227 | Myosin heavy chain, striated muscle | 12 | 30 (25) | P24733 | Motor protein *Aequipecten irradians (Bay scallop) (Argopecten irradians)* |
| 228 | Luciferin 4-monooxygenase | 3 | 36 (26) | Q01158 | Luminescence *Luciola lateralis* (Firefly) |
| 229 | Sex-determining transformer protein 2 | 7 | 31 (26) | Q17307 | Sexual differentiation *Caenorhabditis briggsae* |
| 230 | Tropomyosin | 6 | 31 (25) | Q8T380 | Central role in the calcium dependent regulation of muscle contraction *Lepisma saccharina* (silverfish) |
| 231 | Molt-inhibiting hormone-like | 2 | 27 (26) | P55322 | Neuropeptide signaling pathway *Litopenaeus vannamei* (Whiteleg shrimp) (Penaeus vannamei) |
| 232 | Dihydropyrimidinase 1 | 5 | 28 (25) | Q60Q85 | Hydrolase *Caenorhabditis briggsae* |
| 233 | Egg-lysin | 3 | 26 (25) | Q01383 | Fertilization *Haliotis sorenseni* (White abalone) |
