# Supplementary material for: Diet-Induced Over-Expression of Flightless-I Protein and Its Relation to Flightlessness in Mediterranean Fruit Fly, Ceratitis capitata
Source: PLoS One. 2013 Dec 3;8(12):e81099. doi: 10.1371/journal.pone.0081099 (PMC3849048; doi:10.1371/journal.pone.0081099)
Supplement: Table S5 — Medfly protein identifiers (269) converted to the corresponding Entrez Genes in Human ortholog. (DOC) [file pone.0081099.s005.doc]

**Supporting Information (SI)**

**Diet-induced over-expression of flightless-I protein and its relation to flightlessness in Mediterranean fruit fly, *Ceratitis capitata***

Il Kyu Cho^1^, Chiou Ling Chang^2^ and Qing X. Li^1^*

^1^ Department of Molecular Biosciences and Bioengineering, University of Hawaii, Honolulu, Hawaii, USA.

^2^ U.S. Pacific Basin Agricultural Research Center, Hilo, Hawaii, USA.

**Table S5. Medfly protein identifiers (269) converted to the corresponding Entrez Genes in Human ortholog**.

| Accession No. (UniProt Ids) | Entrez gene symbols^*^ | Under^†^ (-1) / Over (1) / Overlap (0) | Protein /Entrez gene names | Locations | Type(s) | Biological functions |
| --- | --- | --- | --- | --- | --- | --- |
| Q00449 | ABCB4 | 0 | Multidrug resistance protein homolog 49/ATP-binding cassette, sub-family B (MDR/TAP), member 4 | Plasma membrane | transporter | Transport |
| P91660 | ABCC4 | 0 | Multidrug resistance-associated protein lethal(2)03659/ATP-binding cassette, sub-family C (CFTR/MRP), member 4 | Plasma membrane | transporter | Transport |
| P02574 | ACTG2 | 1 | Actin, larval muscle/actin, gamma 2, smooth muscle, enteric | Cytoplasm | other | Ubiquitously expressed in all eukaryotic cells. |
| P32865 | ADRBK2 | -1 | G protein-coupled receptor kinase 1/adrenergic, beta, receptor kinase 2 | Cytoplasm | kinase | Specifically phosphorylates the activated forms of G protein-coupled receptors |
| Q9NGC3 | AGAP3 | -1 | Centaurin-gamma-1A/ArfGAP with GTPase domain, ankyrin repeat and PH domain 3 | Nucleus | transcription regulator | Regulation of ARF GTPase activity |
| P07764 | Aldoal1 | 1 | Fructose-bisphosphate aldolase/aldolase A-like 1 | unknown | other | Glycolysis |
| Q9VCA8 | ANKHD1-EIF4EBP3 | 0 | Ankyrin repeat and KH domain-containing protein mask | unknown | other | Antimicrobial humoral response |
| P91926 | AP2A2 | 0 | AP-2 complex subunit alpha/adaptor-related protein complex 2, alpha 2 subunit | Cytoplasm | transporter | Endocytosis |
| P14599 | APP | 0 | Beta-amyloid-like protein/amyloid beta (A4) precursor protein | Plasma membrane | other | Differentiation/Neurogenesis |
| Q94981 | ARIH1 | -1 | Protein ariadne-1/ariadne homolog, ubiquitin-conjugating enzyme E2 binding protein, 1 (*Drosophila*) | Cytoplasm | enzyme | Ubl conjugation pathway |
| Q9VXT7 | ASRGL1 | -1 | L-asparaginaseCG7860/asparaginase like 1 | Cytoplasm | enzyme | Asparagine catabolic process via L-aspartate |
| Q05825 | ATP5B | -1 | ATP synthase subunit beta | Cytoplasm | transporter | ATP synthesis coupled proton transport |
| Q9W4P5 | ATP6V0D1 | 1 | V-type proton ATPase subunit d 1/ATPase, H+ transporting, lysosomal 38kDa, V0 subunit d1 | Cytoplasm | transporter | Hydrogen ion transport |
| Q9V7D2 | ATP6V1D | 1 | V-type proton ATPase subunit D 1/ATPase, H+ transporting, lysosomal 34kDa, V1 subunit D | Cytoplasm | transporter | Hydrogen ion transport |
| Q9GQN5 | ATRX | 0 | Alpha thalassemia/mental retardation syndrome X-linked | Nucleus | transcription regulator | DNA damage |
| Q9V407 | AXIN2 | -1 | Axin/axin 2 | Cytoplasm | other | Wnt signaling pathway |
| P16568 | BICD1 | 0 | Protein bicaudal D/bicaudal D homolog 1 (*Drosophila*) | Cytoplasm | other | Germarium-derived oocyte fate determination |
| P07713 | BMP2 | 1 | Protein decapentaplegic/bone morphogenetic protein 2 | Extracellular space | growth factor | BMP (bone morphogenetic protein) signaling pathway |
| Q9W0T1 | Bptf | 0 | Nucleosome-remodeling factor subunit NURF301/bromodomain PHD finger transcription factor | Nucleus | transcription regulator | Dendrite morphogenesis |
| P11346 | BRAF | 0 | Raf homolog serine/threonine-protein kinase phl/v-raf murine sarcoma viral oncogene homolog B1 | Cytoplasm | enzyme | Border follicle cell migration |
| P13709 | BRD3 | -1 | Homeotic protein female sterile/bromodomain containing 3 | Nucleus | kinase | Multicellular organismal development |
| Q24270 | CACNA1D | 0 | Voltage-dependent calcium channel type D subunit alpha-1/calcium channel, voltage-dependent, L type, alpha 1D | Plasma membrane | ion channel | Calcium transport |
| P05990 | CAD | 1 | CAD protein/carbamoyl-phosphate synthetase 2, aspartate transcarbamylase, and dihydroorotase | Cytoplasm | enzyme | Pyrimidine biosynthesis |
| Q9NHE5 | CADPS | 1 | Ca++-dependent secretion activator | Plasma membrane | other | Exocytosis |
| Q9I7D3 | CAPRIN1 | -1 | Caprin/cell cycle associated protein 1 | Plasma membrane | other | Monolayer-surrounded lipid storage body |
| Q8SWR2 | CCDC64 | -1 | Bicaudal D-related protein/coiled-coil domain containing 64 | Cytoplasm | other | Belongs to the BICDR family |
| P54733 | CCNE1 | -1 | G1/S-specific cyclin-E/cyclin E1 | Nucleus | transcription regulator | Essential for the control of the cell cycle at the G1/S (start) transition |
| O96433 | CCNT1 | -1 | Cyclin-T 1 | Nucleus | transcription regulator | Actin filament organization |
| P48605 | CCT3 | 1 | T-complex protein 1/chaperonin containing TCP1 | Cytoplasm | other | Mitotic spindle organization |
| Q8T626 | CDAN1 | -1 | Protein disks lost/congenital dyserythropoietic anemia, type I | Nucleus | other | Cellular process |
| Q24276 | CDC37 | -1 | Hsp90 co-chaperone Cdc37/cell division cycle 37 homolog | Cytoplasm | other | Cell cycle |
| Q9W1B0 | CDC42BPA | 0 | Serine/threonine-protein kinase Genghis Khan/CDC42 binding protein kinase alpha (DMPK-like) | Cytoplasm | kinase | Actin polymerization or depolymerization |
| Q9VJB6 | CDH8 | -1 | Putative neural-cadherin 2/cadherin 8, type 2 | Plasma membrane | other | Calcium dependent cell adhesion proteins |
| Q9VP22 | CDK12 | -1 | Cell division cycle 2-like protein kinase CG7597/cyclin-dependent kinase 12 | Nucleus | kinase | Protein amino acid phosphorylation |
| Q9V5N8 | CELSR1 | -1 | Protocadherin-like wing polarity protein stan/cadherin, EGF LAG seven-pass G-type receptor 1 (flamingo homolog, *Drosophila*) | Plasma membrane | G-protein coupled receptor | Cell adhesion/Cell membrane |
| Q7KU24 | CHD2 | 0 | Chromodomain-helicase-DNA-binding protein 2 | Nucleus | enzyme | Chromatin assembly or disassembly |
| O61661 | CHEK1 | -1 | Serine/threonine-protein kinase grp/CHK1 checkpoint homolog (S. pombe) | Nucleus | kinase | Cell cycle |
| O61735 | CLOCK | -1 | Circadian locomoter output cycles protein kaput/clock homolog (mouse) | Nucleus | transcription regulator | Behavioral response to cocaine |
| Q8IRB5 | CLSPN | -1 | Claspin | Nucleus | other | Phosphoprotein |
| Q9V3G6 | CNOT10 | 0 | CCR4-NOT transcription complex | Cytoplasm | other | Phosphoprotein |
| Q95TN4 | COG4 | 1 | Conserved oligomeric Golgi complex subunit 4/component of oligomeric golgi complex 4 | Cytoplasm | transporter | Protein transport |
| P08120 | COL4A5 | 1 | Collagen, type IV, alpha 5 | Extracellular space | other | Dorsal closure |
| Q9VSR3 | CPEB2 | -1 | RNA-binding protein orb2/cytoplasmic polyadenylation element binding protein 2 | Cytoplasm | other | Long-term memory |
| Q9V726 | CPSF1 | 1 | Cleavage and polyadenylation specific factor 1, 160kDa | Nucleus | other | mRNA processing |
| Q24400 | CSRP1 | 1 | Muscle LIM protein Mlp84B/cysteine and glycine-rich protein 1 | Nucleus | other | Muscle organ development |
| Q9W147 | CTDP1 | 1 | Fcp1/CTD (carboxy-terminal domain, RNA polymerase II, polypeptide A) phosphatase, subunit 1 | Nucleus | phosphatase | Hydrolase |
| Q9VXT5 | CWF19L2 | -1 | CWF19-like 2, cell cycle control (S. pombe) | unknown | other | Phosphoprotein |
| Q24292 | DCHS1 | 1 | Dachsous 1 (*Drosophila*) | Plasma membrane | other | Cell adhesion |
| P19109 | DDX17 | 1 | ATP-dependent RNA helicase p62/DEAD (Asp-Glu-Ala-Asp) box polypeptide 17 | Nucleus | enzyme | RNA-mediated gene silencing |
| Q9VD51 | DDX18 | 0 | ATP-dependent RNA helicase pitchoune/DEAD (Asp-Glu-Ala-Asp) box polypeptide 18 | Nucleus | enzyme | Helicase/Hydrolase |
| Q86B47 | DDX31 | 1 | ATP-dependent RNA helicase CG8611/DEAD (Asp-Glu-Ala-Asp) box polypeptide 31 | Nucleus | enzyme | ATP-dependent RNA helicase/hydrolase |
| P09052 | DDX4 | 1 | ATP-dependent RNA helicase vasa/DEAD (Asp-Glu-Ala-Asp) box polypeptide 4 | Nucleus | enzyme | Differentiation/ Oogenesis |
| Q01583 | DGKB | -1 | Diacylglycerol kinase, beta 90kDa | Cytoplasm | kinase | Activation of protein kinase C activity by G-protein coupled receptor protein signaling pathway |
| Q09103 | DGKZ | 1 | Eye-specific diacylglycerol kinase/diacylglycerol kinase, zeta | Cytoplasm | kinase | Phospholipid turnover within the photoreceptor |
| P24785 | DHX9 | -1 | Dosage compensation regulator/DEAH (Asp-Glu-Ala-His) box polypeptide 9 | Nucleus | enzyme | Axon extension |
| P31007 | DLG1 | 0 | Disks large 1 tumor suppressor protein/discs, large homolog 1 (*Drosophila*) | Plasma membrane | kinase | Cell adhesion/Cell junction |
| Q9VDW6 | DMD | 0 | Dystrophin | Plasma membrane | other | Establishment of cell polarity |
| Q7KVQ2 | DNAI1 | 0 | Dynein, axonemal, intermediate chain 1 | Extracellular Space | other | ATPase activity, uncoupled |
| A1ZBE8 | DOPEY1 | 1 | Dopey family member 1 | unknown | other | Protein traffic between late Golgi and early endosomes |
| P37276 | DYNC1H1 | 0 | Dynein, cytoplasmic 1, heavy chain 1 | Cytoplasm | peptidase | RNA transport |
| P83102 | DYRK2 | -1 | Dual-specificity tyrosine-(Y)-phosphorylation regulated kinase 2 | Cytoplasm | kinase | Protein amino acid phosphorylation |
| Q9V3D5 | DYRK4 | -1 | Dual-specificity tyrosine-(Y)-phosphorylation regulated kinase 4 | Nucleus | kinase | Olfactory behavior |
| Q9V9Z9 | EBNA1BP2 | 0 | rRNA-processing protein EBP2/EBNA1 binding protein 2 | Nucleus | other | Ribosome biogenesis |
| Q9NIV1 | EIF2AK3 | -1 | Eukaryotic translation initiation factor 2-alpha kinase 3 | Cytoplasm | kinase | Stress response |
| Q9VE34 | EPG5 | 1 | UPF0493 protein CG14299/ectopic P-granules autophagy protein 5 homolog | unknown | other | Phosphoprotein |
| Q9VDE6 | EXOC6B | -1 | Exocyst complex component 6B | unknown | other | Exocytosis/cytoplasmic vesicle |
| Q9V730 | EXT1 | -1 | Exostosin-1 (Protein tout-velu) | Cytoplasm | enzyme | Wnt signaling pathway |
| A1ZBW7 | FAM21A | 0 | Protein FAM21/family with sequence similarity 21, member A | Cytoplasm | other | Phosphoprotein |
| O16129 | FARS2 | 1 | Phenylalanyl-tRNA synthetase 2 | Cytoplasm | enzyme | Protein biosynthesis |
| Q9VW71 | FAT1 | -1 | Fat-like cadherin-related tumor suppressor /FAT tumor suppressor homolog 1 (*Drosophila*) | Plasma membrane | other | Cell adhesion |
| P33450 | FAT4 | 0 | Cadherin-related tumor suppressor/FAT tumor suppressor homolog 4 (*Drosophila*) | unknown | other | Cell adhesion |
| Q9W1V3 | FBL | 1 | rRNA 2'-O-methyltransferase fibrillarin/fibrillarin | Nucleus | other | rRNA processing |
| Q9VZF4 | FBXW7 | -1 | F-box and WD repeat domain containing 7 | Nucleus | transcription regulator | Cell cycle/Ubl conjugation pathway |
| P18106 | FER | 1 | Tyrosine-protein kinase Fps85D/fer (fps/fes related) tyrosine kinase | Cytoplasm | kinase | Actin filament bundle assembly |
| Q9VL78 | FKBP4 | 0 | FK506 binding protein 4, 59kDa | Nucleus | enzyme | Phototransduction (inhibition or prevention of Ca^2+^ induced stimulation of the trpl ion channel) |
| Q24020 | FLII | 1 | Protein flightless-1/flightless I homolog (*Drosophila*) | Nucleus | other | Flight behavior/structural role in indirect flight muscle |
| O61492 | FLOT2 | 0 | Flotillin 2 | Plasma membrane | other | Cell adhesion |
| Q9VUC6 | FMNL2 | -1 | Formin-like protein CG32138/formin-like 2 | Cytoplasm | other | Actin cytoskeleton organization |
| Q9VT28 | FRYL | -1 | Protein furry/FRY-like | unknown | other | Transcription regulation/Phosphoprotein |
| Q9VEP1 | FTSJ1 | 1 | Putative ribosomal RNA methyltransferase CG5220/FtsJ homolog 1 (E. coli) | unknown | enzyme | rRNA processing |
| Q27294 | FUS | 1 | RNA-binding protein cabeza/fused in sarcoma | Nucleus | transcription regulator | Nuclear mRNA splicing, via spliceosome |
| Q9NFU0 | FXR1 | -1 | Fragile X mental retardation, autosomal homolog 1 | Cytoplasm | other | RNA-mediated gene silencing |
| Q9VVX3 | FZD8 | 0 | Frizzled-2/frizzled family receptor 8 | Plasma membrane | G-protein coupled receptor | Wnt signaling pathway |
| Q24352 | GABRA3 | -1 | Gamma-aminobutyric acid (GABA) A receptor, alpha 3 | Plasma membrane | ion channel | Ion transport |
| Q8MVS5 | GALNT11 | 0 | Polypeptide N-acetyl galactosaminyl transferase 35A/UDP-N-acetyl-alpha-D-galactosamine:polypeptide N-acetylgalactosaminyltransferase 11 | Cytoplasm | enzyme | Oligosaccharide biosynthetic process/Essential glycotransferase |
| P07487 | GAPDH | 1 | Glyceraldehyde-3-phosphate dehydrogenase | Cytoplasm | enzyme | Glycolysis |
| Q9VZ08 | GAPVD1 | -1 | Receptor-mediated endocytosis protein 6/GTPase activating protein and VPS9 domains 1 | Cytoplasm | other | Endocytosis |
| Q7KVQ0 | GAR1 | 1 | H/ACA ribonucleoprotein complex  / GAR1 ribonucleoprotein | Nucleus | ion channel | Ribosome biogenesis/rRNA processing |
| P00967 | GART | 1 | Trifunctional purine biosynthetic protein adenosine-3 | Cytoplasm | enzyme | Purine biosynthesis |
| Q86BY9 | GEMIN5 | -1 | Protein rigor mortis/gem (nuclear organelle) associated protein 5 | Nucleus | other | Transcription regulation |
| Q9VRJ0 | GEN1 | 1 | Flap endonuclease GEN/Gen homolog 1, endonuclease (*Drosophila*) | unknown | enzyme | DNA catabolic process, endonucleolytic |
| Q9VNG1 | GGNBP2 | 1 | Gametogenetin binding protein 2 | unknown | other | Not known |
| Q7KQM6 | GIGYF2 | 0 | PERQ amino acid-rich with GYF domain-containing protein CG11148 /GRB10 interacting GYF protein 2 | unknown | other | Belongs to the PERQ family |
| P54385 | GLUD1 | -1 | Glutamate dehydrogenase 1 | Cytoplasm | enzyme | NADH oxidation |
| Q9VMW9 | GMDS | -1 | GDP-mannose 4,6-dehydratase | Cytoplasm | enzyme | GDP-L-fucose biosynthetic process/GDP-mannose metabolic process/intracellular |
| Q05913 | GTF2F1 | 1 | General transcription factor IIF, polypeptide 1, 74kDa | Nucleus | transcription regulator | Positive regulation of transcription |
| Q9VM75 | HEATR1 | 1 | HEAT repeat containing 1 | Nucleus | other | Ribosome biogenesis/rRNA processing |
| Q9VR91 | HERC2 | 0 | E3 ubiquitin-protein ligase HERC2 /hect domain and RLD 2 | Cytoplasm | enzyme | Ubl conjugation pathway |
| Q960X8 | HGS | -1 | Hepatocyte growth factor-regulated tyrosine kinase substrate | Cytoplasm | other | Border follicle cell migration |
| Q9W040 | IFT172 | 0 | Intraflagellar transport 172 homolog | Nucleus | other | Maintenance and formation of cilia |
| P09208 | IGF1R | 1 | Insulin-like growth factor 1 receptor | Plasma membrane | transmembrane receptor | Differentiation/Growth regulation/Neurogenesis |
| P91928 | IMMT | 0 | Putative mitochondrial inner membrane protein/inner membrane protein | Cytoplasm | other | Integral to mitochondrial inner membrane |
| Q9W485 | INTS6 | 0 | Lethal (1) G0060, isoform A/integrator complex subunit 6 | Nucleus | enzyme | Not known |
| Q24247 | ITGA7 | 0 | Integrin, alpha 7 | Plasma membrane | other | Cell adhesion |
| P12080 | ITGAV | -1 | Integrin, alpha V (vitronectin receptor, alpha polypeptide, antigen CD51) | Plasma membrane | other | Cell adhesion |
| P29993 | ITPR1 | -1 | Inositol 1,4,5-trisphosphate receptor (InsP3R), type 1 | Cytoplasm | ion channel | Second messenger that mediates the release of intracellular calcium/Calcium transport |
| P18168 | JAG2 | 0 | Serrate protein/Jagged 2 | Extracellular Space | growth factor | Differentiation/Notch signaling pathway |
| Q9VD28 | JMJD6 | -1 | Bifunctional arginine demethylase and lysyl-hydroxylase PSR/jumonji domain containing 6 | Plasma membrane | other | Dioxygenase |
| Q02280 | KCNH5 | 0 | Potassium voltage-gated channel, subfamily H (eag-related), member 5 | Plasma Membrane | ion channel | Differentiation/Ion transport/Neurogenesis |
| Q94526 | KCNK17 | 0 | Open rectifier potassium channel protein 1/potassium channel, subfamily K, member 17 | Plasma membrane | ion channel | Ion transport |
| Q9V6L0 | KDM4B | 1 | Lysine (K)-specific demethylase 4B | unknown | other | Histone H3-K36 demethylation |
| Q9VMJ7 | KDM5A | -1 | lysine (K)-specific demethylase 5A | Nucleus | transcription regulator | Histone H3-K4 demethylation |
| Q9V677 | KIAA0368 | -1 | Proteasome-associated protein ECM29 | Cytoplasm | other | Protein catabolic process |
| Q9VQ60 | KIAA2013 | 1 | Drosophila melanogaster, CG7289 | unknown | other | Not known |
| P46863 | KIF11 | -1 | Bipolar kinesin KRP-130/kinesin family member 11 | Nucleus | other | Cell cycle/Microtubule |
| Q8SXT9 | KIF23 | 0 | Kinesin family member 23 | Cytoplasm | other | Contractile ring contraction involved in cell cycle cytokinesis |
| Q960Z0 | KIF2A | 1 | Kinesin-like protein Klp10A/kinesin heavy chain member 2A | Cytoplasm | other | Cell cycle |
| P17210 | KIF5B | -1 | Kinesin heavy chain/kinesin family member 5B | Cytoplasm | other | Axon cargo transport/microtubule |
| P46824 | KLC1 | 1 | Kinesin light chain 1 | Cytoplasm | other | Microtubule-associated force-producing  protein that may play a role in organelle  transport |
| Q04652 | KLHL3 | 0 | Ring canal kelch protein/kelch-like 3 | unknown | other | Differentiation/Oogenesis |
| Q00174 | LAMA5 | 0 | Laminin, alpha 5 | Extracellular space | other | Cell adhesion (mediation of the attachment) |
| P11046 | LAMB2 | 1 | Laminin, beta 2 | Extracellular space | enzyme | Cell adhesion |
| P15215 | LAMC1 | -1 | Laminin, gamma 1 (formerly LAMB2) | Extracellular space | other | Cell adhesion |
| Q9VAW5 | LARP1 | 1 | La-related protein/La ribonucleoprotein | Cytoplasm | other | Mitochondrion inheritance |
| Q94546 | LEO1 | 0 | Another transcription unit protein/Leo1, Paf1/RNA polymerase II complex component, homolog | Nucleus | other | Phosphoprotein |
| Q9BN18 | LGR5 | 1 | Leucine-rich repeat containing G protein-coupled receptor 5 | Plasma membrane | G-protein coupled receptor | G-protein coupled receptor signaling |
| Q8IR79 | LIMK1 | 0 | LIM domain kinase 1 | Cytoplasm | kinase | Actin cytoskeleton organization |
| A1Z9E2 | LIN54 | -1 | Lin-54 homolog | unknown | other | Adult lifespan |
| P08111 | LLGL1 | 0 | Lethal giant larvae homolog 1 | Cytoplasm | other | Cell cycle |
| Q9VXY2 | MADD | 0 | MAP-kinase activating death domain | Cytoplasm | other | Activation of MAPK activity |
| Q9W596 | MAP1B | 0 | Microtubule-associated protein 1B | Cytoplasm | other | Axon cargo transport |
| Q23977 | MAP2K7 | -1 | Dual specificity mitogen-activated protein kinase kinase hemipterous/mitogen-activated protein kinase kinase 7 | Cytoplasm | kinase | JNK (c-Jun N-terminal kinase) cascade |
| Q8MSQ4 | MAP3K4 | 0 | Mekk1, isoform B/mitogen-activated protein kinase kinase kinase 4 | Cytoplasm | kinase | MAPKKK cascade |
| Q9V719 | MAPKAP1 | -1 | Stress-activated map kinase-interacting protein 1/mitogen-activated protein kinase associated protein 1 | Cytoplasm | other | Apoptosis |
| Q9VFL5 | MARS2 | -1 | Methionyl-tRNA synthetase 2, mitochondrial | Cytoplasm | enzyme | Protein biosynthesis |
| Q9VIE6 | MCM10 | -1 | Protein MCM10/minichromosome maintenance complex component 10 | Nucleus | other | DNA replication |
| Q26454 | MCM4 | -1 | DNA replication licensing factor /minichromosome maintenance complex component 4 | Nucleus | enzyme | Mitotic DNA replication |
| Q9VP05 | MED1 | 1 | Mediator of RNA polymerase II transcription subunit 1/mediator complex subunit 1 | Nucleus | transcription regulator | Regulation of transcription from RNA polymerase II promoter |
| Q7KTX8 | MED13L | 0 | Mediator of RNA polymerase II transcription subunit 13 | Nucleus | other | Transcription regulation |
| Q9VSF2 | MED24 | 1 | Mediator of RNA polymerase II transcription subunit 24 | Nucleus | transcription regulator | Transcription regulation |
| Q9VCE6 | METTL3 | 1 | N6-adenosine-methyltransferase MT-A70-like protein/methyltransferase like 3 | Nucleus | enzyme | RNA methylation |
| Q7YU24 | MFN2 | -1 | Transmembrane GTPase Marf/mitofusin 2 | Cytoplasm | enzyme | Mitochondrial fusion |
| Q9VUX2 | MIB1 | -1 | E3 ubiquitin-protein ligase mind-bomb/mindbomb homolog 1 | Cytoplasm | other | Notch signaling pathway/Ubl conjugation pathway |
| Q9VKJ1 | MPND | 0 | MPN domain containing | unknown | other | Probable protease |
| Q9VFB2 | MRPS10 | 1 | Mitochondrial ribosomal protein S10 | Cytoplasm | other | Translation |
| P43248 | MSH2 | 1 | DNA mismatch repair protein spellchecker 1/mutS homolog 2, colon cancer, nonpolyposis type 1 | Nucleus | enzyme | DNA damage/DNA repair |
| Q9VUM0 | MSH6 | 0 | DNA mismatch repair protein Msh6/ mutS homolog 6 | Nucleus | enzyme | Post-replicative DNA-mismatch repair |
| P50536 | MSL3 | 1 | Male-specific lethal 3 | Nucleus | transcription regulator | Chromatin assembly or disassembly |
| Q9NB71 | MYCBP2 | 1 | E3 ubiquitin-protein ligase highwire / MYC binding protein 2 | Nucleus | enzyme | Ubl conjugation pathway |
| Q99323 | MYH10 | -1 | Myosin, heavy chain 10, non-muscle | Cytoplasm | other | Malpighian tubule morphogenesis |
| P05661 | MYH7 | 0 | Myosin, heavy chain 7, cardiac muscle, beta | Cytoplasm | enzyme | Epithelial cell migration |
| P10676 | MYO3A | 0 | Neither inactivation nor after potential protein C/myosin IIIA | Cytoplasm | kinase | Sensory transduction/Vision |
| Q01989 | MYO6 | -1 | Myosin VI | Cytoplasm | other | Actin cytoskeleton organization |
| Q9VJ62 | NAF1 | -1 | H/ACA ribonucleoprotein complex non-core subunit NAF1/nuclear assembly factor 1 homolog | unknown | other | Ribosome biogenesis/rRNA processing |
| Q9VIQ9 | NAV2 | -1 | Protein sickie/neuron navigator 2 | Nucleus | other | Immune response/Innate immunity |
| Q9W4E2 | NBEA | 0 | Neurobeachin | Cytoplasm | other | Compound eye cone cell differentiation/eye photoreceptor cell development |
| P55162 | NCKAP1 | 1 | Membrane-associated protein Hem/NCK-associated protein 1 | Plasma Membrane | other | Axonogenesis |
| P13469 | Ncl | -1 | DNA-binding protein modulo / nucleolin | Nucleus | other | Cell proliferation |
| Q9V3L1 | NDST2 | -1 | N-deacetylase/N-sulfotransferase (heparan glucosaminyl) 2 | Cytoplasm | enzyme | Wnt signaling pathway |
| Q9VVI3 | NEDD4 | 1 | E3 ubiquitin-protein ligase Nedd-4/neural precursor cell expressed, developmentally down-regulated 4 | Cytoplasm | enzyme | Notch signaling pathway/Ubl conjugation pathway |
| Q94527 | NFKB1 | 0 | Nuclear factor of kappa light polypeptide gene enhancer in B-cells 1 | Nucleus | transcription regulator | Immune response |
| Q9VI82 | NOC3L | 1 | Nucleolar complex associated 3 | Nucleus | other | Binding |
| Q9V3P2 | NOL10 | 0 | Nucleolar protein 10 | Nucleus | other | Protein binding |
| Q9VWD4 | NOL8 | 1 | RNA-binding protein CG14230/nucleolar protein 8 | Nucleus | other | RNA binding |
| Q9VEJ2 | NOP14 | -1 | NOP14 nucleolar protein homolog (yeast) | Nucleus | other | Ribosome biogenesis/rRNA processing |
| P07207 | NOTCH1 | 0 | Neurogenic locus notch protein/ notch 1 | Plasma membrane | transcription regulator | Notch signaling pathway |
| Q9V3D8 | NPEPL1 | -1 | Aminopeptidase-like 1 | unknown | peptidase | Proteolysis |
| Q9VUB4 | NPRL3 | 0 | UPF0171 protein CG8783/nitrogen permease regulator-like 3 | unknown | other | Protein binding |
| P46461 | NSF | 1 | Vesicular-fusion ATPase 1/N-ethylmaleimide-sensitive factor | Cytoplasm | transporter | ER-Golgi transport |
| Q9GYU8 | NUP88 | 1 | Nucleoporin 88kDa | Nucleus | transporter | Immunity/Antimicrobial humoral response |
| O01668 | OPN4 | 1 | opsin 4 | Plasma membrane | G-protein coupled receptor | Sensory transduction |
| O16810 | ORC1 | 0 | Origin recognition complex, subunit 1 | Nucleus | other | DNA replication |
| Q24168 | ORC2 | -1 | origin recognition complex, subunit 2 | Nucleus | other | DNA replication/nucleus |
| P54399 | P4HB | 1 | Protein disulfide-isomeraseprolyl/4-hydroxylase, beta polypeptide | Cytoplasm | enzyme | Cell redox homeostasis |
| Q9VN55 | PAF1 | 1 | Antimeros/Paf1, RNA polymerase II associated factor, homolog | Nucleus | other | Protein binding |
| Q868Z9 | PAPLN | 0 | Papilin, proteoglycan-like sulfated glycoprotein | Extracellular space | other | Extracellular matrix organization |
| P35875 | PARP1 | -1 | Poly (ADP-ribose) polymerase 1 | Nucleus | enzyme | Chromatin modification |
| P40427 | PBX1 | -1 | Homeobox protein extradenticle/pre-B-cell leukemia homeobox 1 | Nucleus | transcription regulator | Transcription |
| P18490 | PCNXL3 | 0 | Protein pecanex/pecanex-like 3 (*Drosophila*) | unknown | other | Differentiation/Neurogenesis |
| P12252 | PDE4D | 0 | Phosphodiesterase 4D, cAMP-specific | Cytoplasm | enzyme | Axon extension |
| Q9VLS1 | PHKB | -1 | Phosphorylase kinase, beta | Cytoplasm | kinase | Carbohydrate/Glycogen metabolism |
| O96838 | PIKFYVE | 0 | Phosphoinositide kinase, FYVE finger containing | Cytoplasm | kinase | Cellular protein metabolic process |
| O18400 | PITX2 | -1 | Paired-like homeodomain 2 | Nucleus | transcription regulator | Transcription |
| Q9W5D0 | PLEKHH2 | 1 | Pleckstrin homology domain containing, family H (with MyTH4 domain) member 2 | Cytoplasm | other | Phosphoprotein |
| P52304 | PLK1 | 0 | Polo-like kinase 1 | Nucleus | kinase | Cytokinesis |
| O97143 | PLK4 | -1 | Polo-like kinase 4 | Cytoplasm | kinase | Centriole replication |
| P26019 | POLA1 | 1 | Polymerase (DNA directed), alpha 1, catalytic subunit | Nucleus | enzyme | DNA damage/DNA repair/DNA replication |
| Q27607 | POLG | -1 | Polymerase (DNA directed), gamma | Cytoplasm | enzyme | DNA replication |
| P91875 | POLR1A | 1 | Polymerase (RNA) I polypeptide A, 194kDa | Nucleus | enzyme | Transcription |
| Q9W5D4 | POMT2 | -1 | Protein-O-mannosyltransferase 2 | Cytoplasm | enzyme | Lipid glycosylation |
| Q27597 | POR | 0 | P450 (cytochrome) oxidoreductase | Cytoplasm | enzyme | Oxidation reduction |
| P48456 | PPP3CB | 0 | Protein phosphatase 3, catalytic subunit, beta isozyme | unknown | phosphatase | Neurotransmitter secretion |
| P12370 | PRKACB | -1 | Protein kinase, cAMP-dependent, catalytic, beta | Cytoplasm | kinase | Anterior/posterior pattern formation, imaginal disc/plasma membrane |
| P82295 | PROM1 | 1 | Prominin 1 | Plasma membrane | other | Not known |
| Q05319 | PRSS8 | 1 | Protease, serine, 8 | Extracellular space | peptidase | Actin filament bundle assembly |
| P48601 | PSMC1 | -1 | 26S proteasome | Nucleus | peptidase | Cell proliferation (ATP-dependent degradation of ubiquitinated proteins) |
| P29349 | PTPN11 | -1 | Protein tyrosine phosphatase, non-receptor type 11 | Cytoplasm | phosphatase | Epidermal growth factor receptor signaling pathway |
| P16621 | PTPRD | 0 | Protein tyrosine phosphatase, receptor type, D | Plasma membrane | phosphatase | Cell adhesion |
| Q9Y105 | QARS | 0 | Glutaminyl-tRNA synthetase | Cytoplasm | enzyme | Protein biosynthesis |
| Q9W252 | RAD50 | -1 | RAD50 homolog (S. cerevisiae) | Nucleus | enzyme | Cell cycle/Chromosomal protein |
| P48555 | RALA | 1 | v-ral simian leukemia viral oncogene homolog A (ras related) | Cytoplasm | enzyme | Negative regulation of JNK cascade |
| Q9VB98 | RALGAPA1 | -1 | Ral GTPase activating protein, alpha subunit 1 (catalytic) | Cytoplasm | other | Regulation of small GTPase mediated signal transduction |
| O77086 | RAPGEF1 | -1 | Rap guanine nucleotide exchange factor | Cytoplasm | other | Ras protein signal transduction |
| Q9VRP9 | RNF40 | -1 | Ring finger protein 40 | Cytoplasm | enzyme | Notch signaling pathway/Ubl conjugation pathway |
| Q24488 | ROR1 | 1 | Receptor tyrosine kinase-like orphan receptor 1 | Plasma membrane | kinase | Central nervous system development |
| P13368 | ROS1 | 1 | C-ros oncogene 1, receptor tyrosine kinase | Plasma membrane | kinase | Sensory transduction |
| Q8T3U2 | RPS23 | 1 | Ribosomal protein S23 | Cytoplasm | translation regulator | Translation |
| Q9V3I5 | RPS6KA5 | 0 | Ribosomal protein S6 kinase, 90kDa, polypeptide 5 | Nucleus | kinase | Transcription regulation |
| Q9VJZ7 | RRP1 | 0 | Ribosomal RNA processing 1 | Nucleus | other | rRNA processing |
| Q9VYA7 | RRP12 | -1 | Ribosomal RNA processing 12 | Nucleus | other | Phosphoprotein |
| Q9V3K3 | RUVBL2 | -1 | RuvB-like 2 (E. coli) | Nucleus | transcription regulator | Wnt receptor signaling pathway |
| Q24498 | RYR2 | 0 | Ryanodine receptor 2 (cardiac) | Plasma membrane | ion channel | Calcium transport |
| Q6IDD9 | SARM1 | 1 | Sterile alpha and TIR motif containing 1 | Plasma membrane | transmembrane receptor | Innate immunity |
| Q6NNA4 | SART1 | 0 | Squamous cell carcinoma antigen recognized by T cells | Nucleus | other | Not known |
| Q9VAC8 | SASS6 | 0 | Spindle assembly 6 homolog | Cytoplasm | other | Cell cycle |
| A8JUV0 | SBNO1 | -1 | Strawberry notch homolog 1 (*Drosophila*) | unknown | enzyme | Notch signaling pathway |
| P41044 | SCGN | 1 | Secretagogin, EF-hand calcium binding protein | Cytoplasm | other | Calcium ion binding |
| Q94523 | SDHA | -1 | Succinate dehydrogenase complex, subunit A, flavoprotein (Fp) | Cytoplasm | enzyme | Electron transport |
| O97394 | SDK2 | 1 | sidekick homolog 2 (chicken) | unknown | other | Cell adhesion |
| O18391 | SERHL2 | 1 | Serine hydrolase-like 2 | Cytoplasm | enzyme | Detoxification/Digestion |
| P12297 | SFSWAP | 1 | Splicing factor, suppressor of white-apricot homolog (*Drosophila*) | Nucleus | other | Transcription regulation |
| Q9VA73 | SLC25A12 | 1 | Solute carrier family 25 (mitochondrial carrier, Aralar), member 12 | Cytoplasm | transporter | Transport |
| Q9VAY3 | SLC25A37 | 0 | Solute carrier family 25, member 37 | Cytoplasm | transporter | Mitochondrial iron ion transport |
| Q26365 | SLC25A4 | -1 | Solute carrier family 25 (mitochondrial carrier; adenine nucleotide translocator) | Cytoplasm | transporter | Transport |
| Q9VHT4 | SLC35C1 | -1 | Solute carrier family 35, member C1 | Cytoplasm | transporter | Sugar transport/Golgi apparatus |
| P24014 | SLIT2 | 1 | Slit homolog 2 (*Drosophila*) | Extracellular space | other | Differentiation/Neurogenesis |
| Q9V853 | SMURF2 | 0 | SMAD specific E3 ubiquitin protein ligase 2 | Cytoplasm | enzyme | Ubl conjugation pathway |
| Q9VUV9 | SNRNP200 | 1 | Small nuclear ribonucleoprotein 200kDa (U5) | Nucleus | enzyme | mRNA processing |
| P39736 | SNW1 | -1 | SNW domain containing 1 | Nucleus | transcription regulator | Embryonic development via the syncytial blastoderm/eye-antennal disc development |
| Q960H1 | SORD | 0 | Sorbitol dehydrogenase | Cytoplasm | enzyme | Oxidation reduction |
| Q9GQF1 | SPAG9 | 0 | Sperm associated antigen 9 | Plasma membrane | other | Axon cargo transport/regulation of JNK cascade |
| Q9U1K1 | SPIRE1 | 1 | Spire homolog 1 (*Drosophila*) | Cytoplas | other | Transport |
| P13395 | SPTAN1 | 1 | Spectrin, alpha, non-erythrocytic 1 (alpha-fodrin) | Plasma membrane | other | Cell shape |
| Q00963 | SPTBN1 | 1 | Spectrin, beta, non-erythrocytic 1 | Plasma membrane | other | Actin filament capping |
| Q9VDK7 | SRP72 | 0 | signal recognition particle 72kDa | Nucleus | kinase | SRP-dependent cotranslational protein targeting to membrane |
| Q9V9K7 | SRRT | -1 | Serrate RNA effector molecule | Nucleus | other | RNA-mediated gene silencing |
| Q05344 | SSRP1 | -1 | Structure specific recognition protein 1 | Nucleus | other | DNA damage/DNA repair |
| Q8IRG6 | SUPT16H | -1 | Suppressor of Ty 16 homolog | Nucleus | transcription regulator | Transcription regulation |
| Q9NJG9 | SUZ12 | -1 | Suppressor of zeste 12 (*Drosophila*) | Nucleus | enzyme | Dendrite morphogenesis |
| Q24546 | SYN2 | 1 | Synapsin II | Plasma membrane | other | Behavior/cell junction |
| Q71JA7 | SYNE1 | 1 | Spectrin repeat containing, nuclear envelope 1 | Nucleus | other | Actin filament organization |
| P51123 | TAF1 | -1 | TAF1 RNA polymerase II, TATA box binding protein (TBP)-associated factor | Nucleus | transcription regulator | Cell cycle |
| Q9VWY6 | TAF8 | -1 | TAF8 RNA polymerase II, TATA box binding protein (TBP)-associated factor | Nucleus | transcription regulator | Regulation of transcription |
| P20232 | TCEA3 | 0 | Transcription elongation factor A (siI), 3 | Nucleus | transcription regulator | RNA elongation |
| Q9VCP0 | TCEB3 | 1 | Transcription elongation factor B (siII), polypeptide 3 (110kDa, elongin A) | Nucleus | transcription regulator | Transcription regulation |
| P12613 | TCP1 | 0 | T-complex 1 | Cytoplasm | other | Mitotic spindle organization |
| Q9W4V8 | TIMM50 | 0 | Translocase of inner mitochondrial membrane 50 homolog | Cytoplasm | phosphatase | Protein transport |
| Q9VX39 | TMEM41B | 0 | Transmembrane protein 41B | unknown | other | Integral to membrane |
| P30189 | TOP1 | 1 | Topoisomerase (DNA) I | Nucleus | enzyme | DNA topological change |
| P15348 | TOP2B | 1 | Topoisomerase (DNA) II beta 180kDa | Nucleus | enzyme | DNA topological change |
| Q9NG98 | TOP3A | 0 | Topoisomerase (DNA) III alpha | Nucleus | enzyme | DNA topological change |
| Q9V6K1 | TPP2 | -1 | Tripeptidyl peptidase II | Cytoplasm | peptidase | Protein homooligomerization |
| Q9W2M2 | TREH | 1 | Trehalase (brush-border membrane glycoprotein) | Plasma membrane | enzyme | Trehalose metabolic process |
| Q8I8U7 | TRRAP | -1 | Transformation/transcription domain-associated protein | Nucleus | transcription regulator | Transcription regulation |
| Q9VP47 | TSR1 | 1 | TSR1, 20S rRNA accumulation | Nucleus | other | Ribosome biogenesis |
| P25992 | UBN2 | -1 | ubinuclein 2 | unknown | other | Egg organization/transcriptional regulator |
| Q9VLT5 | UBR4 | -1 | Ubiquitin protein ligase E3 component n-recognin 4 | Nucleus | other | Differentiation/Neurogenesis |
| P51592 | UBR5 | 0 | Ubiquitin protein ligase E3 component n-recognin 5 | Nucleus | enzyme | Ubl conjugation pathway |
| Q86B79 | UNK | 1 | Unkempt homolog (*Drosophila*) | Nucleus | transporter | Essential for late larval and early pupal development |
| Q24574 | USP47 | 1 | Ubiquitin specific peptidase 47 | Cytoplasm | peptidase | Ubl conjugation pathway |
| Q9NHV9 | VAV1 | -1 | Vav 1 guanine nucleotide exchange factor | Nucleus | transcription regulator | Actin filament organization |
| Q24143 | HR96 | 0 | Nuclear hormone receptor HR96 | Nucleus | transcription regulator | RNA polymerase II regulatory region sequence-specific DNA binding |
| Q9W2F2 | VPRBP | 1 | Vpr (HIV-1) binding protein | Nucleus | other | Phosphoprotein |
| Q8T088 | WDR55 | 1 | WD repeat domain 55 | Nucleus | other | WD repeat WDR55 family |
| Q8MT36 | WHSC1 | -1 | Wolf-Hirschhorn syndrome candidate 1 | Nucleus | enzyme | Histone methyltransferase |
| P28466 | WNT5B | -1 | Wingless-type MMTV integration site family, member 5B | Extracellular space | other | Wnt signaling pathway |
| Q9Y0H4 | WWP1 | 1 | WW domain containing E3 ubiquitin protein ligase 1 | Cytoplasm | enzyme | Notch signaling pathway/Ubl conjugation pathway |
| Q9V3H9 | ZC3H14 | 0 | Zinc finger CCCH-type containing 14 | Nucleus | other | Phagocytosis, engulfment |
| Q9W0E8 | ZER1 | 0 | Zer-1 homolog | unknown | enzyme | Ubl conjugation pathway |

*Protein IDs uploaded into IPA were converted to the corresponding Entrez Genes in human ortholog for display in pathways and networks. Ingenuity's knowledge base contains published findings for both genes and proteins. However, for display purposes, only gene names are used, even when proteins IDs are uploaded.

^†^Total protein identifiers: 269 proteins (No matched proteins in the IPA database: 370 proteins). Over-presented proteins (detected only in pupae B reared on diet B): 89 proteins (no matched: 150), under-presented proteins (detected only in pupae A reared on diet A): 98 proteins (no matched: 135), overlap proteins: 82 proteins (no matched proteins: 150 proteins).
