# Supplementary material for: Diet-Induced Over-Expression of Flightless-I Protein and Its Relation to Flightlessness in Mediterranean Fruit Fly, Ceratitis capitata
Source: PLoS One. 2013 Dec 3;8(12):e81099. doi: 10.1371/journal.pone.0081099 (PMC3849048; doi:10.1371/journal.pone.0081099)
Supplement: Table S6 — Over-expressed proteins in pupae B reared with the liquid diet, which were identified by MALDI-TOF/TOF and de novo sequence analysis. (DOC) [file pone.0081099.s006.doc]

**Supporting Information (SI)**

**Diet-induced over-expression of flightless-I protein and its relation to flightlessness in Mediterranean fruit fly, *Ceratitis capitata***

Il Kyu Cho^1^, Chiou Ling Chang^2^ and Qing X. Li^1^*

^1^ Department of Molecular Biosciences and Bioengineering, University of Hawaii, Honolulu, Hawaii, USA.

^2^ U.S. Pacific Basin Agricultural Research Center, Hilo, Hawaii, USA.

**Table S6. Over-expressed proteins in pupae B reared with the liquid diet, which were identified by MALDI-TOF/TOF and de *novo sequence* analysis.**

| Spots | Protein names | A | B | C | D | E | Peptide sequences (Highest ion score) |
| --- | --- | --- | --- | --- | --- | --- | --- |
| 1 | Flightless-I | Q24020 | 143,682 | 5.61 | 13 | 20 | ATSLEFY IDFSLQTQLR (36) |
| 2 | Integrator complex subunit 3 | B4LQY8 | 125,417 | 6.65 | 6 | 20 | LSETGYLLLYFMK (58) |
| 5 | Spectrin beta chain | Q00963 | 265,575 | 5.57 | 8 | 20 | DVLGRILEK (48) |
| 6 | Paramysosin, long form | P35415 | 102, 338 | 5.47 | 5 | 30 | EAAESNLQVSER.K (50) |
| 7 | Leucine-rich repeat protein soc-2 | B5DX45 | 68,544 | 5.52 | 10 | 20 | NLALNENSLTSLPESLQNCK (60) |
| 8 | Coiled-coil domain-containing protein 22 | Q8l145 | 65,688 | 5.87 | 5 | 20 | NDLERWGDLPSMDPVTLTSTSDDLVGK (49) |
| 10 | 39 kDa FK506-binding nuclear protein | P54397 | 39,344 | 4.69 | 8 | 30 | SFHISGVALDK (68) |
| 11 | Ubiquitin-conjugating enzyme E2Q2 | Q32L27 | 38,864 | 4.86 | 4 | 13 | EKEGIEYILLNFSFKDNFPFDPPFVR (67) |
| 12 | V-type proton ATPase subunit d 1 | Q9W4P5 | 40,396 | 4.77 | 4 | 14 | TVAEYYAEYAALFDGSGNNPGDKTLEDK (110) |
| 13 | Protein hunchback | O46250 | 19,367 | 6.46 | 4 | 31 | ATAVATALQTGDKLQLTPPMDVTPPK (38) |
| 15 | Long epsilon-dendrotoxin | Q7LZE3 | 6,957 | 9.39 | 2 | 44 | LPAEPGPCKAsiPAFYYNWAAK (58) |
| 16 | Accessory gland protein | O46204 | 5,123 | 9.30 | 5 | 30 | ISALLKAVTK (42) |
| 17 | Slowpoke-binding protein | Q8IPH9 | 58,458 | 9.12 | 2 | 10 | TGVLPAPYE (78) |
| 18 | Cysteine and histidine-rich protein1 | Q9VZV5 | 46,221 | 7.55 | 5 | 15 | DPHQSNERTITYQLILK (120) |
| 19 | Leucine-rich repeat-containing G protein-coupled receptor 2 | Q9BN18 | 119,177 | 9.35 | 2 | 10 | VVGGGGPGGRGAVARTK (50) |
| 20 | Trifunctional purine biosynthetic protein adenosine-3 | Q26255 | 149,009 | 8.54 | 2 | 30 | GDAETTsiSYK (45) |
| 21 | E3 ubiquitin-protein ligase Su (dx) | Q9Y0H4 | 107,966 | 6.13 | 5 | 20 | SPLPNGGGDHR (70) |
| 22 | Protein wing apart-like | Q9W517 | 185,043 | 6.09 | 7 | 30 | KLGGLE (38) |

*A: Accession number, B: Calculated mass, C: Calculated pI, D: Number of matched peptides, E: Sequence coverage (%)
