# Supplementary material for: Diet-Induced Over-Expression of Flightless-I Protein and Its Relation to Flightlessness in Mediterranean Fruit Fly, Ceratitis capitata
Source: PLoS One. 2013 Dec 3;8(12):e81099. doi: 10.1371/journal.pone.0081099 (PMC3849048; doi:10.1371/journal.pone.0081099)
Supplement: Figure S1 — Overlay of extracted LC-ITMS total ion chromatograms of tryptic peptides from proteins in the second gel slice from pupae A and B (Ia and Ib, respectively), MS (IIa) and MS/MS (IIb) spectra of a fli-I tryptic peptide, and 1D SDS-PAGE image of proteins extracted from pupae A (IIIa) and B (IIIb). (DOC) [file pone.0081099.s007.doc]

**Supporting Information (SI)**

**Diet-induced over-expression of flightless-I protein and its relation to flightlessness in Mediterranean fruit fly, *Ceratitis capitata***

Il Kyu Cho^1^, Chiou Ling Chang^2^ and Qing X. Li^1^*

^1^ Department of Molecular Biosciences and Bioengineering, University of Hawaii, Honolulu, Hawaii, USA.

^2^ U.S. Pacific Basin Agricultural Research Center, Hilo, Hawaii, USA.

1

**b**MW

(KDa)

a

**III**


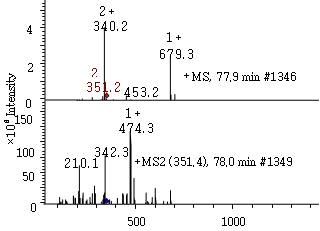


0

20

40

60

80

100

0

1

2

3

120 min

**Ia**

×10^6^ Intensity

*m/z*

**Ib**

**IIa**

75

119

**IIb**


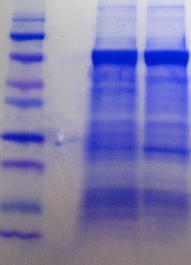


2

6

12

17

29

49

13

10

19

39

Marker

**Figure S1. Overlay of extracted LC-ITMS total ion chromatograms of tryptic peptides from proteins in the second gel slice from pupae A and B (Ia and Ib, respectively), MS (IIa) and MS/MS (IIb) spectra of a fli-I tryptic peptide, and 1D SDS-PAGE image of proteins extracted from pupae A (IIIa) and B (IIIb).** The horizontal lines in III indicate the slices excised for in-gel digestion.
