# Supplementary material for: Diet-Induced Over-Expression of Flightless-I Protein and Its Relation to Flightlessness in Mediterranean Fruit Fly, Ceratitis capitata
Source: PLoS One. 2013 Dec 3;8(12):e81099. doi: 10.1371/journal.pone.0081099 (PMC3849048; doi:10.1371/journal.pone.0081099)
Supplement: Figure S2 — Images of 2D GE of proteins extracted from pupae A (A) and B (B). (DOC) [file pone.0081099.s008.doc]

**Supporting Information (SI)**

**Diet-induced over-expression of flightless-I protein and its relation to flightlessness in Mediterranean fruit fly, *Ceratitis capitata***

Il Kyu Cho^1^, Chiou Ling Chang^2^ and Qing X. Li^1^*

^1^ Department of Molecular Biosciences and Bioengineering, University of Hawaii, Honolulu, Hawaii, USA.

^2^ U.S. Pacific Basin Agricultural Research Center, Hilo, Hawaii, USA.

1

22

KDa

140

3

21

2

19

20

**
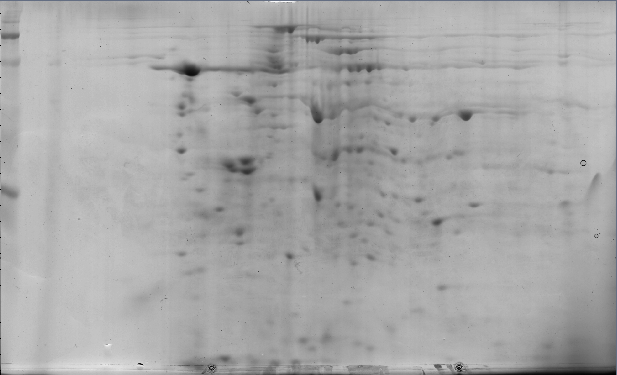

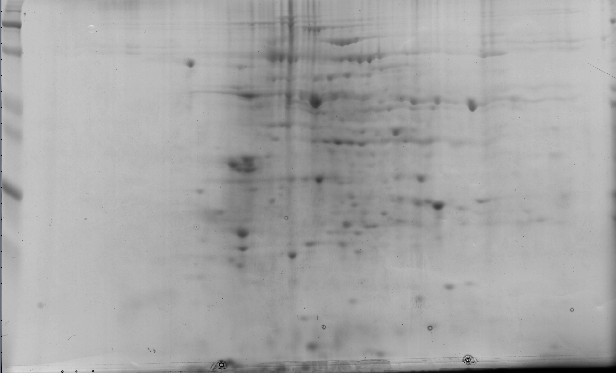
**

**A**

**B**

5

6

9

4

pH 3

pH 3

pH 10

pH 10

8

15

7

16

20

10

12

13

14

17

18

11

**Figure S2. Images of 2D GE of proteins extracted from pupae A (A) and B (B).** fli-I (spot 1), Hsp70Ab (spot 2), paramysosin (spot 6), LRR protein soc-2 (spot 7), LRR containing G protein-coupled receptor 2 (spot 19), E3 ubiquitin-protein ligase Su (spot 21) and protein wing apart-like (spot 22).
