# Supplementary material for: Diet-Induced Over-Expression of Flightless-I Protein and Its Relation to Flightlessness in Mediterranean Fruit Fly, Ceratitis capitata
Source: PLoS One. 2013 Dec 3;8(12):e81099. doi: 10.1371/journal.pone.0081099 (PMC3849048; doi:10.1371/journal.pone.0081099)
Supplement: Figure S3 — Stacked spectra of L-leucine standard (260 ppm) in water and extracts of pupae A (control) and pupae B which the latter showed a low flight rate. (DOC) [file pone.0081099.s009.doc]

**Supporting Information (SI)**

**Diet-induced over-expression of flightless-I protein and its relation to flightlessness in Mediterranean fruit fly, *Ceratitis capitata***

Il Kyu Cho^1^, Chiou Ling Chang^2^ and Qing X. Li^1^*

^1^ Department of Molecular Biosciences and Bioengineering, University of Hawaii, Honolulu, Hawaii, USA.

^2^ U.S. Pacific Basin Agricultural Research Center, Hilo, Hawaii, USA.

**Determination of L-Leucine with Fourier Transform Infrared Spectroscopy (FTIR).**

A Nicolet 6700 spectrometer (Thermo Electron Corp., Madison, WI, USA) equipped with a deuterated triglycine sulphate detector was used. The sampling station was equipped with a Smart Attenuated Total Reflectance Kit (ARK) (Thermo Electron Corp.) which is an advanced multibounce horizontal attenuated reflectance (HATR) accessory that is highly sensitive and provides high energy. The accessory comprised of a ZnSe crystal for the sample containment with an aperture angle of 45 °C, refractive index of 2.4 at 1000 cm^-1^, producing 12 reflections with a penetration depth (infrared beam) of 2.0 µm. The pupal samples were poured onto the HATR cell and then brought to 22 °C prior to spectral collection. Single beam spectra (400-4000 cm^-1^) of the samples were obtained against distilled water as background were collected by using rapid scan software running under OMNIC (Nicolet, Madison, WI, USA). The HATR crystal was carefully cleaned with distilled water and 75% ethanol between measurements. Each experiment was replicated 3 times and the spectra were averaged before subjecting to multivariate analysis. The FTIR calibration set comprised of 30 solutions of standards varying L-leucine concentrations for external validation. A region between 900 and 1500 cm^–1^ showed less interference with L-leucine peaks, a high correction between the peak intensity and L-leucine concentration (R^2^, 0.999), smallest root mean press value (RMPRESS) (0.106) and smallest standard error of calibration (SEC) (2.685), when different IR regions were compared (SI Figure 3, Table below). Therefore, the region 900-1500 cm^-1^ was selected for L-leucine analysis in the present study.

Model calibration and validation statistics for FTIR analysis of L-leucine content in mefly samples

| Range (cm^-1^) | Factors | RMPRESS | R^2^ | SEC |
| --- | --- | --- | --- | --- |
| 900-1150 | 5 | 0.385 | 0.998 | 3.579 |
| 900-1400 | 5 | 0.275 | 0.998 | 3.818 |
| 900-1900 | 6 | 0.106 | 0.999 | 2.685 |
| 900-1900,  3100-3200 | 7 | 0.433 | 0.998 | 3.637 |

Factors: optimum number of factors extracted

**Interpretation of L-leucine FTIR spectrum**

Functional groups and bond vibrations are responsible for IR spectra [1-4]. Functional groups leading to characteristic IR spectra of L-leucine include (a) methyl group, (b) amino group, (c) carboxyl group (SI Figure 3). OH, C=O and C-O stretching can exhibit strong IR absorption in the regions around 3000, 1700, 1300 cm^-1^, respectively [2]. The methyl group characterized by aliphatic CH_3_ bending can be accounted for strong IR absorption bands in a region around 2800-3200 cm^-1^ [3]. A strong IR band cluster often occurs in regions of 1020-1250 cm^-1^ and 1590-1627 cm^-1^, being attributed to C-N skeletal vibraton and RNH_2_ bending, respectively [4].

FTIR analysis showed 0.23 and 0.25 µg/g (wet weight) of L-leucine in pupae A (control) and pupae B, respectively. The results agreed well with those measured with a liquid chromatograph-mass selected detector (LC-MSD), which the L-leucine concentrations were 0.21±0.01 and 0.23±0.01 mg/g (wet pupae body weight) in pupae A and B, respectively.

**References**

(1) Kačuráková, M.; Mathlouthi, M. *Carbohydrate Research* **1996**, 284, 145-157.

(2) Pouchert, C. J. *Aldrich Library of FT-IR spectra*; Aldrich Chemical Co. Inc. 1986, I, 279-396.

(3) Colthup, N. B.; Daly, L. H.; Wiberley, S. E. *Introduction to Infrared and Raman Spectroscopy, 3^rd^ ed*; Academic Press: New York, **1990**.

(4) Lin-Vein, D.; Colthup, N; Fateley, W. G.; Grasselli, J. T*he Handbook of Infrared and Raman Characteristic Frequencies of Organic Molecules*; Academic Press: San Diego, CA, **1991**.

0.10

0.20

0.00

0.05

0.00

0.05

1000

1200

1400

1600

1800

2000

Wave numbers (cm-1)

Absorbance

**L-Leucine standard**

**Pupae A**

**Pupae B**

**Figure S3.** Stacked spectra of L-leucine standard (260 ppm) in water and extracts of pupae A (control) and pupae B which the latter showed a low flight rate.
