# Supplementary material for: Diet-Induced Over-Expression of Flightless-I Protein and Its Relation to Flightlessness in Mediterranean Fruit Fly, Ceratitis capitata
Source: PLoS One. 2013 Dec 3;8(12):e81099. doi: 10.1371/journal.pone.0081099 (PMC3849048; doi:10.1371/journal.pone.0081099)
Supplement: Figure S4 — LC-fluorescence chromatograms of L-leucine in medfly pupal extracts. (DOC) [file pone.0081099.s010.doc]

**Supporting Information (SI)**

**Diet-induced over-expression of flightless-I protein and its relation to flightlessness in Mediterranean fruit fly, *Ceratitis capitata***

Il Kyu Cho^1^, Chiou Ling Chang^2^ and Qing X. Li^1^*

^1^ Department of Molecular Biosciences and Bioengineering, UniverSIty of Hawaii, Honolulu, Hawaii, USA.

^2^ U.S. Pacific Basin Agricultural Research Center, Hilo, Hawaii, USA.

Pupae whose adult medflies had a flight rate of 38%.

Pupae whose adult medflies had a flight rate of 45%.

Control pupae (flight rate: >95%)

**Figure S4.** **LC-fluorescence chromatograms of L-leucine in medfly pupal extracts.** LC conditions: L-Leucine was eluted on an Atlantis HILIC silica column (4.6 x 250 mm, 5 μm) with 40 mmol/L Na_2_HPO_4_ solution at pH 7.8 for 6 min followed by a mixture of 45% acetonitrile, 45% methanol and 10% water for 4 min at a flow rate of 2 mL/min, and detected at λ_Ex_ 480 nm and λ_Em_ 560 nm; The injection volume was 10 μL. Different batches of medfly samples were used for the analysis of L-leucine content. The extracts were cleaned up with C8 SPE.
